# Synthesis, Properties, and Metathesis Activity of Polyurethane Thermoplastics and Thermosets from a Renewable Polysesquiterpene Diol

**DOI:** 10.1021/acs.macromol.4c02436

**Published:** 2025-08-01

**Authors:** Carli B. Kovel, Hannah Perine, Paul J. Chirik, Megan Mohadjer Beromi

**Affiliations:** † Department of Chemistry, 6740 Princeton University, Princeton, New Jersey 08544, United States; ‡ Department of Chemistry, 32722 United States Naval Academy, Annapolis, Maryland 21402, United States

## Abstract

Polyurethanes (PUs) are the sixth most commonly utilized plastic class, yet ∼80% of commodity material is landfilled or incinerated at the end of life. Disposal of thermosets is particularly problematic as cross-linking prevents the repurposing of disposed material. Thus, there is considerable interest in the development of PUs derived from inexpensive feedstocks that can be inherently chemically deconstructed. Ring opening metathesis polymerization (ROMP) of the naturally occurring sesquiterpene β-caryophyllene in the presence of dihydroxy chain terminators afforded the polyol hydroxy-terminated polycaryophyllene (HTPCR). Incorporation of HTPCR into PUs through reaction with polyisocyanates produced polymers with thermal and rheological properties comparable to commodity materials. The feasibility of chemical degradation of both thermoplastic and thermoset materials was also demonstrated through ruthenium-mediated metathesis, utilizing the metathesis-active olefins within the repeat caryophyllene monomer unit. Overall, this work highlights the value of biorenewable, chemically reprocessable polysesquiterpenes in the PU space.

## Graphical abstract

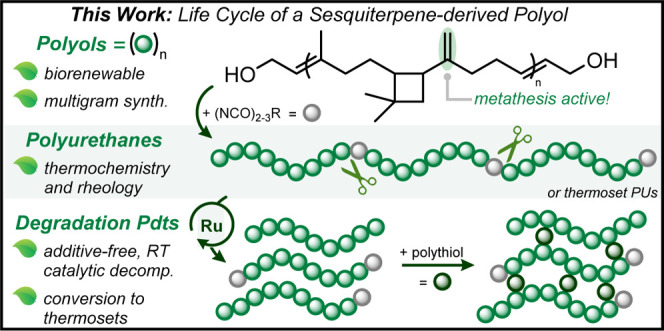

## Introduction

Polyurethanes (PUs) are a diverse class of polymers derived from the condensation of polyols and polyisocyanates.^1,2^ Given the vast array of structures that can be prepared from these reactions, the bulk properties, and thus applications, of PUs vary widely.^3^ Flexible PUs are utilized in packing foams and furniture, while rigid PUs have applications in insulation and construction.^4,5^ Other PU morphologies are suitable for use in coatings and adhesives due to their relatively high chemical and environmental stability.^6^ In total, these commodity PUs encompass approximately 8% of all plastics produced.^7,8^

The commercialization of PUs has led to an ever-increasing accumulation of waste material. Approximately half of end-of-life PU materials are disposed of by landfilling. Incineration is also utilized for approximately 30% of PU waste, while the remaining 20% of PU materials are recycled (Figure 1A).^7,8^ In the latter, mechanical recycling dominates, in which discarded PUs are reprocessed or rebonded and utilized in lower-value sectors, e.g., as filling for cushions.^9–11^ However, the ability to use postconsumer PUs for this purpose is limited by the additives contained within the PU formulations. An alternative strategy is chemical recycling, in which the bonds within the polymer chains are cleaved to afford feedstock chemicals that can be repolymerized, ideally, without loss of value.^12–17^ Stoichiometric chemical recycling of PUs targets polymer scission through stoichiometric glycolysis,^18,19^ hydrolysis,^20^ aminolysis,^21^ and other methods.^22–26^ Of these strategies, only glycolysis finds use in large scale applications due to the high cost, high energy requirements, and high waste generation that generally accompany these processes.^7,27^

**Figure 1:**
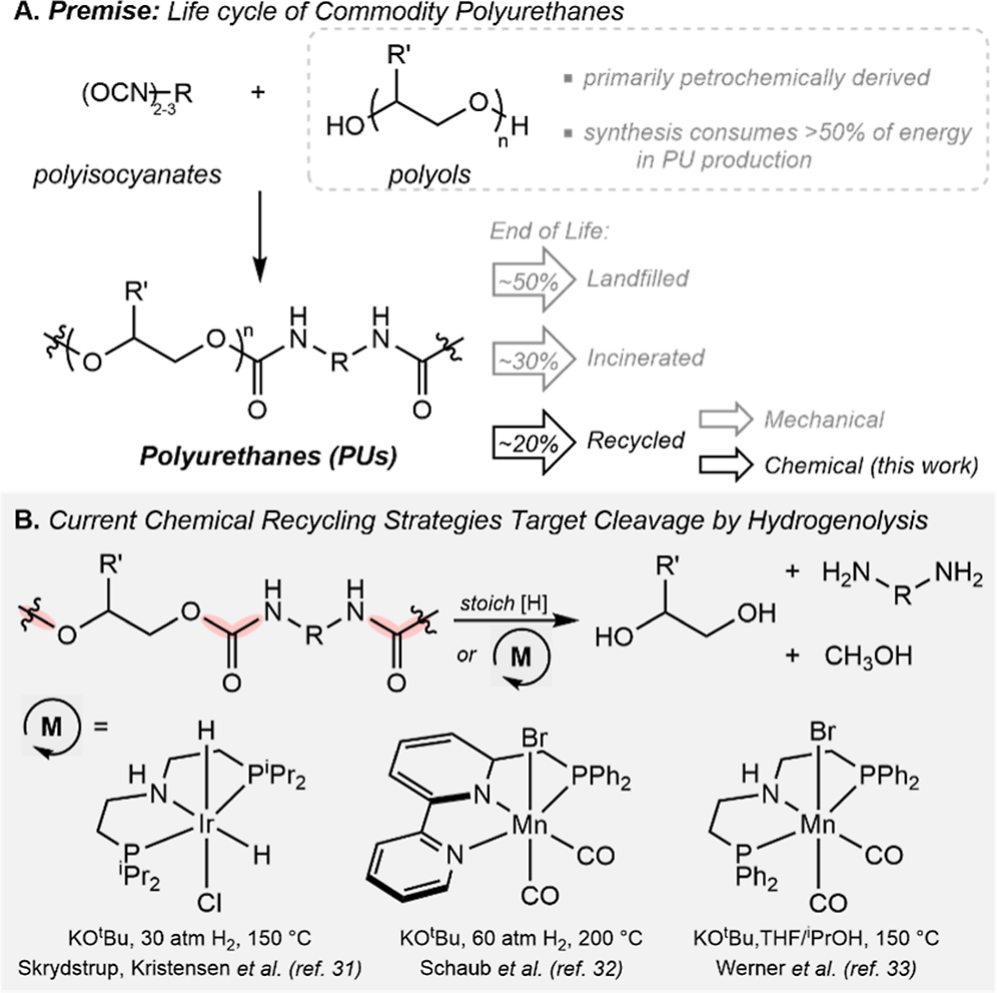
(A) Synthesis and end-of-life fate of commodity PUs. (B) Prior art in metal-mediated hydrogenolysis as a chemical recycling strategy for commodity PUs.

Catalytic, metal-mediated chemical recycling can address shortcomings associated with stoichiometric chemical cleavage of PUs.^28–30^ Seminal work in transition-metal catalyzed depolymerization of commodity PUs have utilized hydrogenolysis via attack by in situ-generated metal hydrides. The groups of Skrydstrup and Kristensen,^31^ as well as Schaub,^32^ have demonstrated the ability of pincer-ligated Ir, Fe, Mn, and Ru catalysts to convert PUs to their corresponding polyanilines and polyols in conjunction with 30–60 atm of dihydrogen and base activators at 150–200 °C (Figure 1B). Liu and Werner also demonstrated transfer hydrogenation with isopropanol as the source of reducing equivalents at 150 °C with a Mn pincer catalyst.^33^ Notably, these instances of stoichiometric and metal-mediated mechanisms target chain scission at the carbamate functionalities.

While the above processes are effective for circulating commercial material, there is also significant interest in designing new polymeric structures that are engineered to be inherently chemically recyclable.^34–37^ The design and implementation of such polymer structures represents a goal of the U.S. Department of Energy’s Strategy for Plastics Innovation by 2030.^38^ In designing a polymer with an optimal depolymerization route, an ideal system would reduce the number of additives and minimize reaction conditions while utilizing only the latent functional groups in the polymer structure.^39^ A mechanism that meets these constraints is intramolecular metathesis, where intrachain vinyl groups trigger the polymer cleavage event without the addition of exogenous olefin coupling partner (Figure 2A). Proven effective in the solvent-free decomposition of polybutadienes^40^ and of dehydrochlorinated polyvinyl chlorides,^41^ this depolymerization route only requires the polymer itself and a suitable metathesis catalyst; however, this process has not been utilized with PUs.

**Figure 2:**
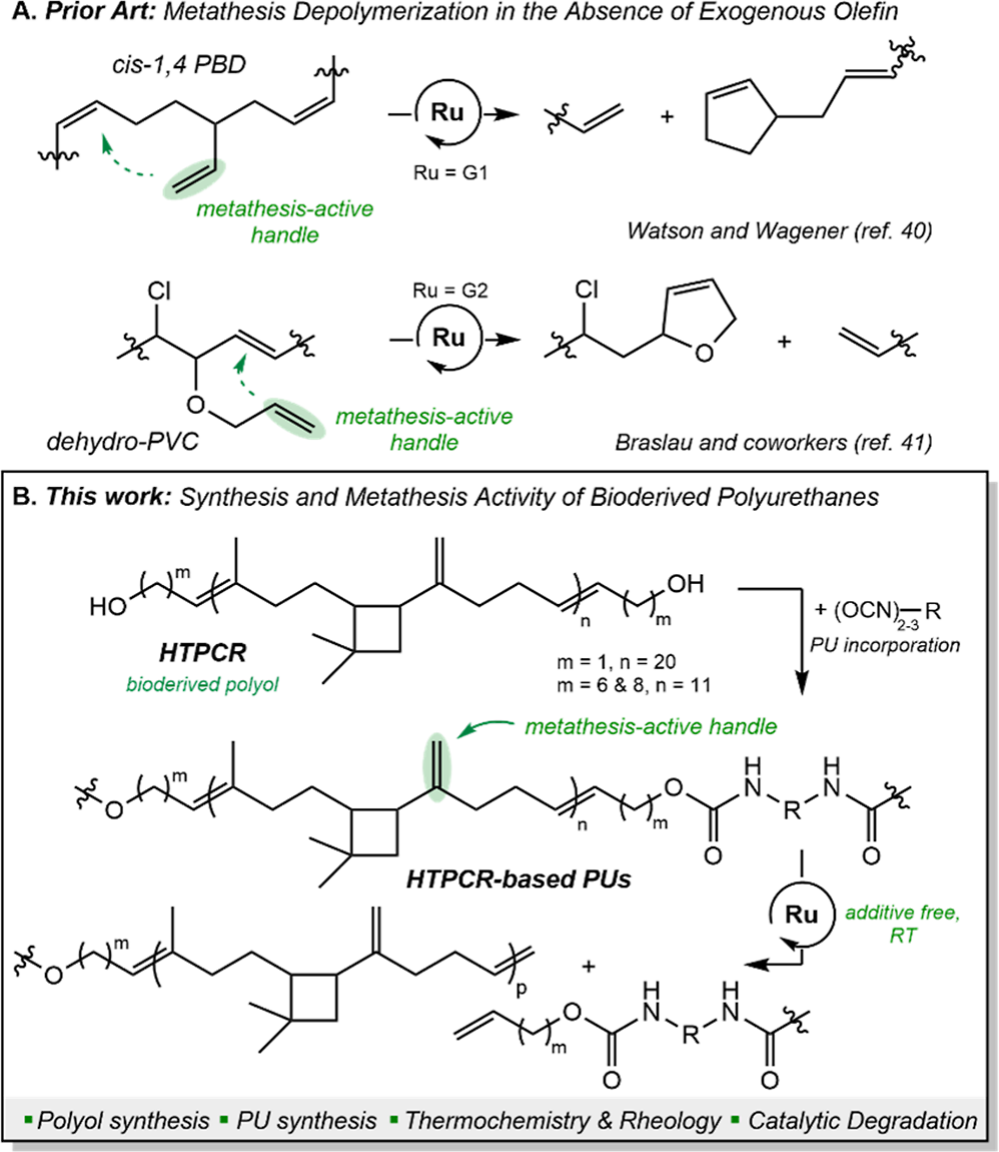
(A) Previously reported applications of intramolecular metathesis in the depolymerization of polybutadiene and dehydrochlorinated polyvinyl chloride. (B) This work: synthesis of HTPCR-based PUs and catalytic degradation via use of metathesis-active handles.

For the development of PU structures amenable to decomposition by intramolecular metathesis, the polyol linker provides a site for diversification. The most common polyols utilized in PUs are polyalkanoates.^42^ As linear aliphatic polyethers, these systems do not have the pendent olefinic functionalities necessary for metathesis depolymerization routes. In terms of environmental impact, these polyols are also primarily sourced from petrochemical feedstocks and account for high energy consumption in processing to PUs.^42–45^ In view of this, there is interest in bioderived polyol segments to address concerns with sustainability.^42–44,46,47^ For example, the lactone family has seen immense success in recent literature in the generation of chemically recyclable polyurethanes.^48–52^

The sesquiterpene β-caryophyllene, found in clove oil and presently produced on an industrial scale, has previously been polymerized by Grau and co-workers to polycaryophyllene.^53–55^ If incorporated into PUs as a polyol, this bioderived unit may be chemically deconstructed through metathesis originating from intrachain olefin functionalities (Figure 2B). Taken together, these polycaryophyllene-based PUs would not only be sourced from inexpensive, biorenewable feedstocks, but would also be inherently degradable without requisite stoichiometric additives.

Here we describe the synthesis and characterization of hydroxy-terminated polycaryophyllene (HTPCR) and demonstrate its thermal and rheological properties in representative PU thermoplastics and thermosets. Further, we demonstrate that the retained vinylidenes are metathesis-active functionalities in degradation pathways for the chemical downcycling of these PUs under mild reaction conditions with no exogenous additives. This work showcases the potential circularity of nonpetrochemically derived polyols in the PU resin class and highlights design principles in chemical reprocessability for their end-of-life fate.

## Results and Discussion

### Synthesis and Characterization of HTPCR

To develop a one-step procedure for the generation of HTPCR, direct chain end modification with diol chain transfer agents (CTAs) was investigated as a drop-in additive to the previously reported ring opening metathesis polymerization (ROMP) of β-caryophyllene by Grau and Mecking.^53^ In consideration of diol CTAs, Hillmyer and co-workers recently reported the synthesis of telechelic hydroxy-terminated polycyclooctene through the ROMP of cyclooctene in the presence of *cis*-7-hexadecene-1,16-diol.^56^ This bioderived CTA enabled the single step synthesis of molecular weight controlled hydroxy terminated polymers due to spacers between the alkene and hydroxyl groups, a feature that was hypothesized to also be requisite for HTPCR synthesis. Here, the polymerization of β-caryophyllene in the presence of *cis*-7-hexadecene-1,16-diol catalyzed by second-generation Grubbs catalyst ((1,3-bis­(2,4,6-trimethylphenyl)-2-imidazolidinylidene)­dichloro­(phenylmethylene)­(tricyclohexylphosphine)­ruthenium, G2) was conducted at 50 °C using the conditions detailed in Table 1 to afford a polyol identified as HTPCR­(6,8) (where the nomenclature (6,8) indicates the number of methylenes in the alcohol end group). After 24 h, an aliquot of the reaction mixture was examined by gel permeation chromatography (GPC) as well as by ^1^H NMR spectroscopy, with the latter indicating complete conversion of β-caryophyllene monomer. The number-average molecular weight (*M*_n_) determined by end-group analysis was in good agreement with the theoretical number-average molecular weight. Decreasing the ratio of β-caryophyllene to CTA successfully truncated the size of the HTPCR polymer chain obtained (Table 1, entries 2 and 3) while still affording complete conversion of the monomer and diol. The dispersities obtained under these conditions were also narrow at 1.2–1.6.

**Table 1:**
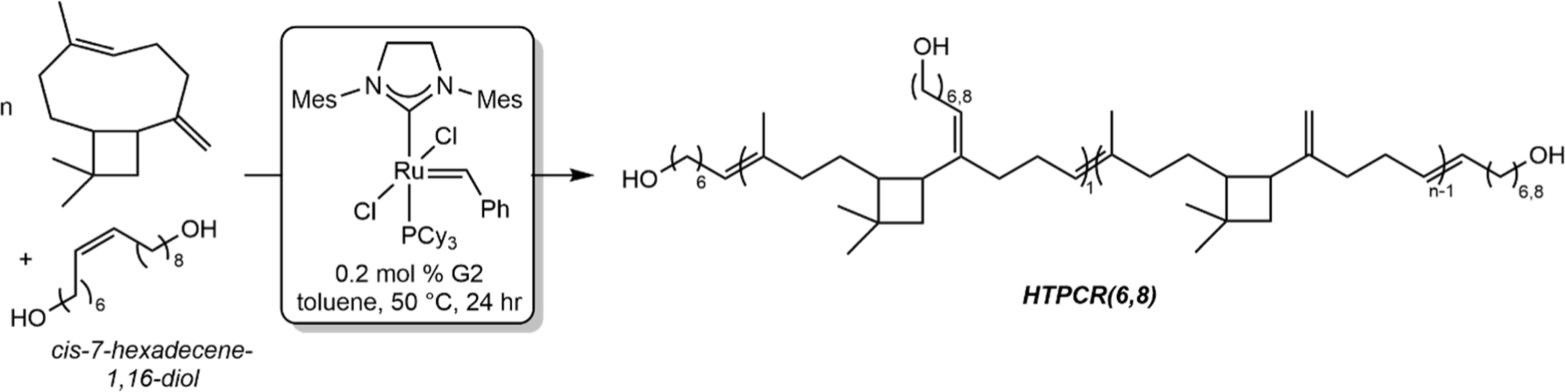
ROMP of β-Caryophyllene at Various Ratios of [Monomer]_0_/[CTA]_0_ Where CTA = *cis*-7-Hexadecene-1,16-diol^a^

entry	[monomer]_0_/[CTA]_0_	conv. (%)^b^	*M*_n_ (kg/mol) (theor)^c^	*M*_n_ (kg/mol) (NMR)^d^	*M*_n_ (kg/mol) (GPC)^e^	*Đ*
1	100	>99	20.7	26.1	15.6	1.6
2	30	>99	6.4	6.4	6.4	1.3
3	15	>99	3.3	3.0	2.8	1.2

a Polymerizations were conducted on a 1 g scale at 50 °C for 24 h, under an inert atmosphere in toluene. Catalyst loading was 0.2 mol % of G2.

b Conversion was determined by relative integration of olefinic protons using ^1^H NMR spectroscopy.

c Theoretical *M*_n_ was calculated with respect to ratio of monomer to CTA at full conversion.

d Determined by end-group analysis.

e GPC data was obtained with THF as the eluent. The values reported are relative to polystyrene standards.

With catalyst loadings of 0.2 mol %, the molecular weight was controlled by stoichiometric incorporation of the CTA into the polymer chain providing access to tunable molecular weight polymers. In the absence of CTAs, this catalyst loading would provide a theoretical average chain length of 500 units of caryophyllene monomer per polymer. Based on the lower molecular weights observed, lower amounts of ruthenium were used to access shorter polymer chain lengths. Based on these findings, the ruthenium catalyst must have re-engaged to synthesize additional polymer chains, decreasing the amount of ruthenium required for the polymer synthesis. Polyurethane properties are impacted by polyol chain length,^57^ prompting our synthesis of a range of molecular weights. The range of molecular weights achieved herein indicate potential for this strategy to yield a range of different materials with tunable properties. The direct correlation between increasing CTA to monomer ratio and decreased polyol molecular weight demonstrates the utility of this synthetic strategy in providing access to targeted molecular weights.

To further confirm the ^1^H NMR spectroscopic data supporting the incorporation of hydroxyl groups to the polymer chain ends as established by ^1^H NMR spectroscopy, the infrared (IR) spectrum of the resulting polymer was obtained; however, the –OH stretch expected in the range between 3550 and 3200 cm^–1^ was not observed (Figure S3C). This is likely a result of the low concentration of hydroxyl groups with respect to C–C and C–H bonds reducing their relative intensity in the resulting IR spectrum. As such, acetylation was chosen as a chemical method of corroborating the presence of hydroxyl groups in the polymer, in which HTPCR­(6,8) was treated with trifluoroacetic acid anhydride at ambient temperature in chloroform (Figure 3). After 20 min of reaction time, the ^1^H NMR spectrum indicated the disappearance of the methylene protons adjacent to oxygen at 3.36 ppm and the appearance of a signal corresponding to the methylene protons adjacent to trifluoroacetate at 3.76 ppm. The shift in signals verifies the presence of hydroxy groups on the parent HTPCR polymer. Determination of the degree of OH functionality was conducted using MALDI-TOF spectroscopy. Analysis of HTPCR­(6,8) indicated the expected distribution of molecular weights increasing stepwise by one caryophyllene repeat unit (Figure S3E). The masses identified are consistent with triple CTA incorporation for a total of 3 OH groups. Within each mass peak was also found a statistical distribution of chain end lengths; combinations of 6 and 8 methylene linkers account for the apparent triplets in mass peaks obtained. Notably, this implies that the vinylidene units are metathesis active in the presence of a suitable exogenous olefin.

**Figure 3:**
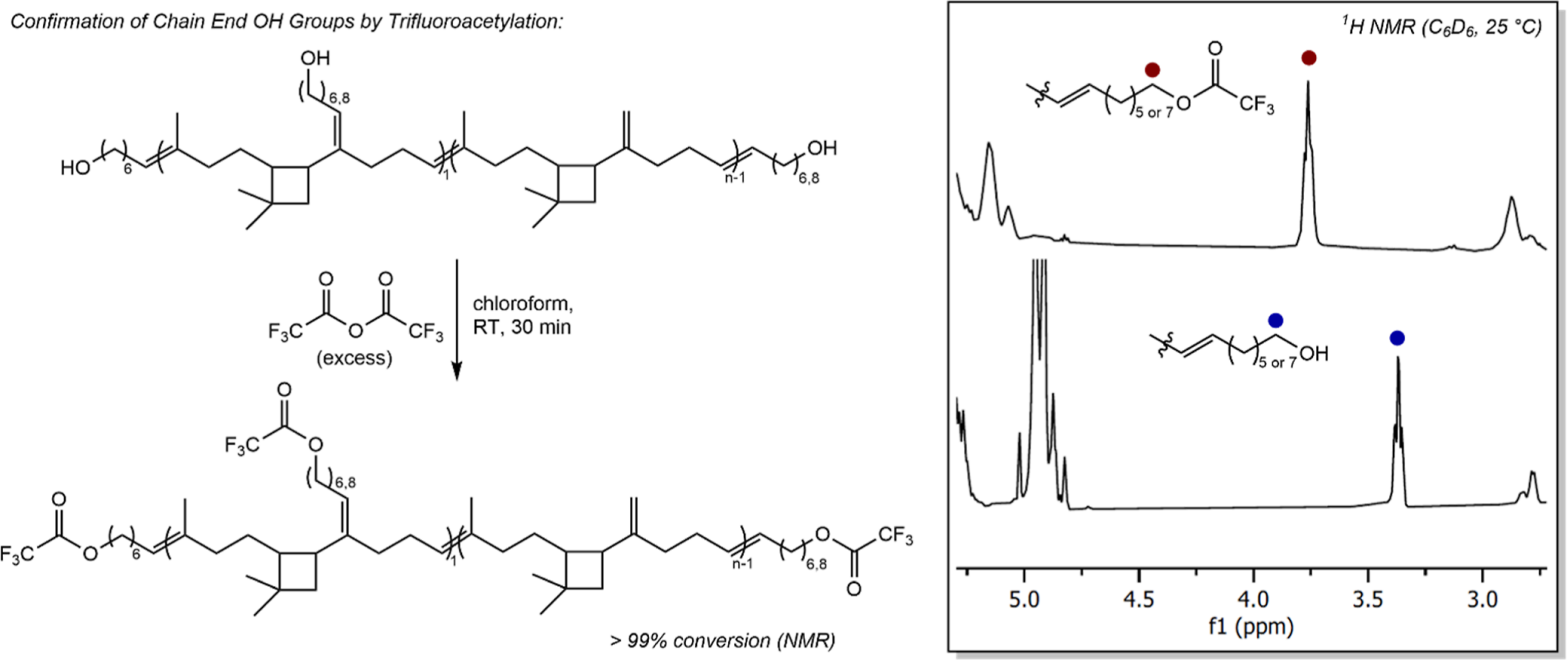
^1^H NMR spectra (C_6_D_6_, 25 °C) of HTPCR­(6,8) (bottom) and trifluoroacetylated HTPCR­(6,8) (top), with methylene groups adjacent to either the hydroxy or trifluoroacetyl group annotated.

To deconvolute the effects of the extended aliphatic, asymmetric chain ends on the HTPCR structure, direct hydroxylation with *cis*-2-butene-1,4-diol was attempted under conditions similar to those previously utilized (Table 2). Production of polyol HTPCR(1) was achieved but required higher ratios of monomer to CTA and lower catalyst loading (0.1 mol % G2). Even with the higher monomer ratio, decreased conversion was seen than that with *cis*-7-hexadecene-1,16-diol CTA (entries 1 and 2). Further decreasing the catalyst loading to 0.05 mol % and decreasing the availability of OH groups through control of monomer to CTA ratio allowed for full conversion (Table 2, entry 3) with *M*_n_ exceeding 20 kg/mol. However, aldehyde end groups were detected by both NMR and IR spectroscopies, suggesting isomerization of the chain ends; this has previously been observed by Grubbs and co-workers in the synthesis of hydroxy-terminated polybutadiene.^58^ To avoid the presence of aldehyde chain ends, HTPCR(1) was accessed from the ROMP of β-caryophyllene in the presence of *cis*-1,4-diacetoxy-2-butene, followed by subsequent deprotection to produce hydroxy-terminated polymer on scales upward of 40 g (*M*_n_ = 4.8 kg/mol, *Đ* = 1.5, Scheme 1). Analysis of the degree of OH functionality of HTPCR(1) by MALDI-TOF indicates that two OH groups are incorporated into the molecule, in contrast to that observed with the more active *cis*-7-hexadecene-1,16-diol (Figure S2F). It is hypothesized that the reduced reactivity of this CTA in the ROMP process also precludes it from reacting further with vinylidenes, thus resulting in a product with an OH functionality of two.

**Table 2:** ROMP of β-Caryophyllene with Varied [Monomer]_0_/[CTA]_0_ Where CTA = *cis*-2-Butene-1,4-diol^a^

entry	[monomer]_0_/[CTA]_0_	conv. (%)^c^	*M*_n_ (kg/mol) (theor)^d^	*M*_n_ (kg/mol) (exp.)^e^	*Đ*
1	100	29	10.2	5.4	1.2
2	300	33	30.6	7.3	1.1
3^b^	1000	>99^f^	24.0	23.7	1.4

a Polymerizations were conducted on a 1 g scale at 50 °C for 24 h, under an inert atmosphere in toluene. Catalyst loading was 0.1 mol % of G2.

b Polymerization conducted with 0.05 mol % G2.

c Conversion was determined by relative integration of olefinic protons using ^1^H NMR spectroscopy.

d Theoretical *M*_n_ was calculated with respect to ratio of monomer to CTA at full conversion.

e GPC data was obtained with THF as the eluent. The values reported are relative to polystyrene standards.

f Contained 2.5% aldehyde chain ends from isomerization.

**Scheme 1:**
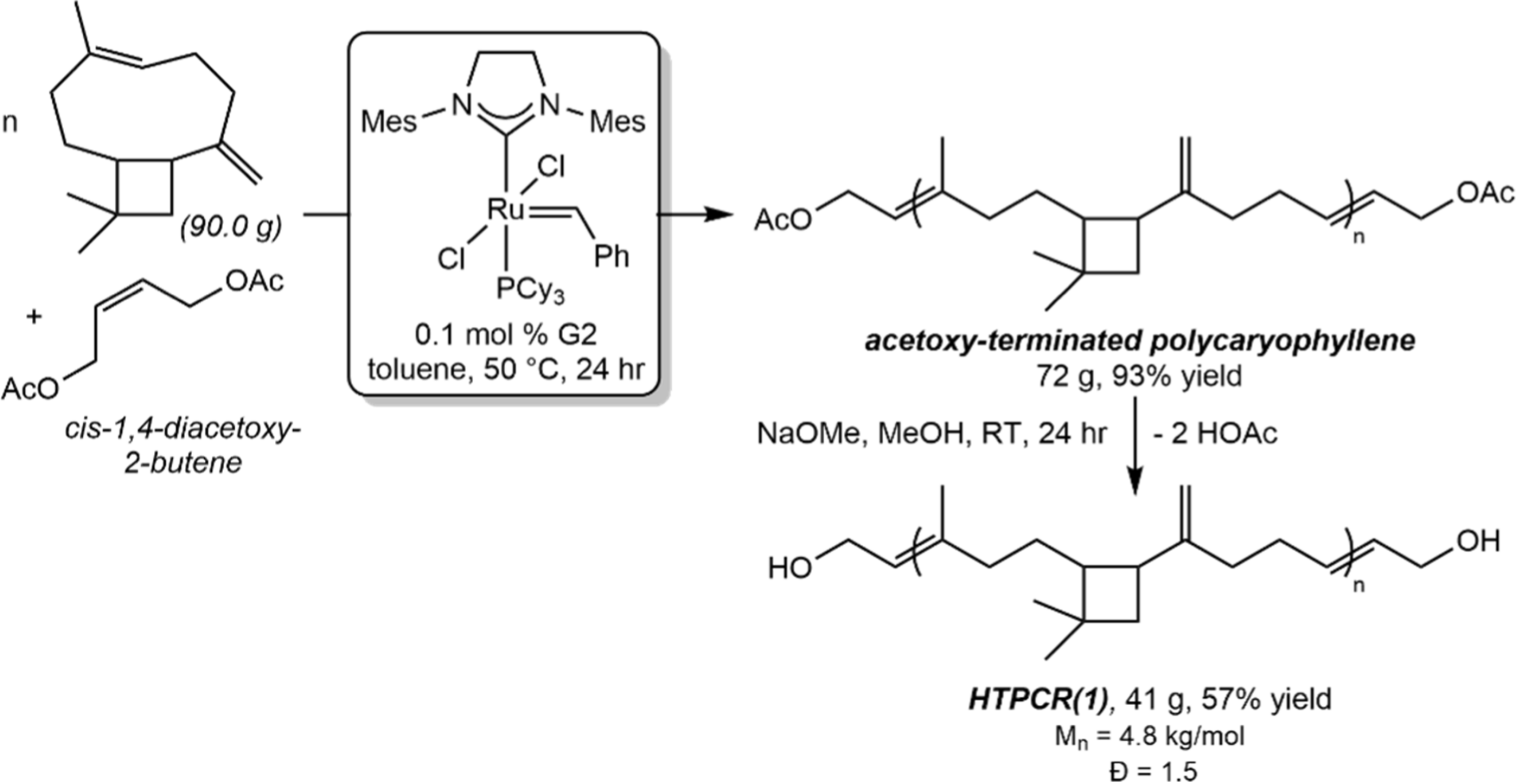
Synthesis of HTPCR(1) on 40 g Scale by Sequential Acetylation and Deprotection

The apparent metathesis activity of the exocyclic methylenes in HTPCR­(6,8) prompted further investigation of the microstructure of the polymers. Examination of the ^13^C NMR spectra of both HTPCR(1) and HTPCR­(6,8) revealed the presence of a signal at 108.3 ppm, consistent with the presence of a terminal vinyl group.^53^ Terminally vinylated HTPCR­(6,8) accounted for ∼17% of the total sample, while vinyl-terminated HTPCR(1) constituted ∼8% of the total sample (Table 3 and Figure S5). In HTPCR(1) and acetoxy-terminated polycaryophyllene, the bulk majority of the polymer is α,ω-substituted (>92%), with the remaining polymer microstructure arising from a different sequence of metathesis events arising from the interaction of the polymer and CTA with the propagating ruthenium alkylidene (see Section II. Assessment of Metathesis Processes Leading to Vinyl Termination in the Supporting Information). This finding is significant in that it demonstrates the potential utility of the vinylidene unit in further metathesis chemistry, particularly, in polymer chain cleavage (vide infra). Rigorous confirmation of the metathesis activity of the vinylidenes was obtained through the postsynthetic modification of HTPCR(1) with CTA under ROMP polymerization conditions. Here, superstoichiometric *cis*-1,4-diacetoxy-2-butene was reacted with a solution of HTPCR(1) and 0.2 mol % G2 in benzene-*d*_6_ at 50 °C for 24 h. Monitoring the reaction by quantitative ^13^C­{^1^H} NMR spectroscopy indicated a 12% reduction in vinylidene signals concomitant with an 8% increase in signals associated with the carbonyl carbon of the acetoxy group (Figure S6), verifying that the vinylidenes are indeed metathesis active.

**Table 3:**
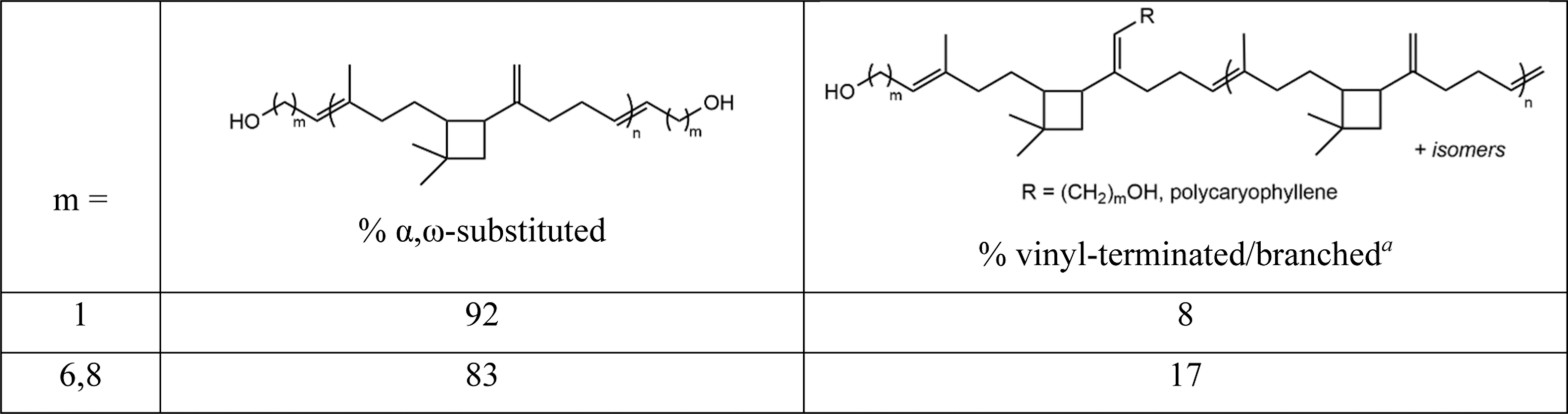
Percent α,ω-Substituted Microstructure in HTPCR(1) and HTPCR­(6,8) Quantitated by ^13^C NMR

a See Section II. Assessment of Metathesis Processes Leading to Vinyl Termination of the Supporting Information.

Thermal and rheological characterization of HTPCR(1) and HTPCR­(6,8) produced on multigram scale were conducted to further understand the properties of this polyol class (Table 4). Both HTPCR(1) and HTPCR­(6,8) are less dense and have higher dynamic viscosities than similar molecular weight polyethylene glycol polyols.^59,60^ Both HTPCR polyol variants exhibit low glass transition temperatures, as identified by DSC, while the decomposition temperatures are similar within ca. 10 °C. Overall, both variants exhibit high thermal stability, with decomposition events in TGA occurring at temperatures over 300 °C.

**Table 4:** Tabulated Thermal and Rheological Data for HTPCR(1) and HTPCR­(6,8) Prepared on Multigram Scale

	HTPCR(1)	HTPCR(6,8)
*M*_n_ (kg/mol)	4.8	2.8
*D̵*	1.5	1.2
ρ (g/cm^3^)^a^	0.8687	0.9123
ν (m^2^/s)^a^	0.0547	0.0236
μ (Pa·s)^a^	47.55	21.54
*T*_g_ (°C)	–33.5	–47.7
*T*_d_ (5%) (°C)	303	310

a Data obtained at 25 °C. Conditions for thermal characterization can be found in the Supporting Information.

### Synthesis and Characterization of HTPCR-Based PUs

In PUs, the polyol typically serves as the soft segment in the polymer chain, whereas modulation of rigid polyisocyanates controls the hard segments.^61^ Given the principally linear aliphatic composition of the HTPCR backbone, it was anticipated that the role of soft segment would be unchanged for this polyol. As such, toluene diisocyanate (TDI) was selected as a prototypical hard segment diisocyanate for the synthesis of a HTPCR-based thermoplastic.^62^ Given its characterization as rigorously a diol paired with its scalability of synthesis, HTPCR(1) was selected for the further generation of representative PU materials.

Condensation of HTPCR(1) with one equivalent of TDI in the presence of catalytic dibutyltin dilaurate (DBTDL) led to poor condensation at ambient temperatures; however, conducting the reaction at 110 °C for 24 h resulted in the production of a thermoplastic with adhesive-like properties identified at HTPCR-TDI (Scheme 2, top). Increasing the ratio of diisocyanate to HTPCR(1) caused the condensation polymerization to proceed at room temperature to afford a fully solid, translucent material (Scheme 2, top). Cast-curing into aluminum pans allowed for the fabrication of pucks from which dogbones were cut. After removal of excess unreacted isocyanate by sonication in acetone, the resulting thermoplastics were soluble in nonpolar organic solvents and were characterized by NMR and IR spectroscopies. In the benzene-*d*_6_ NMR spectrum, the methylene protons adjacent to the OH groups in parent HTPCR(1) shift from 4.00 to 4.70 ppm, analogous to the spectroscopic signatures of other phenyl propenyl carbamates.^63,64^ The urethane linkage itself was confirmed by IR spectroscopy through the identification of carbonyl groups with stretching frequencies around 1700 cm^–1^ as well as the lack of residual isocyanate stretch at ∼2300 cm^–1^. The solubility of these HTPCR-TDI formulations also enabled analysis of molecular weight by GPC, which indicated an *M*_n_ of 14.5–14.7 kg/mol obtained from the cast curing process. Given the similar molecular weights, the difference in morphologies is attributed to the different dispersities; the elastomer has a significantly lower dispersity of 2.2 compared to 3.1 for the adhesive. Thermochemical events for each material were elucidated through a combination of dynamic mechanical analysis (DMA), DSC, and TGA (Table 5). The HTPCR-TDI elastomer exhibits a glass transition (*T*_g_) at −19.9 °C, a melting temperature of 223 °C, and two decomposition events at 257 and 315 °C, with the latter paralleling that seen in the polyol.

**Scheme 2:**
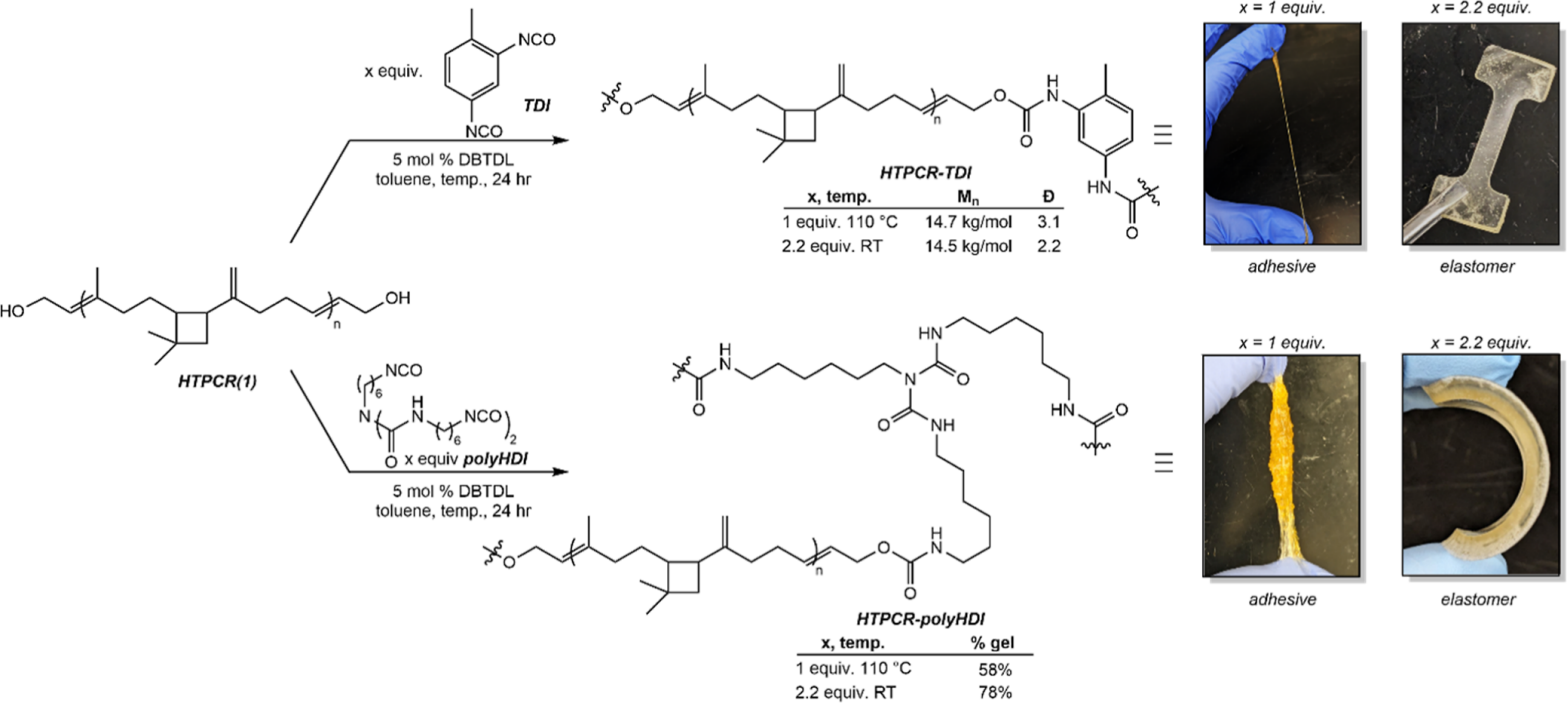
Synthesis of Thermoplastic HTPCR-TDI (Top) and Thermoset HTPCR-PolyHDI (Bottom)^a^

a DBTDL = dibutyltin dilaurate.

**Table 5:** Tabulated Thermal and Rheological Data for Synthesized HTPCR-Based Thermoplastic and Thermoset Polymers, with Synthesized HTPB-PolyHDI Provided for Comparison

	HTPCR-TDI (adhesive)	HTPCR-TDI (elastomer)	HTPCR-polyHDI (adhesive)	HTPCR-polyHDI (elastomer)	HTPB-polyHDI (elastomer)
*T*_g_ (°C)	–25.7^b^	–19.9^c^	–27.8^b^	–31.3^c^	–18.5
*T*_m_ (°C)	^d^	223	^d^		
*T*_d_ (5%) (°C)	261	257, 315^e^	278	256, 307^e^	283
Young’s modulus (MPa)^a^		4.382 ± 0.166		3.297 ± 0.071	1.300 ± 0.011
strain at break (%)^a^		150 ± 0.5		64.5 ± 7.5	62.4 ± 0.05
load at break (MPa)^a^		1.918 ± 0.079		0.522 ± 0.031	0.545 ± 0.004
lap shear strength (kPa)^a^	50.4 ± 15		213 ± 35		

a Reported as the average of at least two runs. Conditions for thermal characterization can be found in the Supporting Information.

b Obtained by DSC.

c Obtained by DMA.

d No melting events detected by DSC.

e Two thermal events identified.

Given the inclusion of exactly two hydroxy groups per HTPCR(1) chain, the synthesis of a representative PU thermoset material required condensation with a polyisocyanate with isocyanate functionality greater than two to ensure cross-linking. Here, HTPCR-polyHDI was synthesized through the cast curing of HTPCR and stoichiometric or excess poly­(hexamethylenediisocyanate) (polyHDI) in the presence of 5 mol % DBTDL (Scheme 2, bottom). Similarly to the TDI-based polyurethanes, condensation of HTPCR(1) with only one equivalent of polyHDI resulted in poor cross-linking unless conducted at elevated temperature, resulting in an adhesive material once more. Increasing the equivalents of polyHDI allowed cross-linking to occur at room temperature over 24 h to afford a flexible solid elastomer. Quantification of the gel fraction was conducted by extraction in THF at 50 °C for 24 h. Under these conditions, the adhesive material partially dissolved, resulting in only 58% of recovered cross-linked material. In contrast, the solid elastomer was treated in the same extraction process, under which 78% of the material was preserved, indicating a relatively high degree of cross-linking. This elastomeric material is characterized by a *T*_g_ of −31.3 °C, which is comparable to the HTPCR polyol prepolymer and significantly lower than that of the HTPCR-TDI thermoplastic (Table 5). The latter observation is attributed to the lack of hard segments present in the HTPCR-polyHDI thermoset, as neither the diol nor the polyisocyanate are rigid. Like the thermoplastic PU, HTPCR-polyHDI also exhibits thermal stability greater than 250 °C.

The adhesives obtained from stoichiometric reaction of polyisocyanates with HTPCR(1) were found capable of bonding roughened aluminum plates. Curing the HTPCR(1) and polyisocyanates at 110 °C between aluminum plates resulted in adhesion suitable for lap shear testing. Quantification of their bonding strength indicated an average lap shear strength of 50.4 ± 17 kPa for HTPCR-TDI adhesive and 213 ± 35 kPa for HTPCR-polyHDI adhesive (Tables 5 and S1, Figure S15). While these shear strengths are not currently comparable to optimized PU adhesives in circulation,^65,66^ the ability of these preliminary formulations to bond aluminum to any degree provides the first demonstration of the potential use of these materials for these purposes.

In contrast, the elastomers generated from higher ratios of polyisocyanate to HTPCR were cast-cured into dogbones amenable to tensile testing. The position of the dogbones was adjusted at a rate of 1.000 in. per minute at room temperature until a load of 21.35 N was achieved or breakage occurred. The combined tensile data is summarized in Table 5. HTPCR-TDI exhibited an average Young’s modulus of 4.382 ± 0.166 MPa, placing HTPCR-TDI in a similar elasticity profile as “soft” commercial thermoplastic polyurethane rubbers (PURs).^65,66^ Similarly, tensile testing of HTPCR-polyHDI indicated an average Young’s modulus of 3.297 ± 0.071 MPa. The thermoplastic elastomer possessed an approximately 2-fold enhancement in percent strain at break (150% vs 64.5%) and almost 4-fold enhancement in stress at break (1.918 MPa vs 0.522 MPa) compared to the thermoset, which can be attributed to the effects of cross-linking and chain mobility.

For more direct comparison to commodity PUs, a thermoset was prepared from the reaction hydroxy-terminated polybutadiene (HTPB, *M*_n_ = 1.2 kg/mol) with polyHDI under the same conditions as that of HTPCR-polyHDI elastomer; this polyol was chosen for comparison due to its ubiquity in commercial PU roles, including in propellants, adhesives, and coatings.^67–69^ The resulting thermoset was analyzed by identical thermal and rheological analysis as the HTPCR-based PUs, with the results reported in Table 5. When synthesized under the same conditions, the HTPCR-polyHDI elastomer has a significantly lower *T*_g_ (−31.3 °C vs −18.5 °C) yet slightly higher Young’s modulus at room temperature to that of HTPB-polyHDI; further, the thermosets possess strikingly similar elongation percentages (64.5% vs 62.4%) and load capacity profiles (0.522 MPa vs 0.545 MPa).

### Metathesis Activity of PU Thermoplastics and Thermosets

To investigate the potential of alkene metathesis as a method for the decomposition of the HTPCR-based PUs, metathesis reactions were attempted whereby the same catalyst used in HTPCR synthesis was applied to the soluble polymers. Homogeneous solutions of HTPCR(1) and HTPCR-TDI in THF were stirred with 1 wt % of G2 at ambient or elevated temperature for 48 h, with periodic monitoring by GPC (Table 6). Minimal to no decomposition or change in dispersity was observed in the HTPCR(1) polyol at either ambient or elevated temperature (Table 6, entries 1 and 2). Significantly, at 48 h of reaction at 50 °C, the molecular weight of the polymer increased from the 24 h time point; this result may imply that repolymerization pathways interfere with productive chain cleavage pathways at elevated temperature.

**Table 6:**
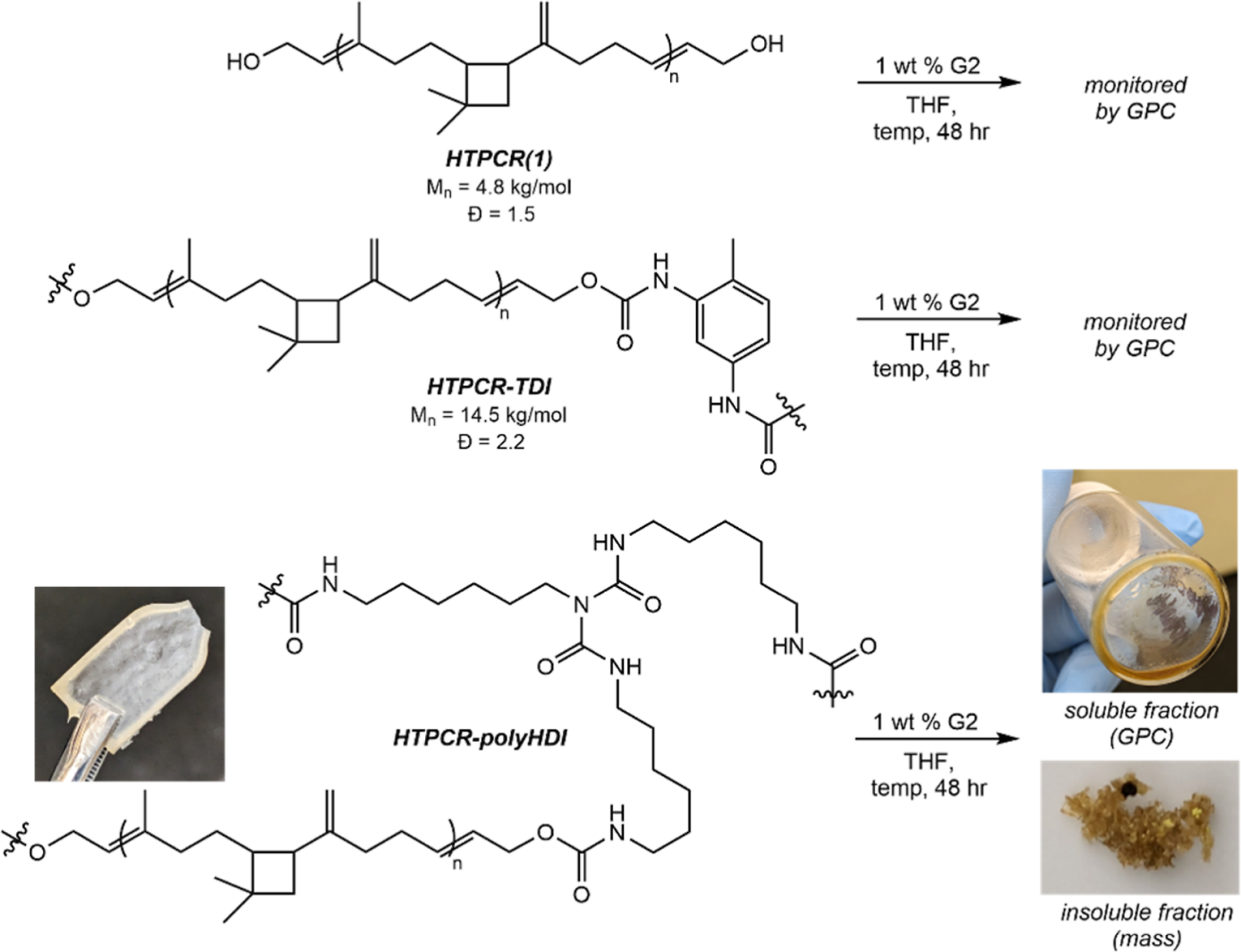
*M*_n_, Dispersity, and Yield Data for the Metathesis Degradation of HTPCR-Based Polyol, Thermoplastic, and Thermoset Polymers^a^ ^,^ ^b^

entry	polymer	time (h)	temp. (°C)	*M*_n_ (kg/mol)	*Đ*	percent solubilized^a^
1	HTPCR(1) *M*_n_ = 4.8 kg/mol *Đ* = 1.5	24	RT	4.2	1.6	
		48	RT	3.2	1.4	
2	HTPCR(1) *M*_n_ = 4.8 kg/mol *Đ* = 1.5	24	50	3.3	1.5	
		48	50	5.0	1.5	
3	HTPCR-TDI *M*_n_ = 14.5 kg/mol *Đ* = 2.2	24	RT	5.5	3.1	
		48	RT	5.0	3.4	
4	HTPCR-TDI *M*_n_ = 14.5 kg/mol *Đ* = 2.2	24	50	5.2	3.1	
		48	50	5.1	3.0	
5	HTPCR-polyHDI	24	RT	10.0	5.3	
		48	RT	9.9	5.4	95
6	HTPCR-polyHDI	24	50	3.3	5.3	
		48	50	4.3	7.1	42

a Conditions: 200 mg polymer, 1 wt % G2, 5 mL THF, inert atmosphere.

b Determined from isolation of insoluble material postreaction.

In contrast, HTPCR-TDI showed an approximately 60% decrease in *M*_n_ after 24 h at ambient temperature, with minimal decreases in molecular weight at prolonged reaction times (Table 6, entry 3). Increasing the temperature to 50 °C led to a similar molecular weight distribution as obtained from the ambient reaction conditions (Table 6, entry 4). The dispersities of the soluble material also increased significantly from that of the parent HTPCR-TDI thermoplastic elastomer, characteristic of olefin exchange and subsequent fragmentation of the polymer chain. Evaluation of other ruthenium catalysts in the decomposition did not lead to an increase in degree of decomposition (Table S2), and further reduction of catalyst loading led to decreased metathesis activity (Table S3). Control experiments omitting added metal catalyst resulted in no degradation observed (see Supporting Information for details).

Given the molecular weight reduction seen in the thermoplastic HTPCR-based PU, it was hypothesized that this process would translate onto the thermoset material as well. Such a procedure would provide a rare case of the metal-mediated chain scission of thermoset PUs, and one utilizing a distinct mechanism from previous hydrogenolysis reports. To this end, the HTPCR-polyHDI thermoset was shredded and suspended in a solution of 1 wt % G2 in THF. The heterogeneous mixture was stirred at ambient temperature or 50 °C for 48 h with periodic monitoring by GPC. Under ambient conditions, swelling and gelation of the mixture preceded dissolution of the thermoset fragments. Characterization of the molecular weight distribution of an aliquot of the solubilized material by GPC revealed a *M*_n_ of 10.0 kg/mol with broad dispersity (*Đ* = 5.3) after 24 h (Table 6, entry 5). Allowing the reaction to proceed for 48 h once again did not lead to significant decreases in *M*_n_ or improvements in dispersity. At the end of the reaction, the soluble material was separated from the insoluble solids by filtration, after which the solids were dried and weighed to recover 5% of the initial insoluble thermoset. Increasing the reaction temperature to 50 °C led to a significant truncation of polymer chain length, as indicated by the *M*_n_ of 3.3 kg/mol obtained after 24 h. However, this appears to come at the expense of percent decomposition, in which only 42% of the thermoset solubilized after 48 h at elevated temperature (Table 6, entry 6). Further, the *M*_n_ of the solubilized material increased from 24 to 48 h concurrent with a significant erosion in dispersity (*Đ* = 7.1), analogous to the phenomenon observed in the polyol decomposition at elevated temperature. This implies that random repolymerization and/or cross-linking may once again interfere or outcompete productive chain scission at elevated temperature. Nevertheless, these data indicate that both thermoplastic and thermoset HTPCR-based PUs are amenable to deconstruction through processes distinct from hydrogenolysis.

To investigate the dominant mechanism of chain scission, the metathesis reactions were monitored by ^1^H and ^13^C NMR spectroscopies. For the decomposition of the thermoplastic, a homogeneous solution of HTPCR-TDI and 3 wt % G2 was prepared in benzene-*d*_6_. To accelerate the process for monitoring by NMR spectroscopy, a higher catalyst loading and sonication were used, ensuring that the bath remained at ambient temperature. As indicated by the NMR spectra, no small molecule ring closing metathesis products were detected, nor was ethylene produced as a byproduct (Figure S21). Instead, the spectra remained largely unchanged except for the appearance of new olefinic signals identified at 108.3 and 125.3 ppm by ^13^C NMR spectroscopy, assigned to terminal vinyl groups in the polycaryophyllene fragment (Figure 4, full spectrum in Figure S21B). Further, the stereochemical information from the olefinic signal at 125.3 ppm was lost, supporting the assignment as signals belonging to terminal olefins. These signals are also consistent with that observed in independently synthesized polycaryophyllene.^53^ Paired with the lack of identification of caryophyllene monomer or related products by either NMR or gas chromatography/mass spectrometry (GC/MS) (Figures S21D and S23), these signals are consistent with the production of linear chain end vinyl groups. Signals congruent with the in-growth of propenyl carbamate chain termination from the other end of the cleaved polymer fragment were also identified (Figure S21A). Notably, signals consistent with vinylated TDI fragments were located at 131.3 ppm over the course of 12 h that subsequently reduce in intensity by the end of the timecourse experiment, presumably as the end vinyl group participates further in alkene metathesis cleavage reactions.

**Figure 4:**
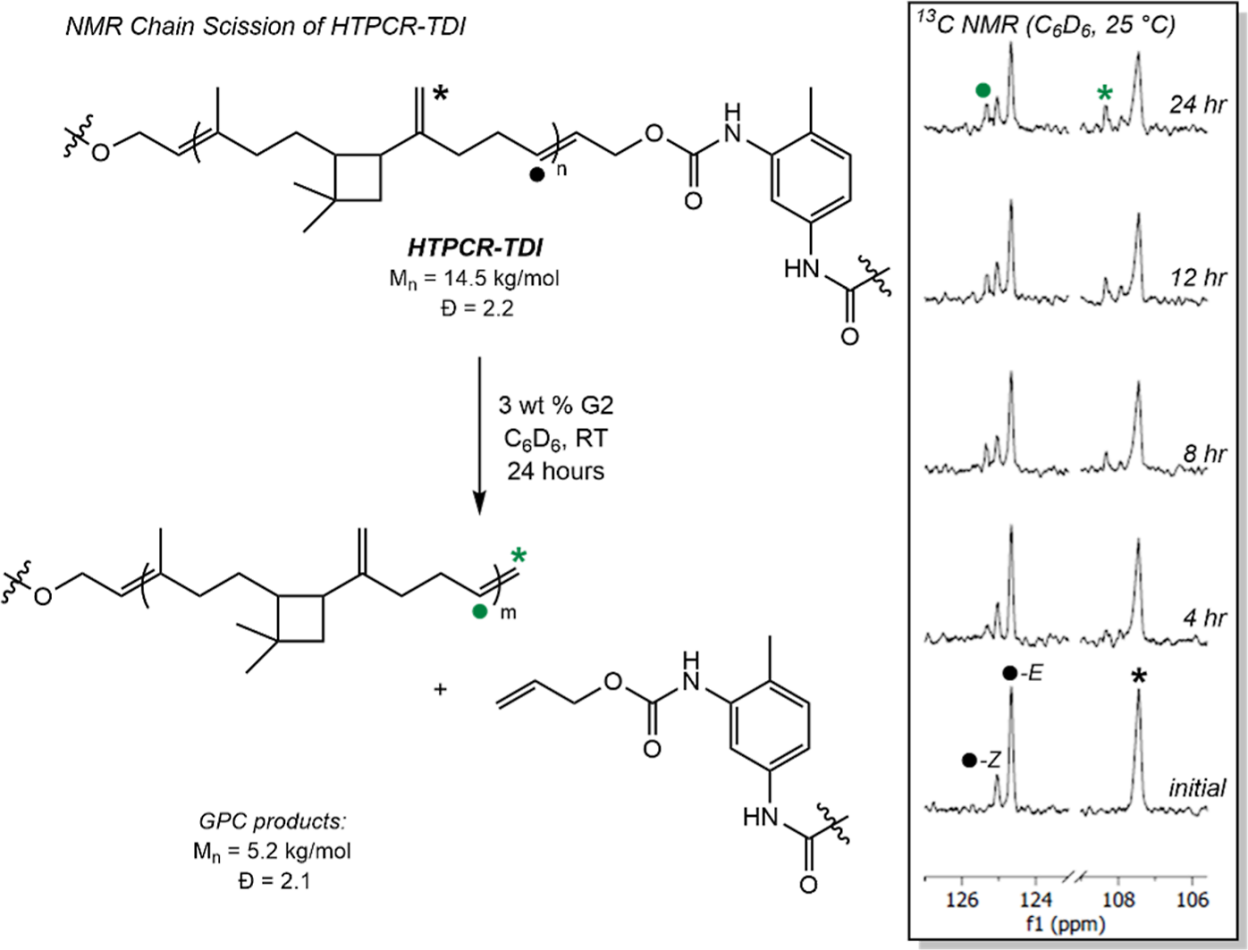
Chain scission of HTPCR-TDI monitored by ^13^C NMR (C_6_D_6_, 25 °C). Inset: identification and assignment of terminal vinyl groups arising from intramolecular ADMET. Full assignment is given in Figure S21B of the Supporting Information.

Similar phenomena were observed from the in situ NMR monitoring of the degradative cleavage of thermoset HTPCR-polyHDI under the same conditions as the NMR-scale metathesis reaction of HTPCR-TDI. As the reaction proceeded, a soluble organic product containing a 15-carbon pattern of signals appeared in the ^13^C NMR spectrum (Figure S22A). This 15-carbon pattern is consistent with the main chain backbone of HTPCR as well as independently prepared vinyl-terminated polycaryophyllene. The same vinyl chain ends were also identified in the product mixture as the dissolution of the thermoset progressed in the metathesis degradation process, indicating that polycaryophyllene units are “clipped” off the thermoset as the reaction proceeds. The same metathesis pathway was identified for the deconstruction of a PU thermoset made from the condensation of HTPCR­(6,8) with polyHDI (Figure S25); here, the elongated methylene spacer would remain with the urethane unit upon metathesis cleavage, leaving the same vinyl-terminated polycaryophyllene product as both HTPCR-TDI and HTPCR-polyHDI.

Given the experimental evidence of vinyl-terminated polycaryophyllene production during the chain cleavage process and the lack of evidence for ring closing reactions, the chain cleavage process is hypothesized to occur through several potential cross-metathesis pathways outlined in Scheme 3. Of note is the potential for the reaction to be initiated from either the metathesis-active vinylidenes or from the free terminal vinyl groups previously characterized in the HTPCR prepolymers (vide supra). As several types of substituted olefins are present in the polycaryophyllene structure, a model that accounts for the molecular weight range of products seen in the downcycling of HTPCR(1)-based PUs is the cross-metathesis of vinylidene groups and less hindered 1,2-disubstituted main-chain olefins, consistent with previously established relative rates of sterically encumbered olefins in metathesis reactions (Scheme 3A).^70–72^ Scission at the main-chain 1,2-disubstituted olefins initiated from a vinylidene would generate a branched PU structure and liberate a terminally vinylated polymer chain, with the latter functionality now potentially active in metathesis as well. Continual truncation at 1,2-disubstituted main chain olefins until complete consumption would result in a theoretical molecular weight of 4.2 kg/mol obtained for the shortest polymeric products of HTPCR-polyHDI and of HTPCR-TDI, if only consumption at these lesser sterically hindered olefins occurred. These molecular weights are consistent with the experimental molecular weights obtained from the decomposition process, particularly in the case of HTPCR-TDI (Table 5, entries 4 and 5); however, tracking the relative signal intensity by ^13^C­{^1^H} NMR spectroscopy of the olefinic signals in the depolymerization of HTPCR-TDI indicates conversion of the trisubstituted chain end as well, suggesting that disubstituted olefin cleavage may not be the only operative process (Figure S21C).

**Scheme 3:**
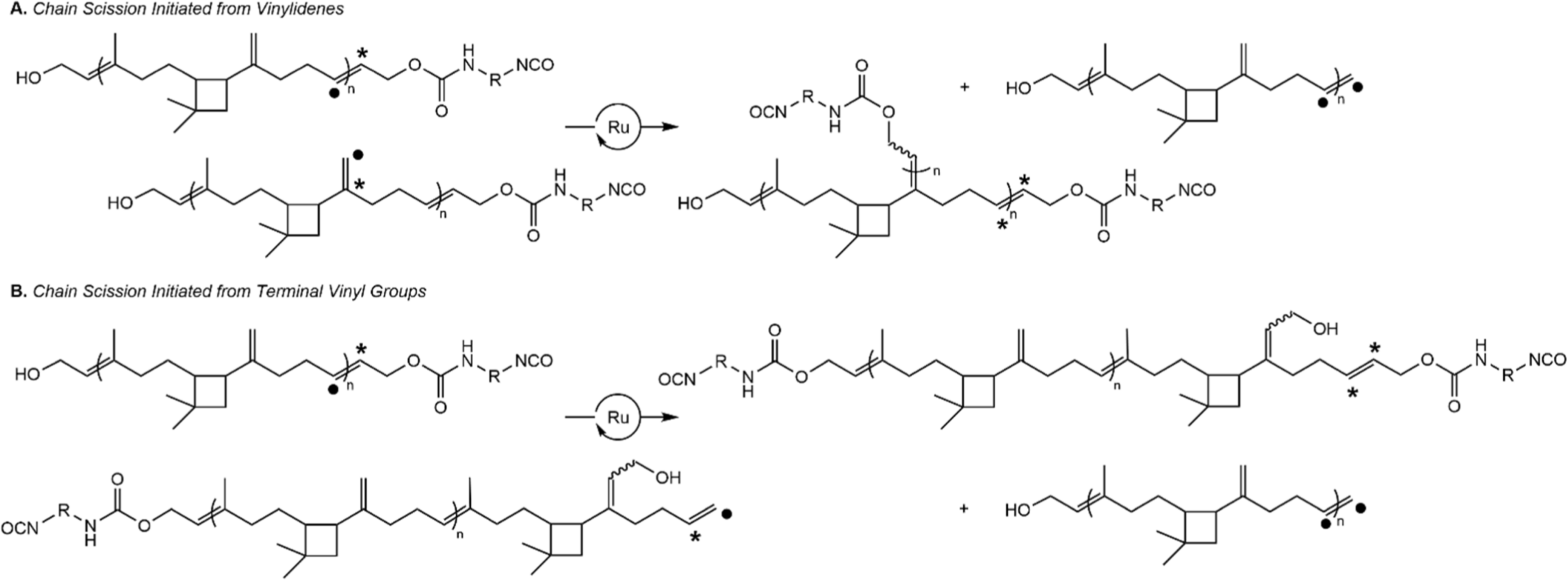
Cross-Metathesis Decomposition Routes Accounting for the Reduction in Molecular Weight of HTPCR-Based PUs upon Exposure to Grubbs Catalysts^a^

a (A) Model for chain scission initiated from pendent vinylidene groups. (B) Model for chain scission initiated by cross-metathesis from residual terminal vinyl groups.

Similar molecular weights could also be obtained if chain scission was initiated from the small percentage of terminally vinylated HTPCR(1) (Scheme 3B). Here, engagement of vinyl-terminated functionalities with 1,2-disubstituted main-chain olefins would result in degenerate molecular weight short chain products to that of vinylidene-initiated deconstruction. Further, the scission processes of Scheme 3, if conducted intramolecularly, could also produce macrocyclic products (Scheme 4). These macrocycles would still contain vinylidene functionalities that can break open the macrocyclic rings upon further metathesis; however, the presence of these macrocyclic intermediates may account for the fact that the molecular weight of degradation products obtained in Table 6 tends to exceed that of the polyol used in the PU, and that residual insoluble material is isolated in the decomposition of the HTPCR-based thermosets. Regardless of the olefin type that initiates metathesis degradation, successful deconstruction of PU thermoplastics and thermosets in this manner demonstrates that HTPCR is privileged in its inclusion of metathesis-active handles as a polyol.

**Scheme 4:**
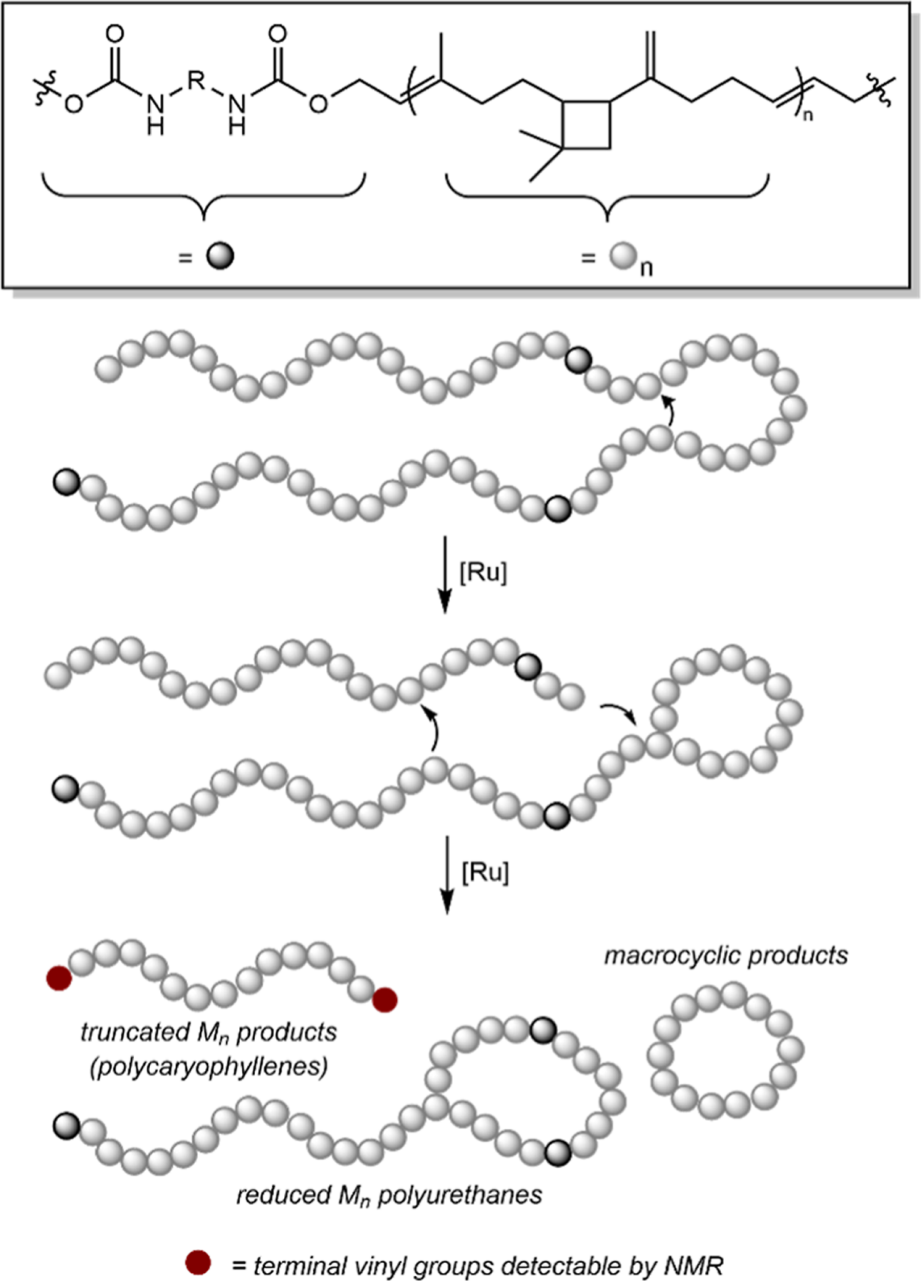
Intramolecular Chain Scission Pathways of HTPCR(1)-Based PUs Accounting for the Higher Statistical Likelihood of Producing Reduced Molecular Weight Polycaryophyllenes as Well as Potential Macrocyclic Products

To differentiate the significance of the vinylidene group as the metathesis-active initiator over that of the small content of chain end vinyl groups from HTPCR(1) synthesis, PU analogues with decreased vinylidene content were synthesized and subsequently deconstructed. HTPB-polyHDI, originating from HTPB containing approximately 15% 1,2-butadiene incorporation, was subjected to 1 wt % G2 at room temperature for 48 h. Despite containing more main chain olefins than HTPCR, only 50% of the HTPB-polyHDI thermoset material solubilized, presumably due to the decreased vinyl group content available to function as initiators (Scheme 5). As a control, full elimination of olefins is possible through the synthesis of polythioether-based thermosets, as demonstrated by Grau and co-workers;^54^ here, a thermoset lacking vinylidenes was prepared by the hydrothiolation of polycaryophyllene with trimethylolpropane tris­(3-mercaptopropionate) (PCR-trithiol, see Supporting Information for synthetic details).^54^ Attempted deconstruction of PCR-trithiol under the same conditions resulted in 95% recovery of material.

**Scheme 5:**
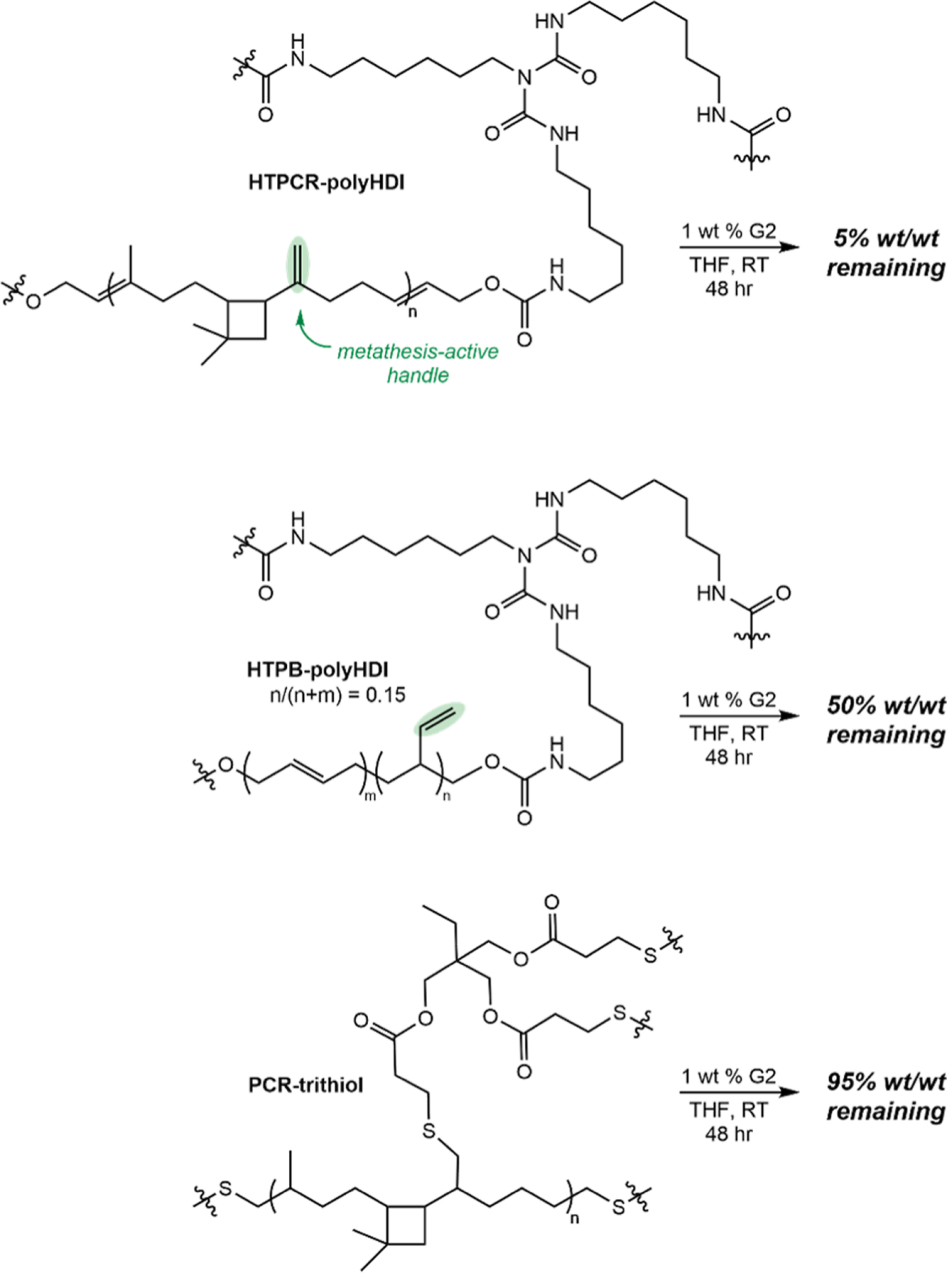
Elimination of Available In-Chain Metathesis-Active Handles Leads to Decreased Consumption of Thermoset Material in Metathesis Degradation

The short chain, vinyl-terminated polycaryophyllene produced has established precedent as a competent plasticizer in rubbers^55^ and as an elastomer in polythioethers and vulcanized thermosets.^54^ Likewise, the vinylated carbamates have proven utility as additives for radiation curing in related PU syntheses,^73,74^ demonstrating the continued value of the products obtained from HTPCR-based PU deconstruction. The utility of the products obtained from this particular downcycling process was demonstrated by cross-linking of the recovered material from the metathesis degradation of HTPCR-polyHDI thermoset. Separation of the soluble material from the decomposition mixture and purification by flash column chromatography recovered a principally polycaryophyllene thermoplastic PU (*M*_n_ = 4.9 kg/mol, *Đ* = 4.0) that was characterized by ^1^H and ^13^C NMR spectroscopy as well as by IR spectroscopy (Scheme 6 and Figure S27). The presence of remaining vinylidenes in the recovered material allowed for cross-linking of the thermoplastic back to a thermoset through thiol–ene click chemistry. Photoinitiated hydrothiolation of the recovered thermoplastic with excess trithiol trimethylolpropane tris­(3-mercaptopropionate) produced a thin, flexible film of thermoset material identified as HTPCR-polyHDI-trithiol (Scheme 6). Although unable to be cast into dogbones amenable to tensile testing without fragmenting, this material was further characterized by IR, DSC, and TGA. IR spectroscopy verified the vinylidene units as sites for cross-linking through the disappearance of the signals at ∼1630 cm^–1^, corresponding to their conversion to thioethers in the hydrothiolation process (Scheme 6 and Figure S28A). Confirmation of thermoset behavior was established through the material’s insolubility in organic solvents and lack of melting events observed by DSC. Notably, the material is characterized by its relatively high glass transition temperature of −11.4 °C when compared to the other polyHDI-cross-linked PUs examined in this study. Regardless, the material exhibited similar thermal stability to that of the pure PU as determined by TGA, with 5% decomposition occurring at 271 °C under inert atmosphere (Figure S28C). The unique composition of these formally polyurethane–polythioethers is of emerging interest as new classes of coating materials with high glass transition temperatures and/or self-healing properties,^75–78^ such that their recovery presents a potential avenue for the end-of-life fate for HTPCR-based PUs. Notably, this cross-linking process should also be possible on thermoplastic HTPCR-based PUs; the full investigation and application of these new compositions is reserved for future disclosures.

**Scheme 6:**
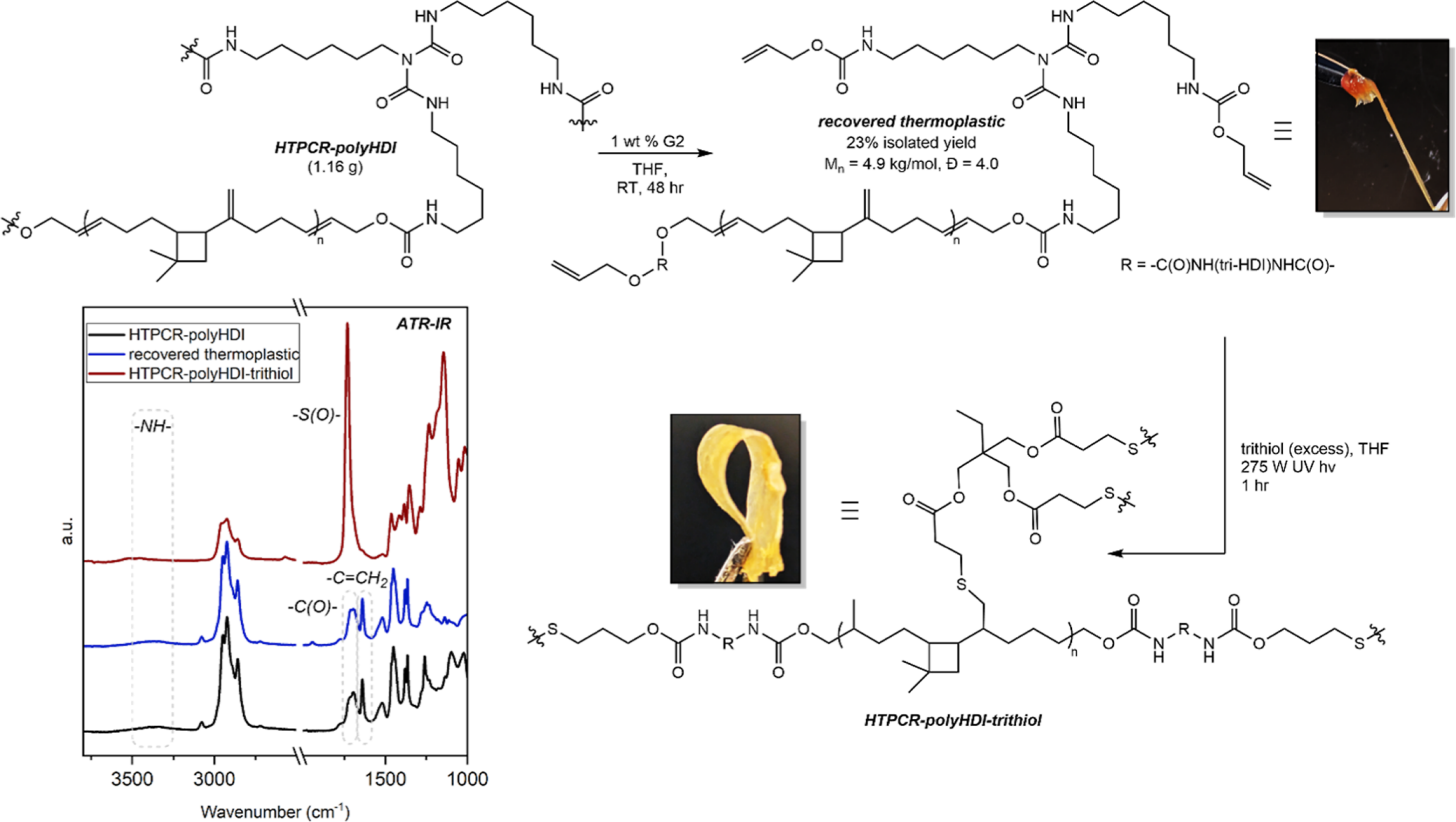
Crosslinking of Thermoplastic Material Obtained from the Decomposition of HTPCR-PolyHDI^a^

a Inset: truncated, annotated ATR-IR spectra of starting HTPCR-polyHDI (black), recovered thermoplastic PU (blue), and HTPCR-polyHDI-trithiol (red). Full IR spectra are given in Figure S28A.

## Conclusions

In this work, hydroxy termination of polycaryophyllene afforded polyols that were readily incorporated into PU thermoplastics and thermosets. These materials exhibit high thermal stability along with rheological properties that were modulated to obtain adhesive materials or elastomers. The vinylidene functionality preserved in the PU structure also provides an avenue for catalytic chain scission of the PUs by metathesis under ambient temperature and pressure conditions, and in the absence of additives. This chain scission process not only serves as a distinct mechanism from established hydrogenation protocols for PUs, but also highlights a principle for the design of polymers with the inherent ability to be chemically deconstructed. Given the breadth of the heteroatomic polymer space, future work will extend HTPCR-based polyols to a broader range of composite materials in order to assess the potential applicability of these metathesis-active handles as chain cleavage initiators and sites for material repurposing.

## Supplementary Material



## Notes

General considerations, synthetic procedures, full IR, NMR, GPC, DSC, DMA, and TGA spectra and procedures, and other characterization data (PDF)
