# Supplementary material for: Synthesis, Properties, and Metathesis Activity of Polyurethane Thermoplastics and Thermosets from a Renewable Polysesquiterpene Diol
Source: Macromolecules. 2025 Aug 1;58(15):8235–48. doi: 10.1021/acs.macromol.4c02436 (PMC12356080; doi:10.1021/acs.macromol.4c02436)
Supplement: Supplementary file 1 [file ma4c02436_si_001.pdf]

Supporting Information

Synthesis, Properties, and Metathesis Activity of Polyurethane Thermoplastics and Thermosets from a Renewable Polysesquiterpene Diol

*Carli B. Kovel,[1] Hannah Perine,[2] Paul J. Chirik,[1] and Megan Mohadjer Beromi[2]**

[1]Department of Chemistry, Princeton University, Princeton, NJ 08544, USA

[2]Department of Chemistry, United States Naval Academy, Annapolis, MD 21402, USA

mohadjer@usna.edu

Table of Contents

I. General Considerations S2

II. Synthesis and Characterization of HTPCR Polyols S6
General Procedure for Conditions Screen for Diol Synthesis S6
Gram-Scale Synthetic Procedure for HTPCR(1) S6
Representative Spectroscopic Data for Acetoxy-Terminated Polycaryophyllene S7
Representative Spectroscopic Data for HTPCR(1) S11
Gram-Scale Synthetic Procedure for HTPCR(6,8) S15
Representative Spectroscopic Data for HTPCR(6,8) S15
Determination of Hydroxylation by Trifluoroacetylation S19
Quantification of Percent Vinyl-Termination S20
Assessment of Metathesis Processes Leading to Vinyl Termination S23
Post-Synthetic Metathesis Activity of HTPCR(1) S28
DSC Data for HTPCR(1) and HTPCR(6,8) S31
TGA Data for HTPCR(1) and HTPCR(6,8) S31

III. Synthesis and Characterization of HTPCR-Based Polyurethanes S33
General Cast-Cure Procedure S33
NMR and GPC Spectroscopic Data for HTPCR-TDI Thermoplastic S35
ATR-IR Data for HTPCR-TDI and HTPCR-polyHDI S40
Gelation Test for Crosslinking of HTPCR-polyHDI Thermoset S41
TGA Data for HTPCR-TDI and HTPCR-polyHDI S42
DSC Data for HTPCR-TDI and HTPCR-polyHDI S37
DMA Data for HTPCR-TDI and HTPCR-polyHDI S44
Lap Shear Testing of HTPCR-TDI and HTPCR-polyHDI S47

Tensile Testing of HTPCR-TDI and HTPCR-polyHDI S48

IV. Metathesis Activity of Thermoplastics and Thermosets S50
General Procedure for Decomposition of Polymer Composites S50
Decomposition Catalyst Screen S50
Catalyst Loading Screen S52
GPC Data for PU Decomposition Conditions Screen S54
Determination of Decomposition Products by NMR S55
Determination of Decomposition Products by GC/MS S62
Control Experiments S63
Synthesis and NMR Decomposition of HTPCR(6,8)-based PU Thermoset S64
Synthesis of PCR-trithiol Thermoset S68
Crosslinking of Decomposition Products S69

V. References S74

I. General Considerations

All air- and moisture-sensitive manipulations were carried out using vacuum line, Schlenk and cannula techniques or in an MBraun inert atmosphere (nitrogen) dry box unless otherwise noted. All glassware was stored in a pre-heated oven prior to use. The solvents used for air- and moisture-sensitive manipulations were dried and deoxygenated using literature procedures.[1] β-caryophyllene, Grubbs catalysts, cis-2-butene-1,4-diol, cis-1,4-diacetoxy-2-butene, hydroxy-terminated polybutadiene (HTPB, M_n ~1200), TDI, polyHDI, dibutyltin dilaurate (DBTDL), trimethylolpropane tris(3-mercaptopropionate), and 2,2-Dimethoxy-2-phenylacetophenone (DMPA) were purchased in reagent grade from Fischer, Aldrich or Acros and stored under inert atmosphere. Chain terminators cis-2-butene-1,4-diol and cis-1,4-diacetoxy-2-butene were dried over calcium hydride and deoxygenated prior to use. Polycaryophyllene was prepared according to literature procedures with a M_n of 30.050 kg/mol and a Đ of 2.192.[2] HTPB-polyHDI was prepared according to literature procedures, but was cured at room temperature instead of elevated temperature. The spectroscopic signatures obtained for room temperature curing were identical to those previously reported.[3]

^{1}H and ^{13}C NMR spectra were recorded on JEOL JNM-ECZS Series 400 or Bruker Avance III 500 spectrometers operating at 399.80/100.54 MHz or 500.46/125.86 MHz, respectively. All ^{1}H and ^{13}C NMR chemical shifts are reported in ppm relative to $SiMe_4$ using the ^{1}H (benzene-d_6: 7.16 ppm) and ^{13}C (benzene-d_6: 128 ppm) chemical shifts of the solvent as a standard. ^{1}H NMR data are reported as follows: chemical shift, multiplicity (s = singlet, d = doublet, t = triplet, q = quartet, p = pentet, br = broad, m = multiplet, app = apparent, obsc = obscured), coupling constants (Hz), integration. ^{13}C NMR data for diamagnetic compounds are reported by their chemical shift. ATR-IR spectra were recorded on a ThermoScientific Nicolet iSIO spectrometer equipped with a SMART iTR ATR-IR sampling accessory and OMNIC software.

TGA data were recorded on a TA Instruments TGA5500 equipped with an IR furnace capable of ballistic heating or a TA Instruments Q500 using 3-7 mg of material in a platinum crucible. For

inert atmosphere experiments, the crucible was held at a temperature of 30 °C in an atmosphere of nitrogen in the furnace, after which the material was heated to 800 °C at a rate of 10 °C/min under nitrogen flow. Data were analyzed using Universal Analysis software.

DSC data were recorded on a TA Instruments Q20 scanning calorimeter equipped with a RCS40 cooling system using 3-6 mg of material in crimp-sealed TA standard aluminum pans. The pans were referenced to a duplicate empty aluminum pan. For determination of melting and crystallization temperatures, the temperature was cycled in the following sequence: isothermal 25 °C for 5 minutes, ramp 10 °C/min to 250 °C, isothermal 250 °C for 5 minutes, ramp 10 °C/min to -40 °C, isothermal -40 °C for 5 minutes, ramp 10 °C/min to 250 °C, isothermal 250 °C for 5 minutes, ramp 10 °C/min to -40 °C, isothermal -40 °C for 5 minutes, ramp 10 °C/min to 25 °C. For determination of glass transition temperatures of polyols, the temperature was cycled in the following sequence: Isothermal 25.00 °C for 5 minutes, ramp 5 °C/min to -100.00 °C, isothermal -100.00 °C for 5 min, ramp 5 °C/min to 25.00 °C, isothermal 25.00 °C for 5 min, ramp 5 °C/min to -100.00 °C, isothermal -100.00 °C for 5 min, ramp 5 °C/min to 25.00 °C. Data were analyzed using Universal Analysis software.

Viscosity and density data were recorded on an SVM 3001 Stabinger viscometer (Anton Paar) with temperature controlling capabilities. 2 mL samples were loaded directly into the instrument, and the cell was kept at 25.000 °C during measurements.

DMA data were recorded on a TA Instruments Discovery HR-2 rheometer equipped with a thin film-fiber accessory in tension mode. Temperature sweeps were conducted from -25 to 50 °C at a ramp rate of 3 °C/min with a minimum axial force of 1 N, sensitivity of 0.1 N, axial displacement of 25 μm and frequency of 1.0 Hz. Runs were analyzed using TRIOS software.

Tensile and lap shear testing was conducted on an ADMET MTEST Quattro Materials Testing System with an Interface SM-250 250 lbf force transducer. Materials were suspended for testing using either dual pneumatic grips or lap shear testing pins. The position was adjusted at a rate of 1.000 inch per minute until the load was 4.800 lb, or the sample load dropped 10% (indicating

breakage). The sampling rate was 127 samples/second. Dogbones and adhered aluminum plates were prepared in duplicate and the elongation at break, load at break, Young's Moduli, or shear strength averaged.

GPC data were recorded on an Agilent Technologies 2000 Series gel-permeation chromatograph (GPC) equipped with two Polymer Laboratories PL gel (5 μm 500 Å 300 x 7.5 mm) columns and a Wyatt Optilab refractive index detector using THF as the eluent. The column was kept at 30 °C and a flow rate of 1.0 mL/min was utilized. The GPC was calibrated with polystyrene standards obtained from PSS-Polymer.

GC/MS data were recorded on an Agilent 6890N gas chromatograph system equipped with a Zebron ZB-5MS phase column (l = 30 m, i.d. = 0.25 mm, df = 0.25 μm) and an Agilent 7683B Series injector/autosampler. Mass data were obtained with an Agilent 5975 inert mass selective detector. The GC method conditions utilized were: equilibrate at 30 °C for 0.5 min, ramp to 50 °C at 5°C/min, ramp to 300 °C at 20°C/min, hold at 300 °C for 5 min.

MALDI-TOF data were recorded on a Shimadzu Biotech AXIMA Confidence instrument equipped with a variable repetition rate 50 Hz nitrogen laser operating in positive ion, linear mode. Analyte solutions of 8 mg HTPCR in 2 mL of uninhibited THF, cation solutions of 2 mg of sodium trifluoroacetate in 1 mL of uninhibited THF, and matrix solutions of 20 mg of 2,5-dihydroxybenzoic acid in 1 mL of uninhibited THF were mixed in a 1:1:4, 1:1:1, and 4:4:1 volume ratio on the target plate and dried at ambient temperature and pressure prior to analysis.

II. Synthesis and Characterization of HTPCR Polyols

General Procedure for Conditions Screen for Diol Synthesis. In a typical experiment, β-caryophyllene (1.00 g, 4.893 mmol) was added to a 50 mL round-bottom flask along with a PTFE-coated stir bar. To the round bottom was added G2 dissolved in toluene (1.63 mL). The mixture was allowed to stir for 5 minutes, after which the diol CTA was added dropwise with a syringe. The reaction was sealed in an inert atmosphere and then allowed to stir at 50 °C for 24 hours. The catalyst was deactivated through addition of ethyl vinyl ether (1 mL) and the polymer was recovered by dropwise addition into methanol. The methanol was decanted, and the resulting polymer was dried under vacuum for a minimum of 24 hours to remove residual solvent. The polymer was then analyzed by NMR and GPC with data reported in Tables 1 and 2 of the main text.

Gram-Scale Synthetic Procedure for HTPCR(1).

Synthesis of acetoxy-terminated polycaryophyllene: The preparation and deprotection procedure was adapted from that of previously reported hydroxy-terminated polyisoprene.[4] In an inert atmosphere, a 500 mL round-bottom flask was charged with a PTFE-coated magnetic stir bar and β-caryophyllene (90.0 g, 0.440 mol). G2 (0.748 g, 0.880 mmol) was dissolved in toluene (220 mL) and the solution was added to the round-bottomed flask. While the mixture stirred, cis-1,4-diacetoxy-2-butene was added dropwise (9.36 mL, 58.7 mmol). The round-bottom flask was sealed with a rubber septum and heated to 50 °C for 24 hours. Conversion was determined by ^{1}H NMR spectroscopy of a reaction aliquot. The reaction was terminated by the addition of ethylvinyl ether (6.9 mL). The mixture was stirred for 20 minutes, after which the polymer was precipitated by the addition into anhydrous methanol. The supernatant was removed, and the polymer was washed with anhydrous methanol two additional times. The resulting polymer was dried under vacuum for an excess of 24 hours and 74.0 g was isolated in 92.5% yield.

Representative spectroscopic data for acetoxy-terminated polycaryophyllene. ^{1}H NMR (500 MHz, benzene-d_6, 25 °C): δ 5.55 (m, 3H); 5.44 (m, 3H); 5.32 (m, 9H); 5.27 (m, 4H); 4.92 (m, 34H); 4.63 (d, 4H); 2.48 (m, 16H); 2.29 (m, 35H); 2.15 (m, 39H); 2.03 (m, 47H); 1.91 (m, 9H); 1.82 (m, 16H); 1.76 (app. s, 15H); 1.72 (app. s, 11H); 1.65-1.49 (m, 105 H). Acetate end groups: CH_2: 4.63, CH_3: obs. 2.30. The ^{1}H NMR spectrum is given in Figure S1A. ^{13}C NMR (126 MHz, benzene-d_6, 25 °C): δ 170.14; 152.70; 152.64; 152.57; 152.42; 142.66; 141.99; 135.91; 135.56; 135.50; 130.54; 130.04; 128.35; 125.06; 124.68; 124.61; 119.80; 119.27; 65.07; 61.22; 60.88; 49.73; 49.39; 48.89; 48.75; 46.86; 42.21; 42.09; 40.03; 39.92; 38.63; 38.28; 35.55; 35.24; 35.16; 34.30; 33.92; 33.83; 31.62; 31.46; 31.14; 30.28; 30.10; 29.54; 27.19; 27.11; 26.40; 23.73; 23.53; 22.62; 22.52; 20.64; 16.37; 16.25; 15.88. Acetate end groups: *C*=O: 170.84, *C*H_2: 65.07 *C*H_3: 20.26. The ^{13}C{^{1}H} NMR spectrum is given in Figure S1B. ^{1}H-^{1}H COSY, ^{1}H-^{13}C HSQC, ^{1}H-^{13}C HMBC, and ^{13}C APT NMR experiments were used to establish the identity of the chain end -CH_2-OAc resonance; the annotated spectra are given in Figure S1C-F. ATR-IR: (cm^{-1}, assignment): 1741 cm^{-1}, -C=O, 1640 cm^{-1}, -C=C- and -C=CH_2. The ATR-IR spectrum is given in Figure S1G.

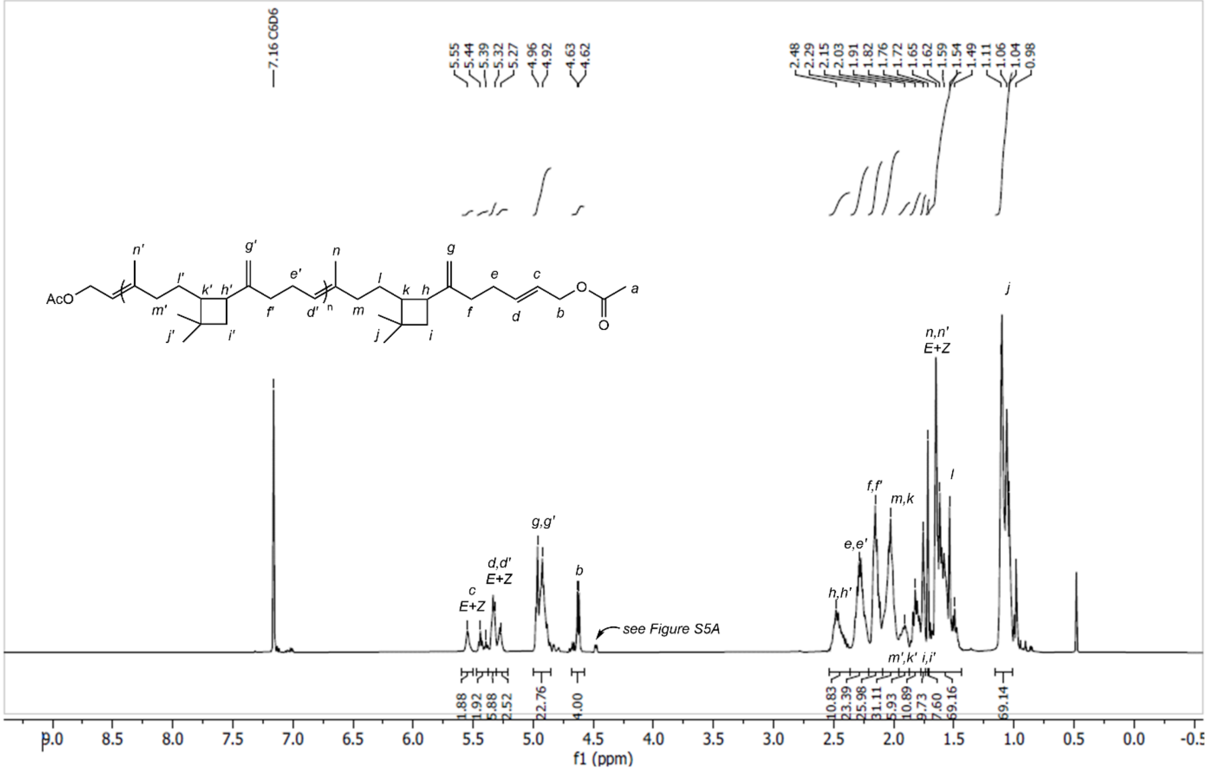

Figure S1A. ^{1}H NMR (benzene-d_6, 25 °C) spectrum of acetoxy-terminated polycaryophyllene.

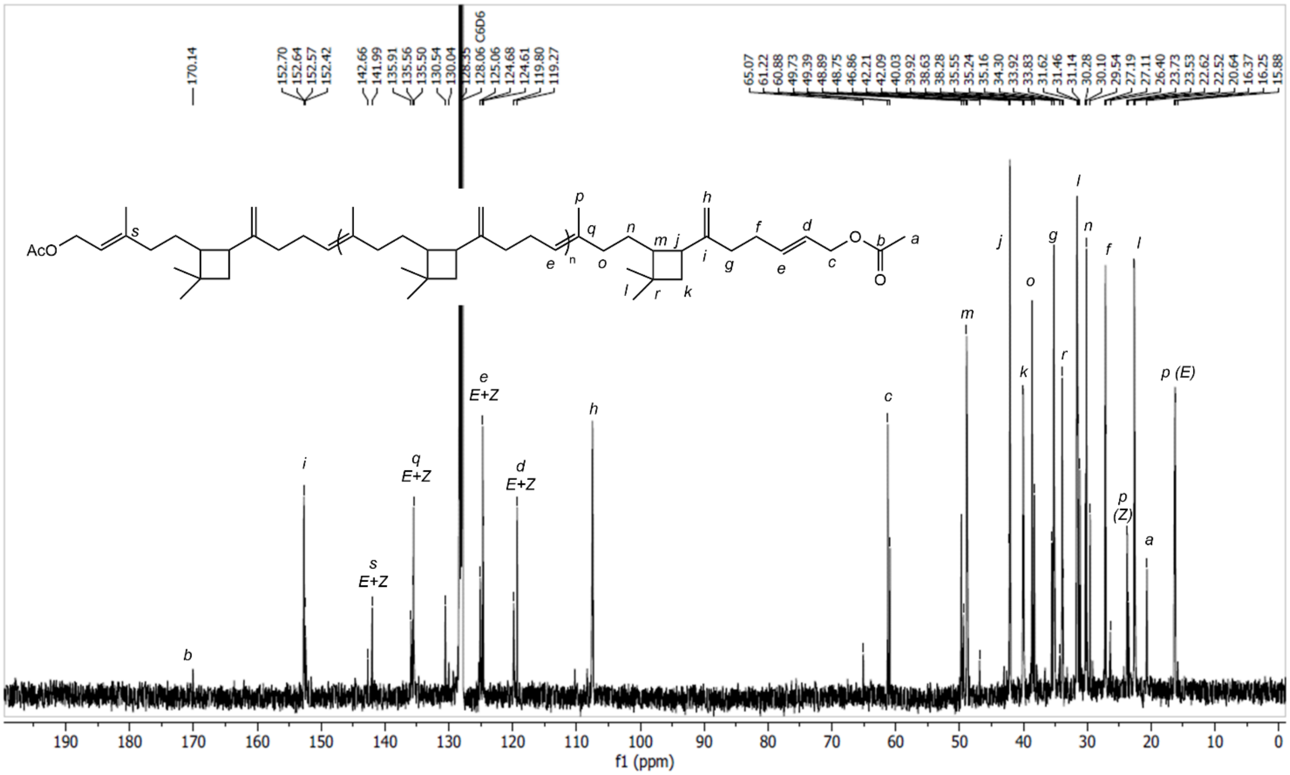

Figure S1B: $^{13}C\{^1H\}$ NMR (benzene-d_6, 25 °C) spectrum of acetoxy-terminated polycaryophyllene.

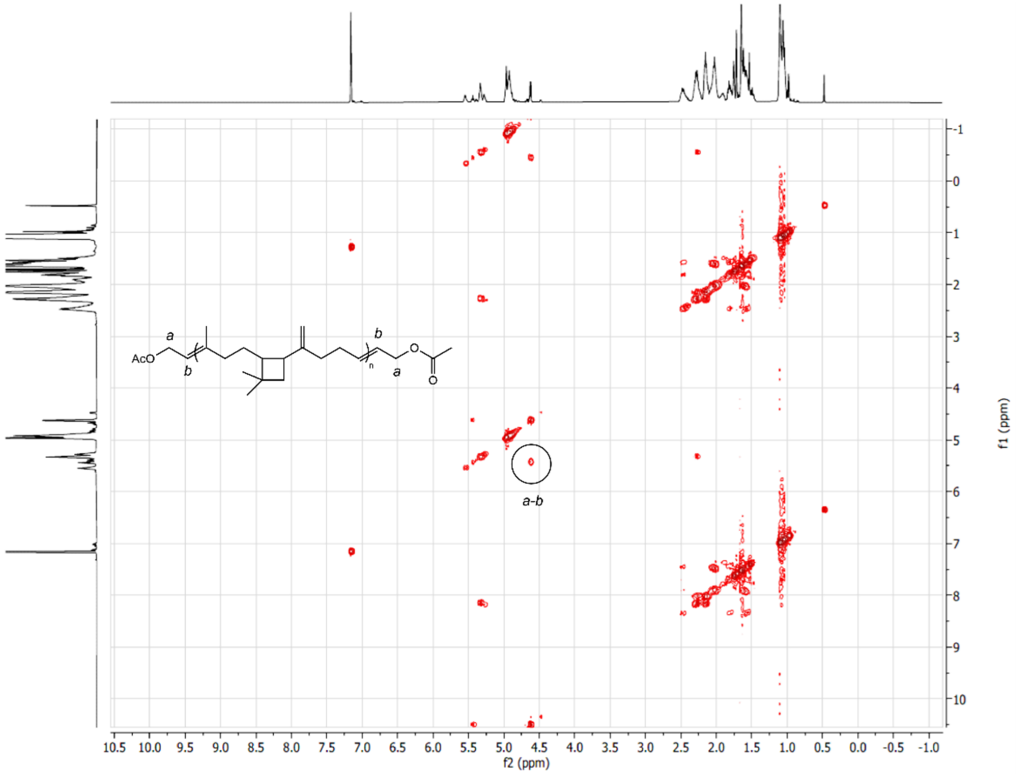

Figure S1C: Annotated ^{1}H-^{1}H COSY NMR (benzene-d_6, 25 °C) spectrum of acetoxy-terminated polycaryophyllene.

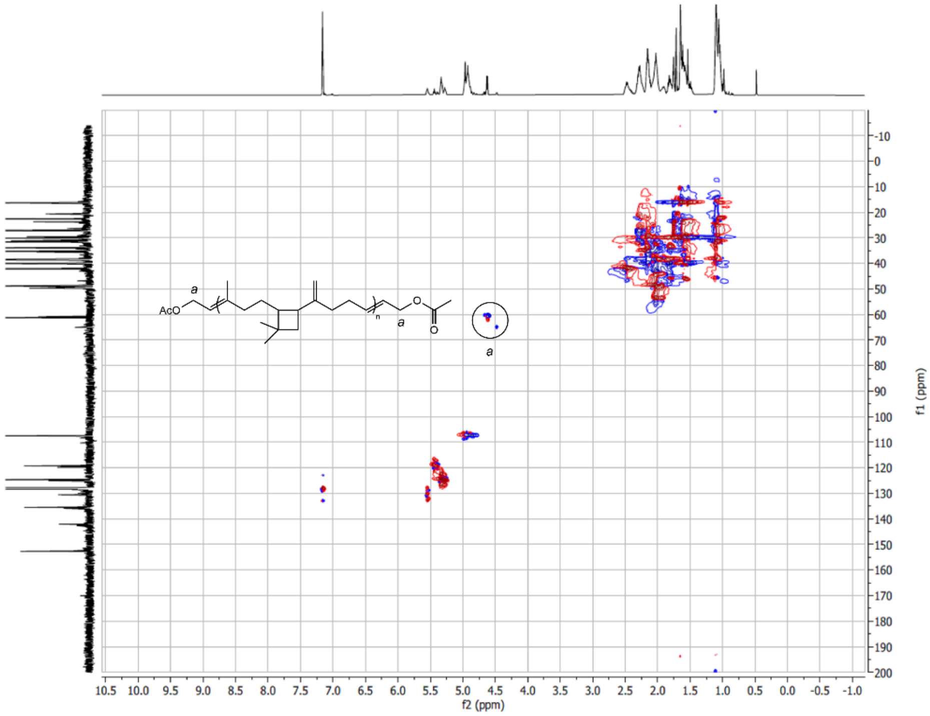

Figure S1D: Annotated HSQC NMR (benzene-d_6, 25 °C) spectrum of acetoxy-terminated polycaryophyllene.

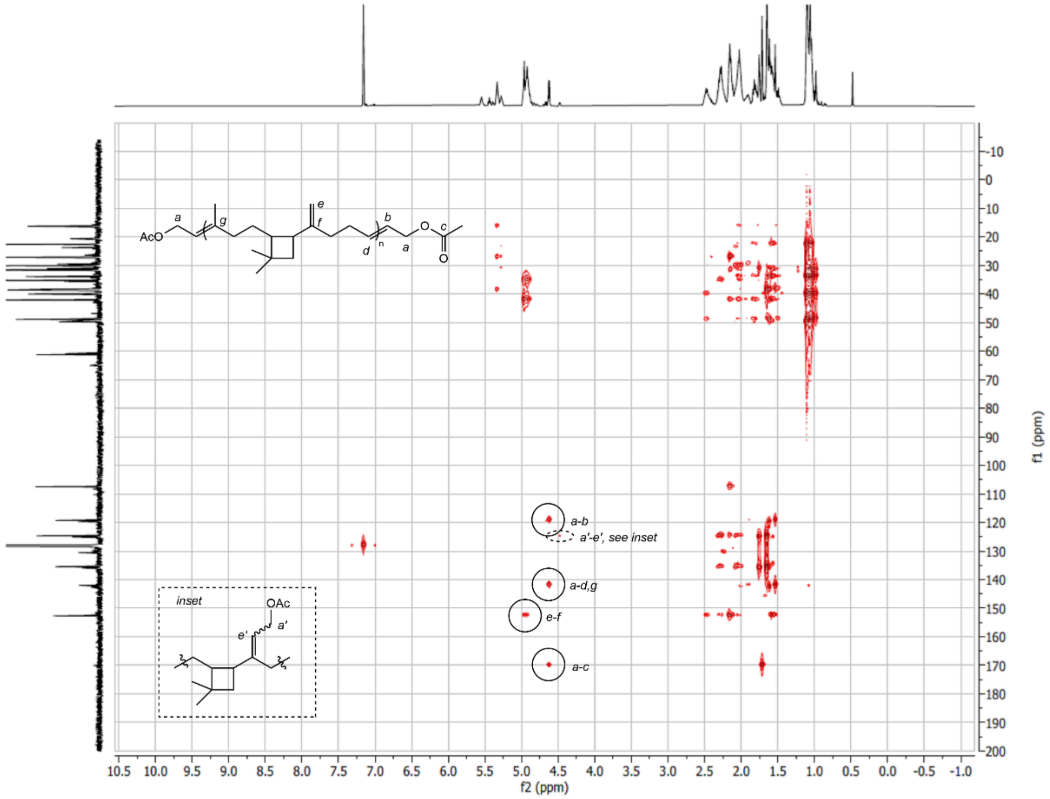

Figure S1E: Annotated HMBC NMR (benzene-d_6, 25 °C) spectrum of acetoxy-terminated polycaryophyllene.

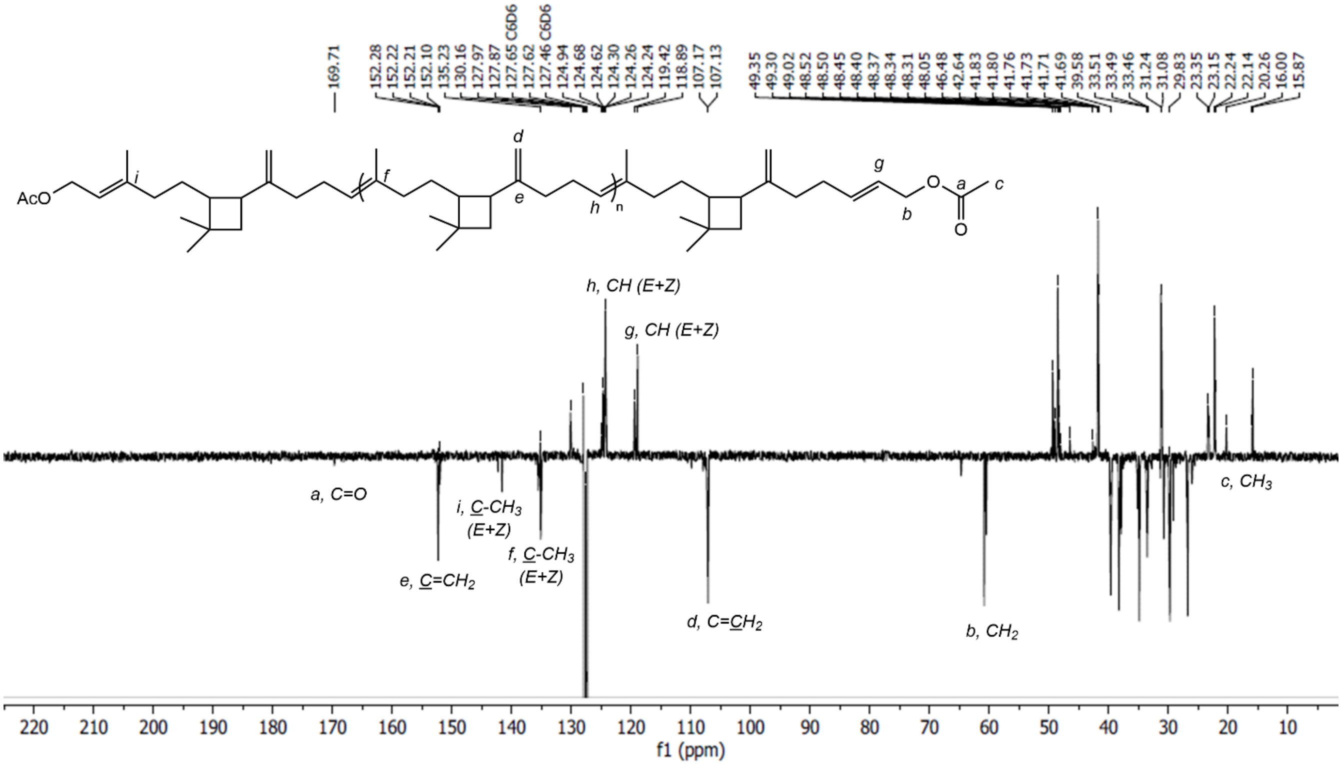

Figure S1F: Annotated APT NMR (benzene-d_6, 25 °C) spectrum of acetoxy-terminated polycaryophyllene.

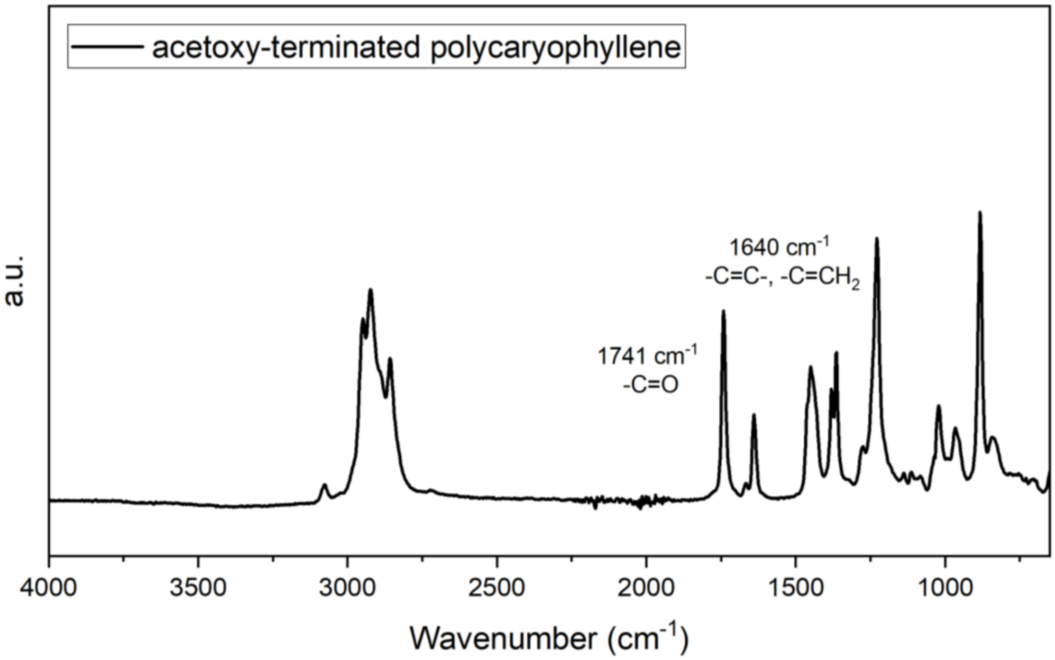

Figure S1G. ATR-IR spectrum of acetoxy-terminated polycaryophyllene.

Deprotection of acetate end groups: Acetoxy-terminated polycaryophyllene (74.0 g) was dissolved in THF in a round-bottom flask cooled to 0 °C. A 25 wt% solution of sodium methoxide solution in methanol (10.0 mL) was prepared and added dropwise to the reaction mixture. The reaction was stirred for 24 hours at 23 °C. The reaction mixture was precipitated into 2.0 L acidic methanol (20.0 mL of concentrated HCl in 1.98 L of anhydrous methanol) by dropwise addition using a dropping funnel. The supernatant was decanted, and the resulting polymer was dried for a minimum of 24 hours under vacuum, after which 41.7 g of the product was isolated in a 57.2% yield.

Representative Spectroscopic Data for HTPCR(1) (M_n = 4.820 kg/mol, Đ = 1.5). ^{1}H NMR (400 MHz, benzene-d_6, 25 °C): δ 5.54 ppm (app s, 2H); 5.39 (t, 2H); 5.32 (m, 20H); 5.27 (m, 4 H); 4.00 (m, 4H); 2.45 (m, 28H); 2.26 (m, 58H); 2.14 (m, 64H); 2.01 (m, 84H); 1.81 (m, 29H); 1.75 (s, 6H); 1.64 (m, 144H), 1.49 (s, 8H); 1.10 (m, 88H); 1.05 (72H); 1.02 (app s, 10H). The ^{1}H NMR spectrum is given in Figure S2A. ^{1}H-^{1}H COSY NMR was used to establish the identity of the chain end -

CH_2-OH resonance at 4.00 ppm; the ^{1}H-^{1}H COSY NMR spectrum is given in Figure S2B. ^{13}C{^{1}H} NMR (101 MHz, benzene-d$_6$, 25 °C): δ 152.67; 152.60; 152.39; 138.44; 135.88; 135.47; 125.23; 124.65; 124.09, 107.44; 59.39; 48.81; 42.07; 40.18; 39.99; 38.60; 35.52; 35.21; 33.91; 31.63; 31.52; 31.28; 30.24; 30.07; 29.87; 27.10; 23.87; 23.76; 22.62; 16.35; 16.13. The ^{13}C{^{1}H} NMR spectrum is given in Figure S2C. ATR-IR: (cm^{-1}, assignment): 3319 cm^{-1}, -OH, 1640 cm^{-1}, -C=C- and -C=CH_2. The ATR-IR spectrum is given in Figure S2D. Molecular weight and dispersity were determined by GPC; the chromatogram is given in Figure S2E. Quantitation of OH functional groups was conducted by MALDI-TOF analysis; the mass spectrum is given in Figure S2F.

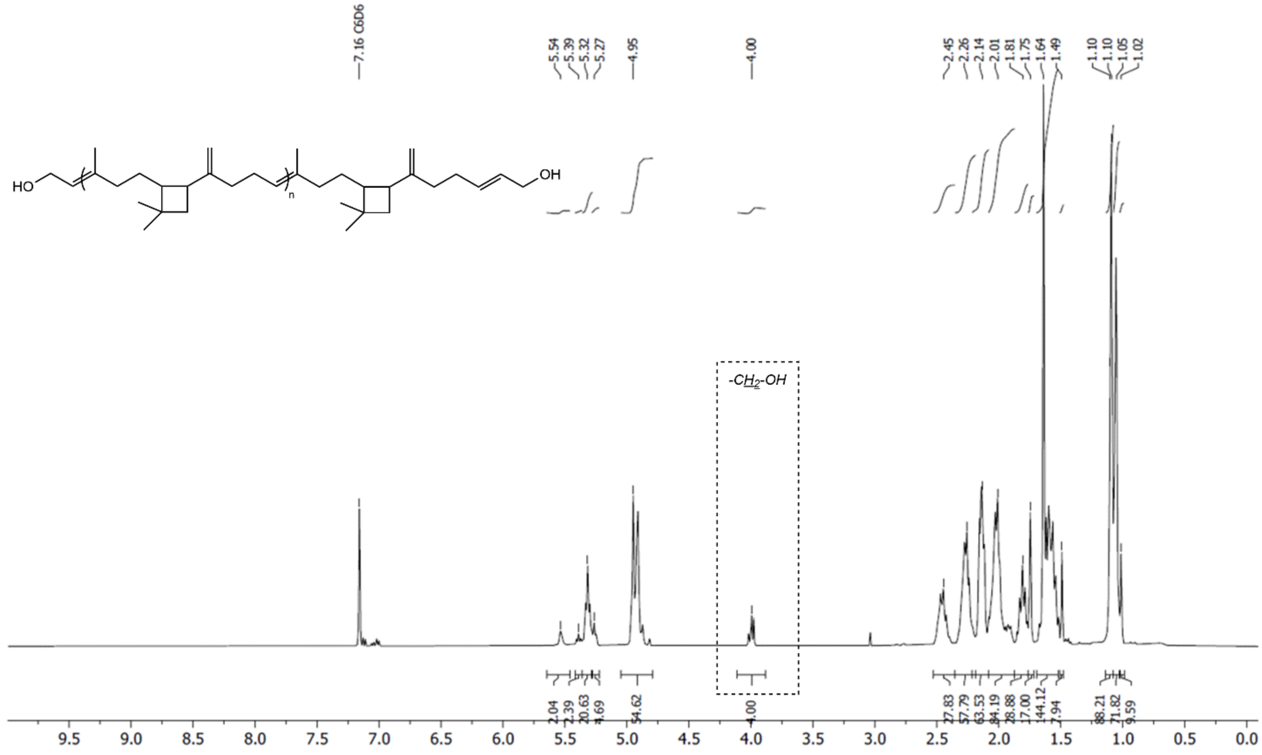

Figure S2A. ^{1}H NMR (benzene-d_6, 25 °C) spectrum of HTPCR(1) (M_n = 4.820 kg/mol, Đ = 1.5).

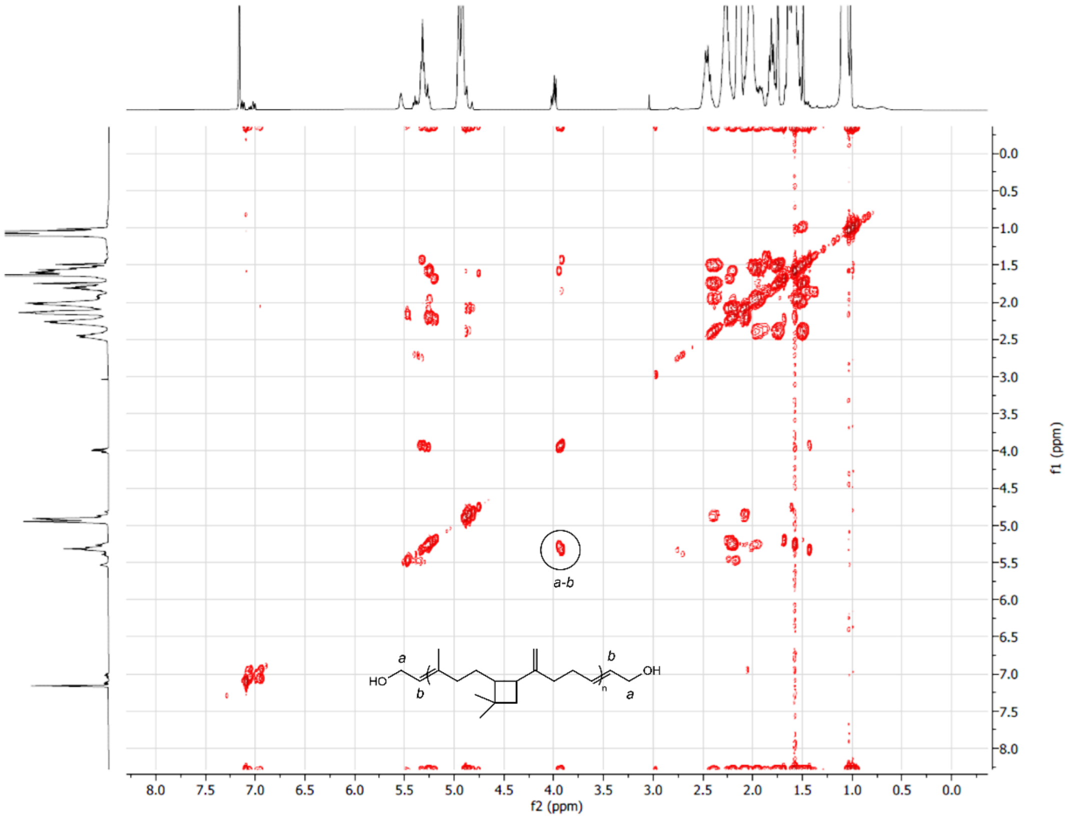

Figure S2B. Annotated ^{1}H-^{1}H COSY NMR (benzene-d_6, 25 °C) spectrum of HTPCR(1) (M_n = 4.820 kg/mol, Đ = 1.5).

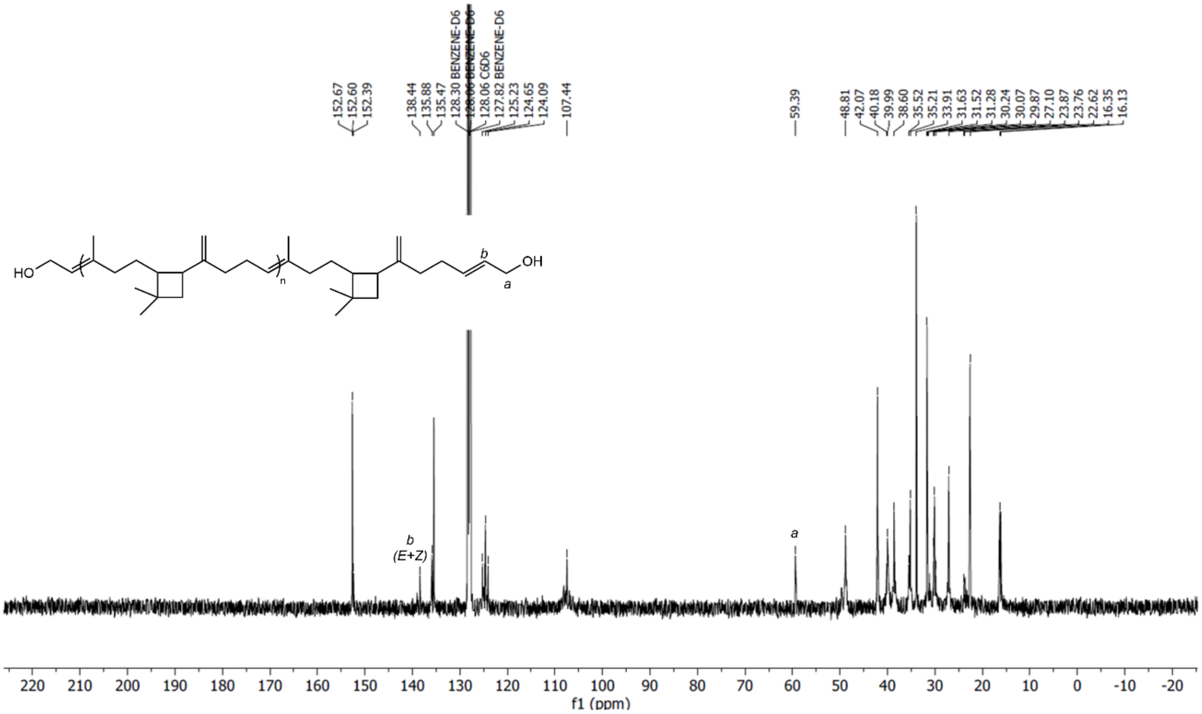

Figure S2C: $^{13}C\{^{1}H\}$ NMR (benzene-d_6, 25 °C) spectrum of HTPCR(1) (M_n = 4.820 kg/mol, Đ = 1.5).

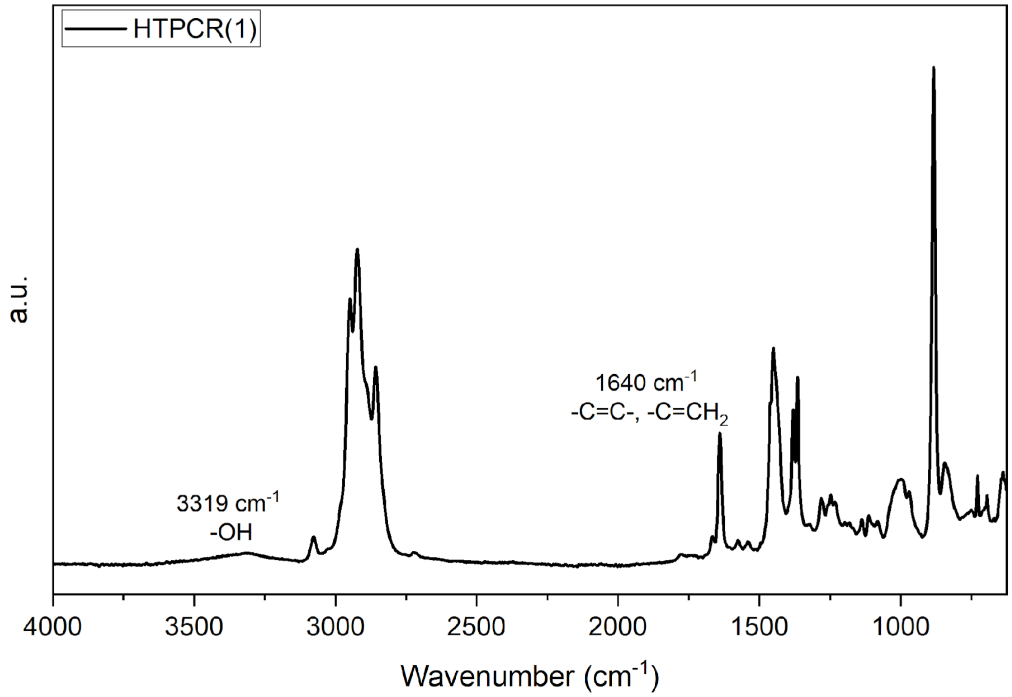

Figure S2D: Annotated ATR-IR spectrum of HTPCR(1) (M_n = 4.820 kg/mol, Đ = 1.5).

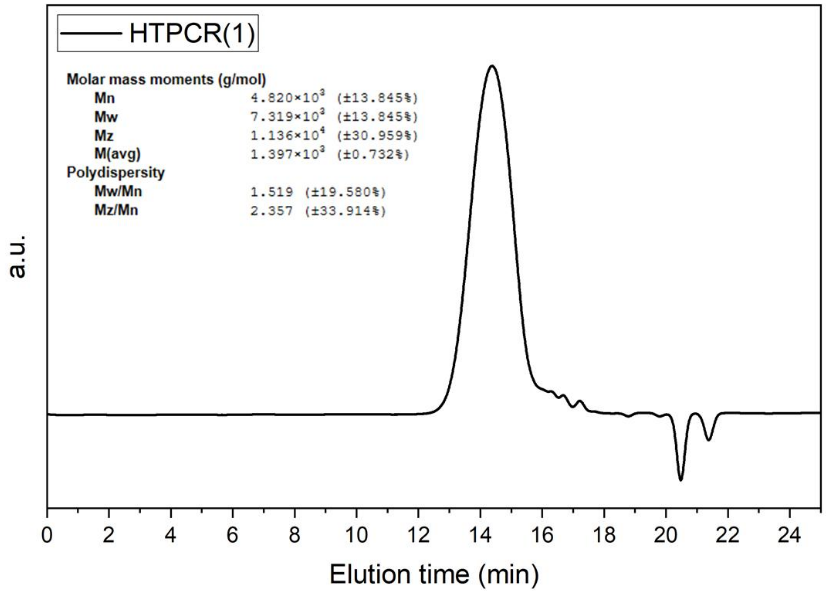

Figure S2E: GPC Chromatogram of HTPCR(1) (M_n = 4.820 kg/mol, Đ = 1.5).

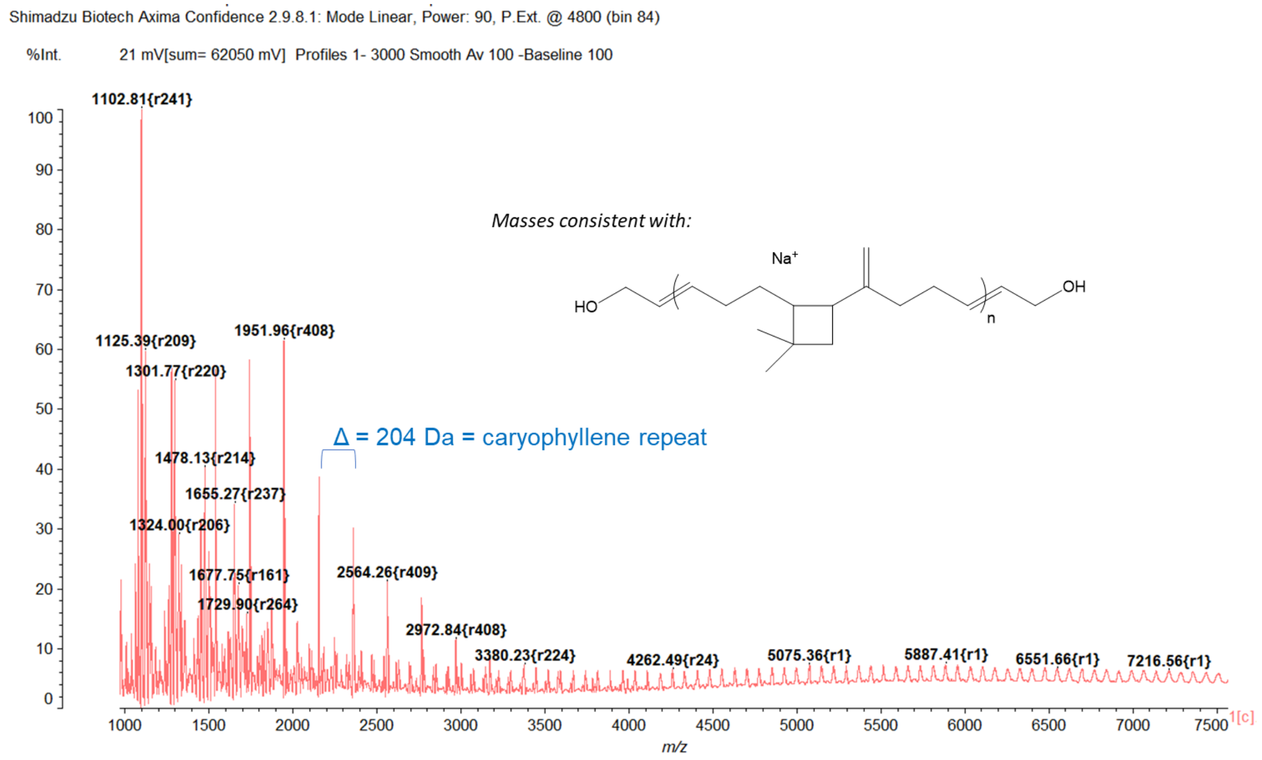

Figure S2F: Annotated MALDI-TOF spectrum of HTPCR(1) (M_n = 4.820 kg/mol, Đ = 1.5).

Gram-scale Synthetic Procedure for HTPCR(6,8): in a 100 mL round bottom flask, cis-7-hexadec-6-ene-1,16-diol (1.00 g, 3.58 mmol) and β-caryophyllene (10.0 g, 48.9 mmol) were dissolved in 15.3 mL of toluene. A solution of G2 (40 mg, 0.0471 mmol) dissolved in 1 mL of toluene was added to the round bottom flask and stirred for 12 hours at 50 °C. Full conversion was determined by ^{1}H NMR spectroscopy. The reaction was cooled to room temperature and then quenched with the addition of ethyl vinyl ether (2.00 mL). The mixture was stirred for 20 minutes, after which the product was precipitated by addition of 200 mL of methanol. The supernatant was decanted, and the resulting polymer was washed two more times with methanol. The polymer was dried for a minimum of 24 hours. The product was isolated (7.40 g) in 67.2% yield.

Representative Spectroscopic Data for HTPCR(6,8) (M_n = 2.770 kg/mol, Đ = 1.2). ^{1}H NMR (400 MHz, benzene-d_6, 25 °C): δ 5.53 (m, 2H); 5.32 (m, 12H); 4.91 (m, 24H); 3.36 (app t, 4H); 2.48 (m, 14H); 2.26 (m, 25H); 2.14 (m, 25H); 2.01 (m, 42H); 1.81 (t, 14H); 1.75 (s, 8H); 1.64 (s,

36H); 1.59 (m, 34H); 1.39 (m, 16H); 1.25 (m, 12H); 1.10 (s, 40H); 1.05 (s, 30H); 1.00 (s, 3H); 0.92 (s, 3H). The ^{1}H NMR spectrum is given in Figure S3A. $^{13}C\{^{1}H\}$ NMR (101 MHz, benzene-d_6, 25 °C): δ 152.67, 152.61, 152.39, 151.50, 135.89, 135.51, 135.47, 135.23, 134.68, 132.26, 130.52, 128.30, 128.06, 127.82, 125.44, 125.32, 125.18, 125.11, 125.04, 124.85, 124.66, 124.62, 107.41, 62.75, 62.72, 53.75, 51.89, 49.67, 48.83, 48.76, 46.77, 42.98, 42.16, 42.09, 40.80, 40.69, 40.32, 40.04, 39.98, 39.87, 38.71, 38.65, 38.61, 37.92, 35.91, 35.53, 35.21, 35.10, 33.93, 33.90, 33.87, 33.11, 32.86, 31.62, 31.57, 31.11, 30.42, 30.37, 30.24, 30.10, 30.07, 29.80, 29.72, 29.60, 29.10, 29.00, 28.87, 28.84, 28.48, 28.40, 27.15, 27.10, 26.37, 26.23, 26.16, 25.91, 22.61, 16.24. The $^{13}C\{^{1}H\}$ NMR spectrum is given in Figure S3B. ATR-IR: (cm^{-1}, assignment): 3315 cm^{-1}, -OH, 1640 cm^{-1}, -C=C- and -C=CH_2. The ATR-IR spectrum is given in Figure S3C. Molecular weight and dispersity were determined by GPC; the chromatogram is given in Figure S3D. Quantitation of OH functional groups was conducted by MALDI-TOF analysis; the mass spectrum is given in Figure S3E.

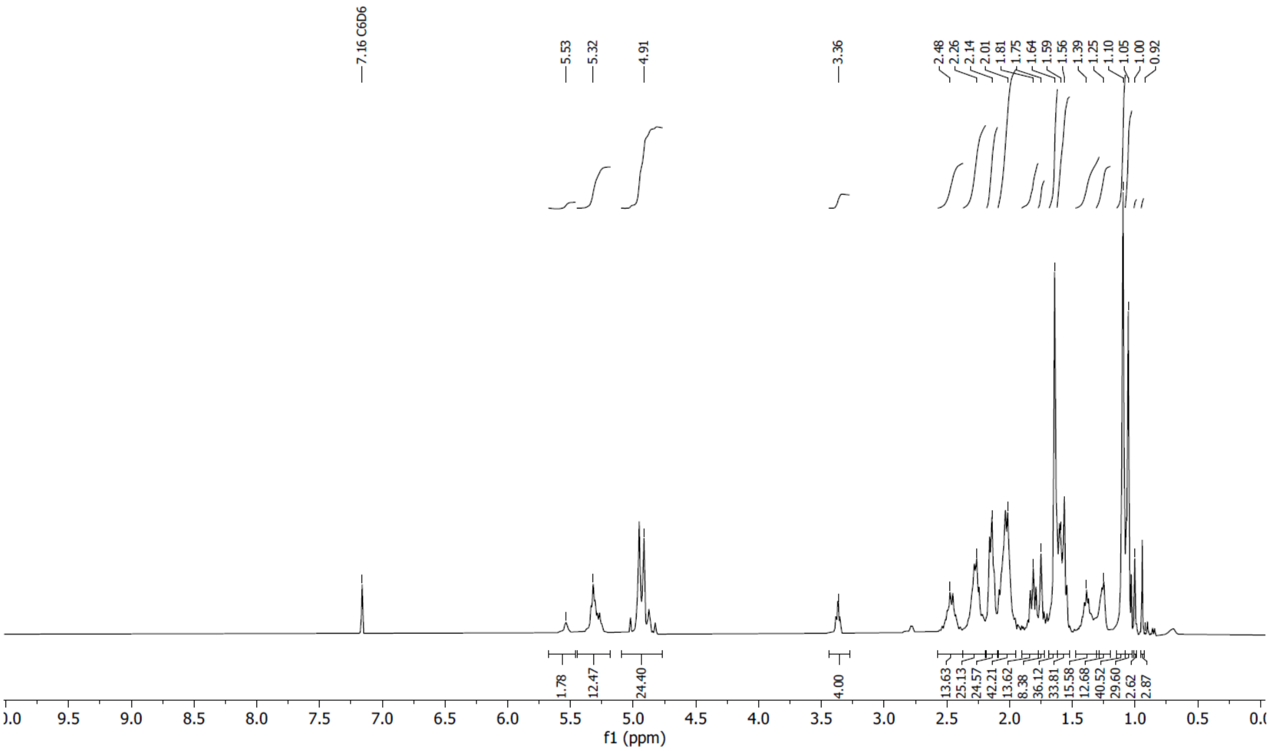

Figure S3A. ^{1}H NMR (benzene-d_6, 25 °C) spectrum of HTPCR(6,8) (M_n = 2.770 kg/mol, Đ = 1.2).

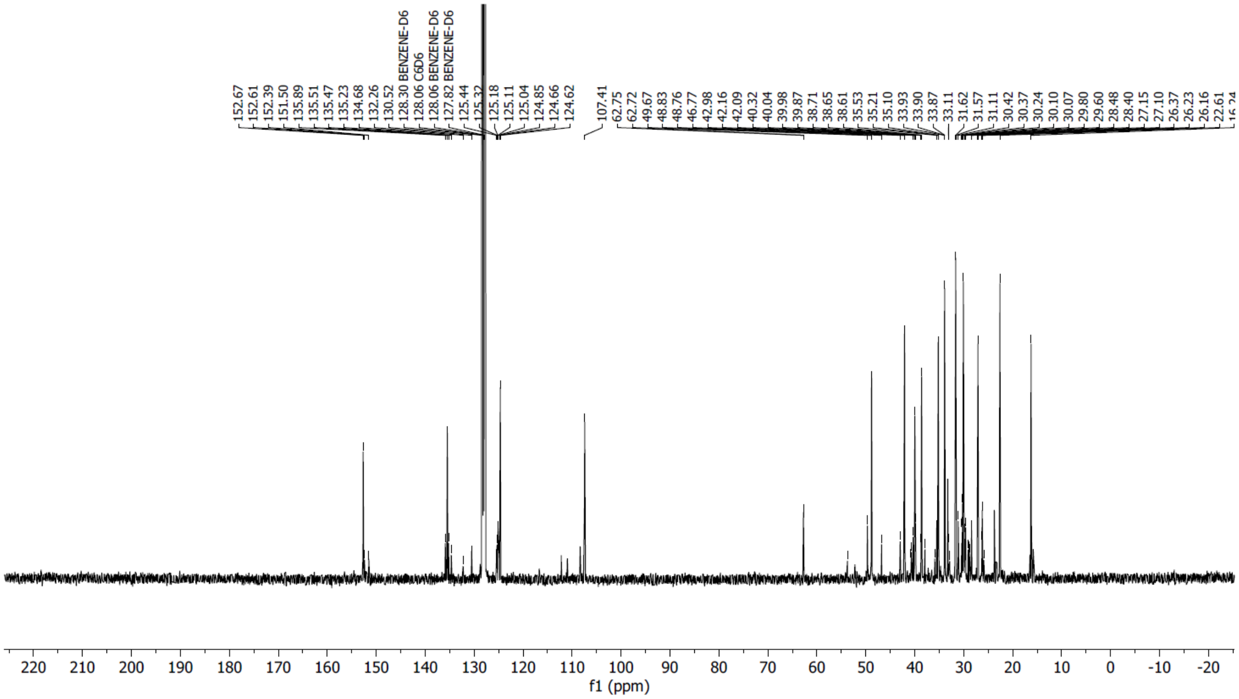

Figure S3B: $^{13}C\{^1H\}$ NMR (benzene-d_6, 25 °C) spectrum of HTPCR(6,8) (M_n = 2.770 kg/mol, Đ = 1.2).

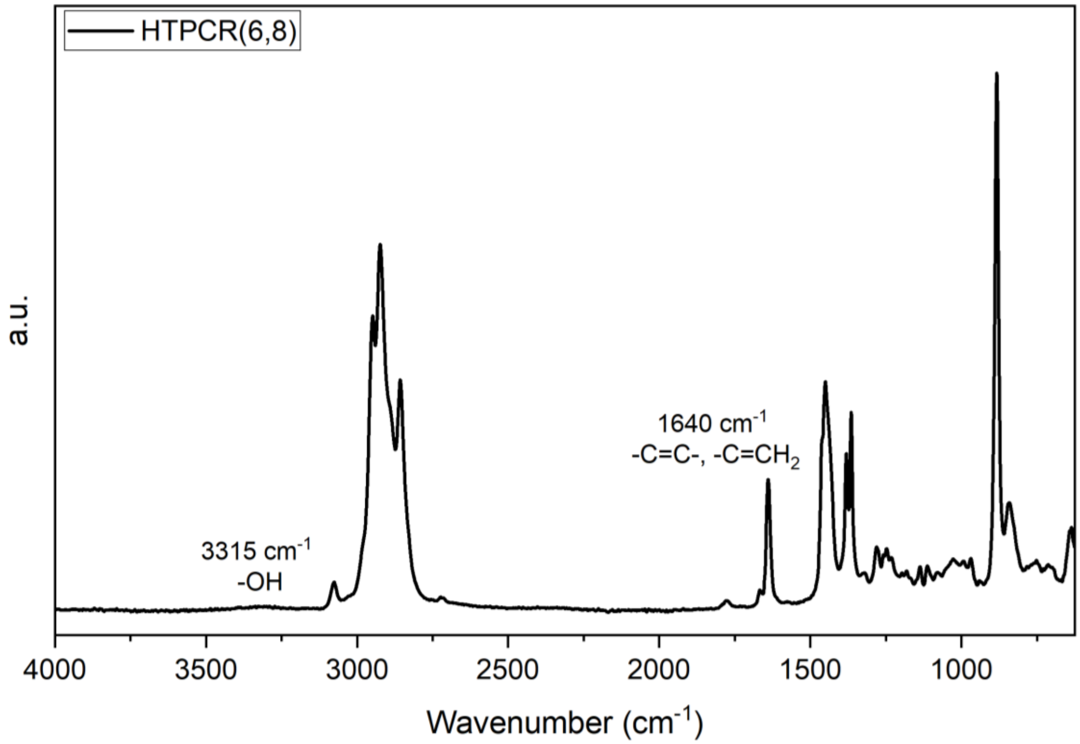

Figure S3C: Annotated ATR-IR spectrum of HTPCR(6,8) (M_n = 2.770 kg/mol, Đ = 1.2).

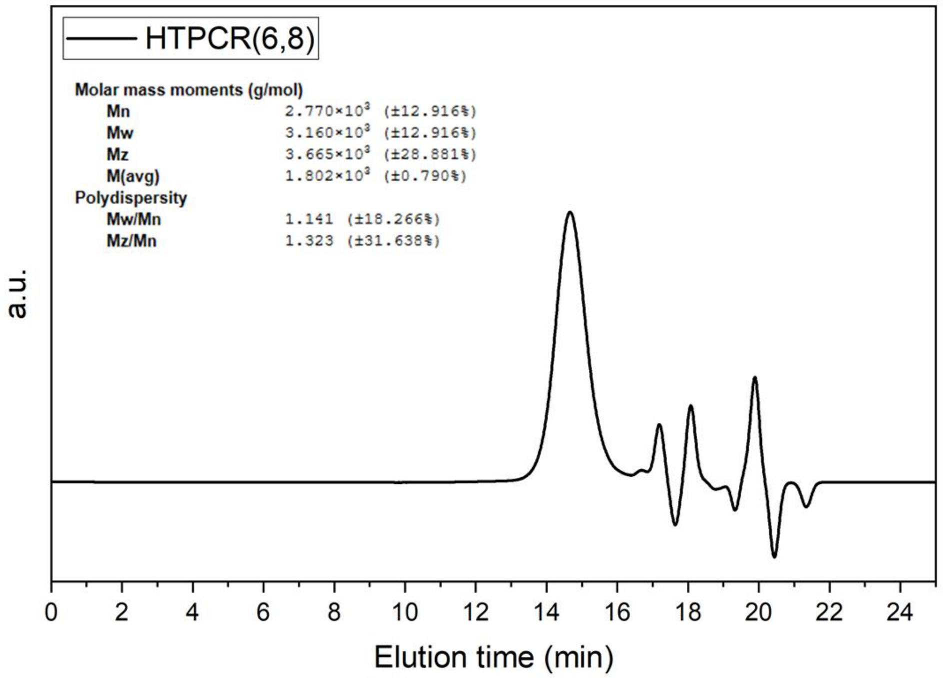

Figure S3D: GPC Chromatogram of HTPCR(6,8) (M_n = 2.770 kg/mol, Đ = 1.2).

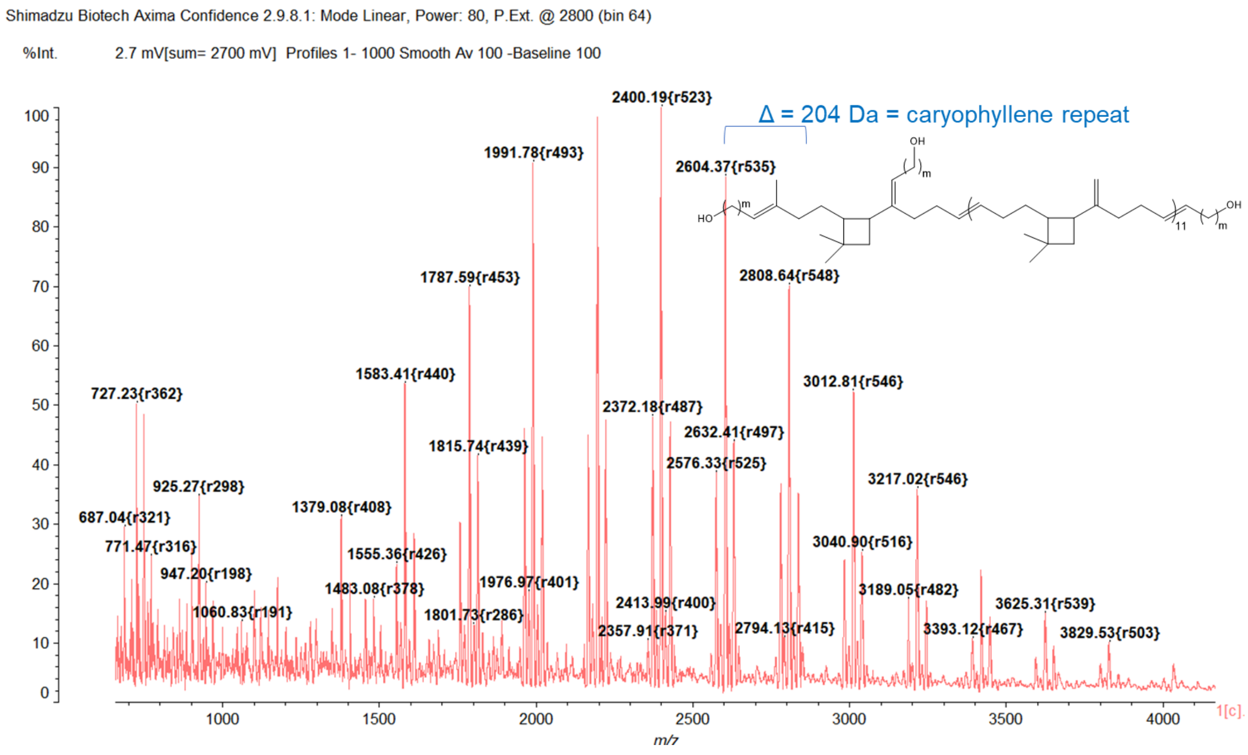

Figure S3E: Annotated MALDI-TOF spectrum of HTPCR(6,8) (M_n = 2.770 kg/mol, Đ = 1.2).

Determination of Hydroxylation by Trifluoroacetylation. In order to definitively identify the presence of telechelic hydroxy groups on the polymer products, the hydroxy groups were trifluoromethylacetylated in the following procedure. To a vial was added 50.0 mg (0.0178 mmol) of HTPCR(6,8) and a PTFE-coated stir bar. The polymer was dissolved in 1 mL of chloroform, after which 11 μL (0.0784 mmol, 4.4 equiv.) of trifluoroacetic acid anhydride was added with a syringe. The vial was capped and stirred at room temperature for 30 minutes, after which the solvent was removed under vacuum. The residue was reconstituted in 800 μL of benzene-*d*$_6$ and a ^{1}H NMR spectrum was obtained. A comparative ^{1}H NMR spectrum of HTPCR(6,8) and trifluoroacetylated HTPCR(6,8) is given in Figure S4.

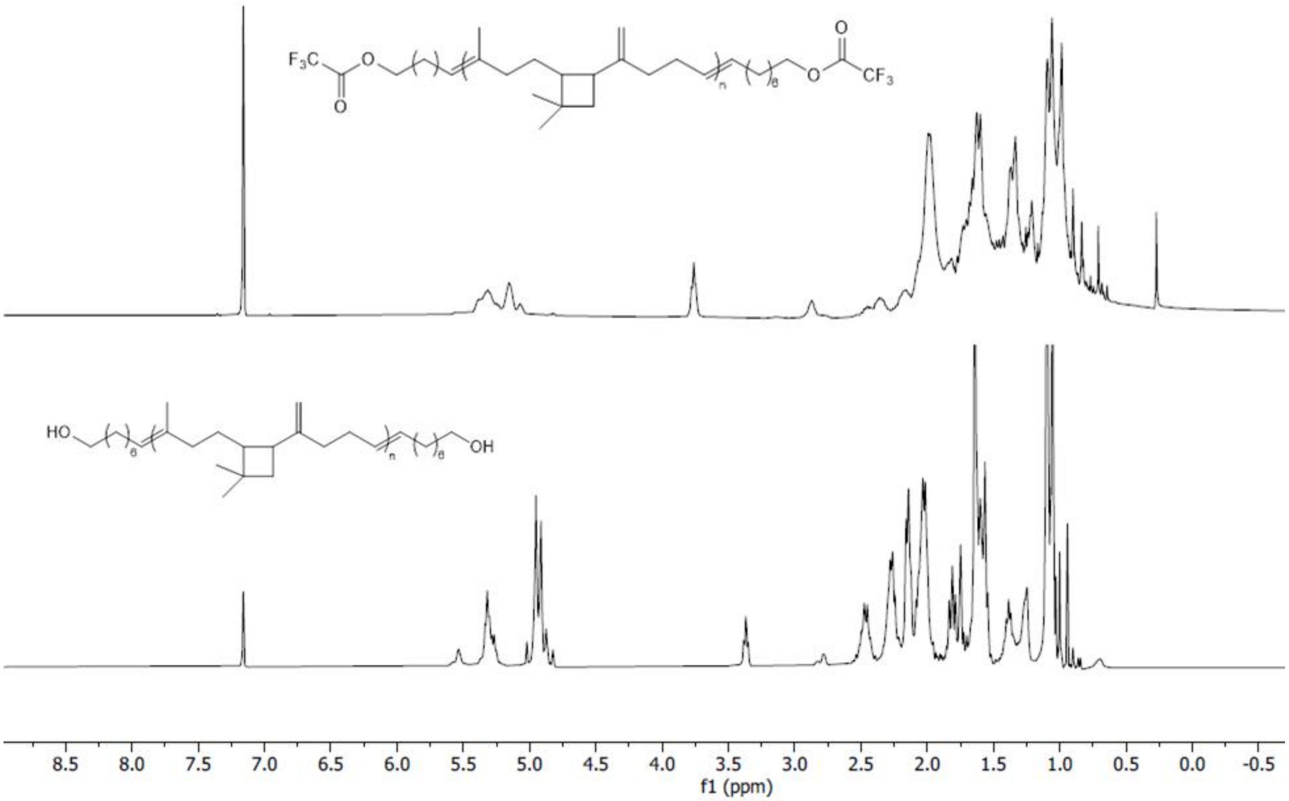

Figure S4. Stacked ^{1}H NMR (benzene-d$_6$, 25 °C) spectrum of HTPCR(6,8) (M_n = 2.770 kg/mol, Đ = 1.2) (bottom) and trifluoroacetylated HTPCR(6,8) top.

Quantification of Vinyl Termination. In order to quantify the amount of vinyl-termination present in the polyols, the chain ends of acetoxy-terminated polycaryophyllene, HTPCR(1) (synthesized from acetoxy-terminated polycaryophyllene), and HTPCR(6,8) were analyzed by quantitative ^{13}C NMR in benzene-d_6. In the case of acetoxy-terminated polycaryophyllene, ^{1}H NMR was also utilized due to the observation of a doublet at 4.48 ppm attributable to the α-carbon of the acetoxy group attached to a vinylidene. Integration of the relative amounts of the internally substituted acetoxy group against the analogous terminally-substituted chain end acetoxy groups at 4.63 ppm indicated a 5.5% incorporation of acetoxy groups at the exocyclic vinylidenes. The ^{1}H NMR spectrum is given in Figure S5A. As the analogous internal substitution could not be identified for the hydroxy-terminated variants, quantification of vinyl termination was limited to quantitative $^{13}C\{^{1}H\}$ NMR.

In the quantitative $^{13}C\{^{1}H\}$ NMR spectra, percent vinyl termination was determined by integration of the terminal vinyl peak at 108.3 and the caryophyllene repeat vinylidene peak at 107.5 ppm relative to a methyl group on the cyclobutane moiety at 22.6 ppm. Given that the methyl group should integrate in a 1:1 ratio to the vinylidene in the α,ω-terminated polymer, deviation from the ratio indicates consumption of the vinylidenes during metathesis concomitant with the appearance of terminal vinyl groups. The resulting annotated spectra for acetoxy-terminated polycaryophyllene, HTPCR(1), and HTPCR(6,8) are given in Figures S5B-D, respectively. In reporting the percent vinyl termination, the deviation from the 1:1 ratio of vinylidene to methyl group was used to mitigate the impact of the high signal-to-noise ratio of the vinyl groups on the calculated percentage values.

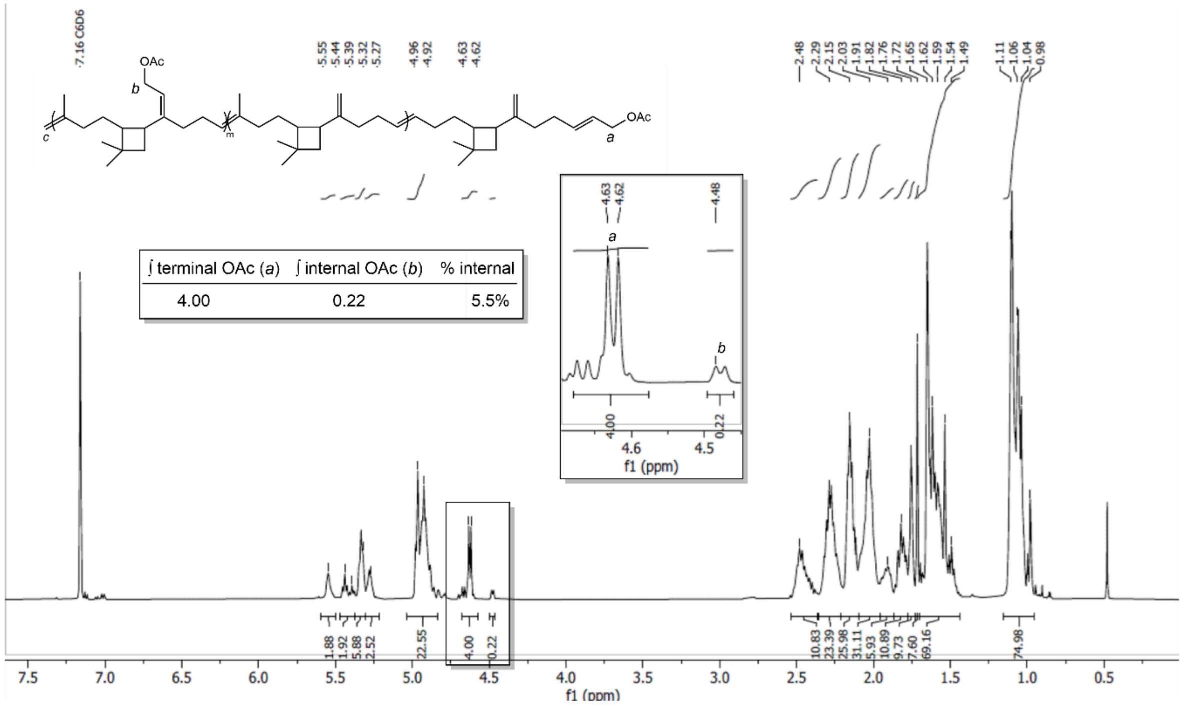

Figure S5A. Chain end analysis by ^{1}H NMR of acetoxy-terminated polycaryophyllene. Inset: signals diagnostic of terminal- or internal-acetoxy substitution utilized for quantification.

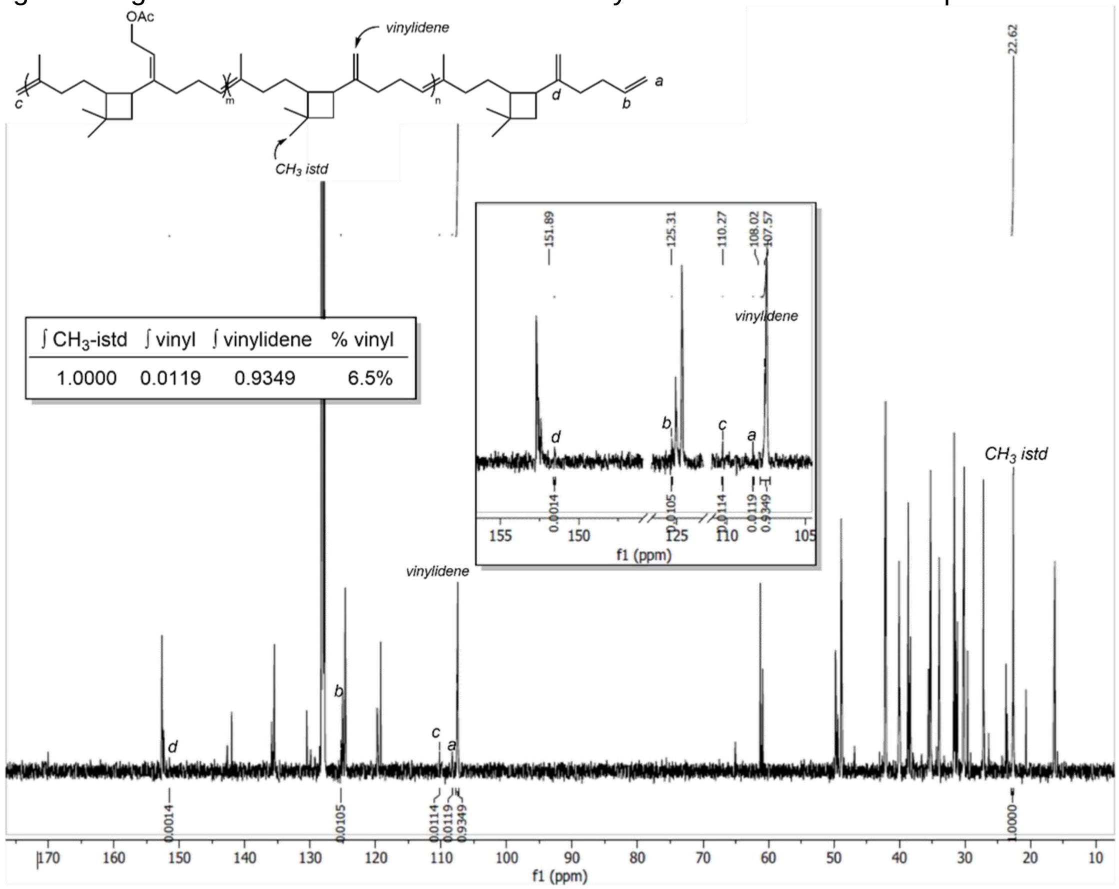

Figure S5B. Chain end analysis by quantitative $^{13}C\{^{1}H\}$ NMR of acetoxy-terminated polycaryophyllene. Inset: signals diagnostic of vinyl-termination utilized for quantification.

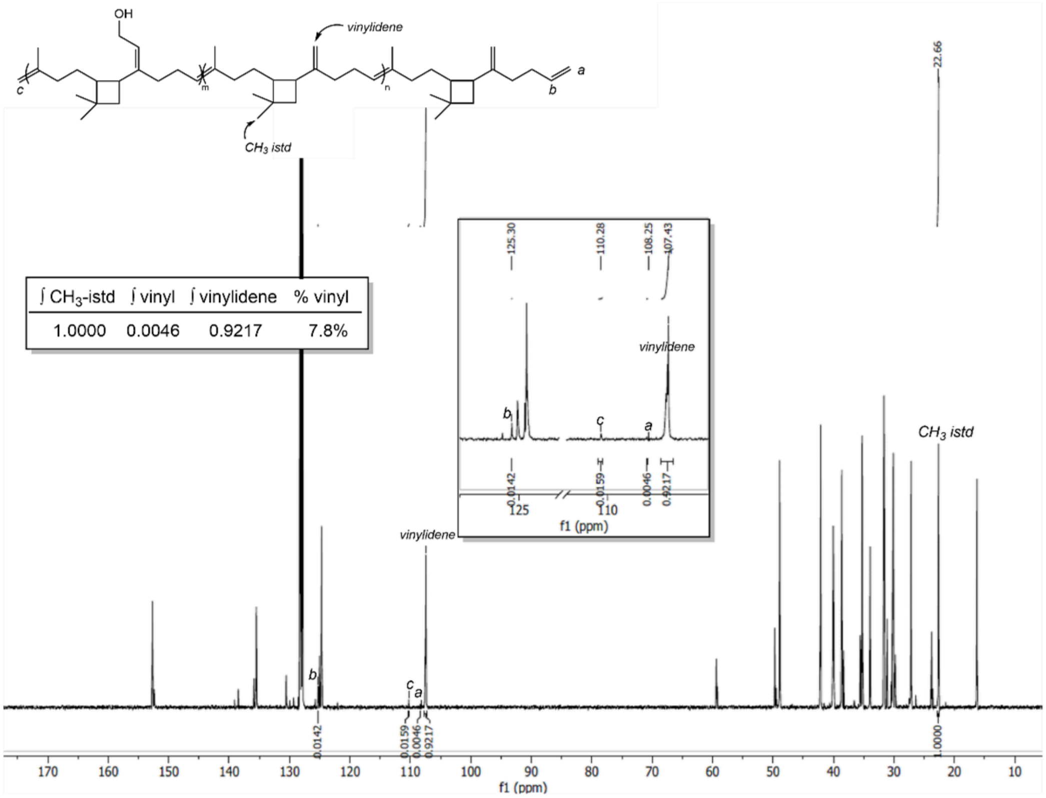

Figure S5C. Chain end analysis by quantitative $^{13}C\{^1H\}$ NMR of HTPCR(1). Inset: signals diagnostic of vinyl-termination utilized for quantification.

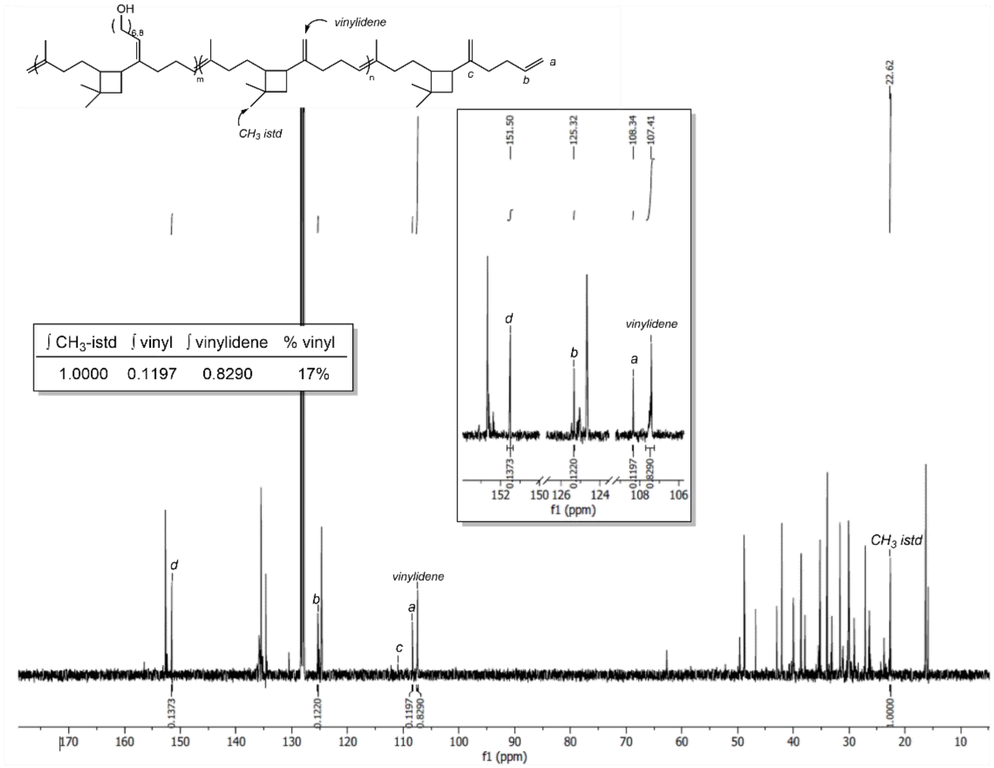

Figure S5D. Chain end analysis by quantitative $^{13}C\{^1H\}$ NMR of HTPCR(6,8). Inset: signals diagnostic of vinyl-termination utilized for quantification.

Assessment of Metathesis Processes Leading to Vinyl Termination. Evidence for the dominant polymer structures being α,ω-disubstituted linear polycaryophyllenes, as depicted in the schemes throughout the main text, is predicated on the NMR quantitation indicating that the majority of the vinylidene signals (>92%) within acetoxy-terminated polycaryophyllene and HTPCR(1) are retained in the polymers post-synthesis (Figure S5B and S5C). Further, these vinylidene signals are =$\underline{C}H_2$ carbons as indicated in the APT NMR (Figure S1F). Thus, any branching from the vinylidene positions does not substantiate the majority of the microstructure of the polymers synthesized and utilized for the production of polyurethanes.

In consideration of the metathesis mechanisms accounting for 6-8% of the vinyl content (in the case of acetoxy-terminated polycaryophyllene and HTPCR(1)) or 17% of the vinyl content (in the case of HTPCR(6,8)) of the microstructure, several processes may be operative from propagating ruthenium carbene species:

Scenario 1: In the first scenario, ROMP polymerization occurs, after which a propagating Ru carbene engages an exocyclic vinylidene of an already formed polycaryophyllene, as shown in Scheme S1. Chain termination with CTA would then produce the branched polyol.

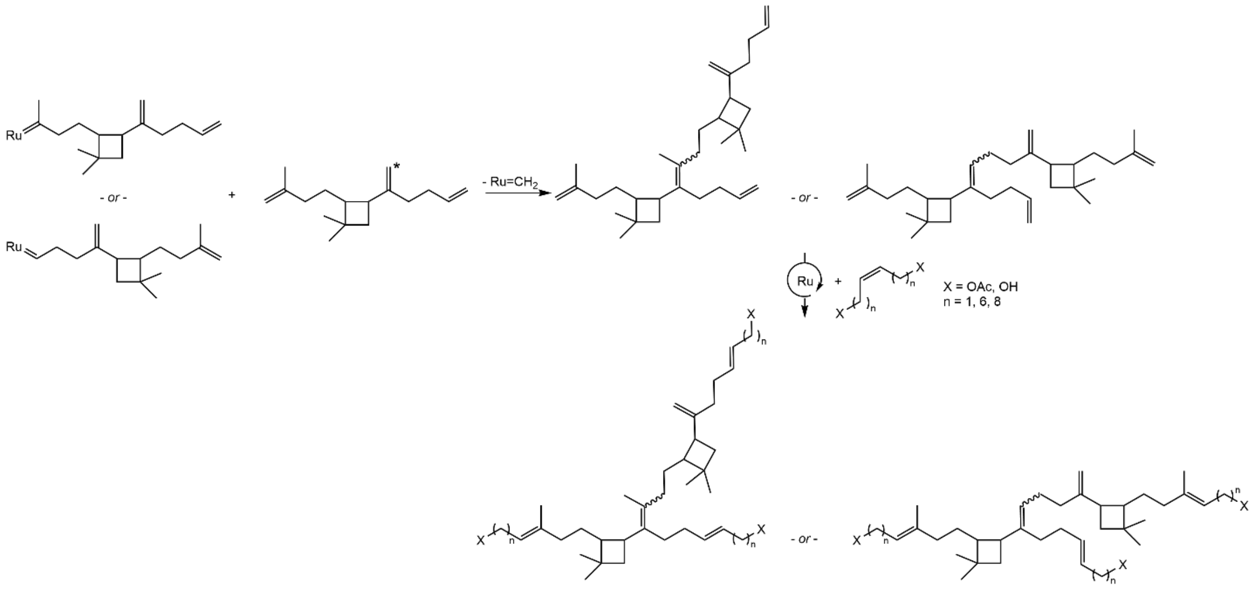

Scheme S1. Products arising from ROMP polymerization and branching prior to CTA termination. A single unit of polycaryophyllene is shown for clarity. The vinylidene involved in branching is indicated with a *.

If branching preceded CTA termination, there would either be no correlation or an inverse correlation between activity of the CTA and the vinyl content of the resulting polymer; that is, since the branching process produces more vinylated chain ends than with purely α,ω-functionalization, a more active polyol CTA would more readily convert the termini to allyl alcohols. However, the opposite trend is observed in the synthetic data, whereby the use of the more active cis-7-hexadec-6-ene-1,16-diol in the synthesis resulted in *more* vinyl content than with less active cis-1,4-diacetoxy-2-butene or cis-1,4-dihydroxy-2-butene (Figure S5B-D). This observation is counterintuitive to that expected with the branching process as terminal vinyl groups should react faster with highly active CTA than the geminal substituted vinylidenes, based on steric arguments on rates of metathesis. Other data excluding this mechanism of polymerization giving rise to a predominantly branched microstructure prior to CTA termination are the observations that the polyols are thermoplastic, paralleling that seen by Mecking in the seminal synthesis of polycaryophyllene[2]. Predominant polycaryophyllene branching prior to CTA termination has the potential to lead to thermosets, which were not observed; intramolecular processes were also not observed in the prior art and were not identified in these data.

Scenario 2: In the second scenario, ROMP and CTA termination precludes branching, such that the vinylidenes of an already formed diol engages with the propagating Ru carbene, as depicted in Scheme S2.

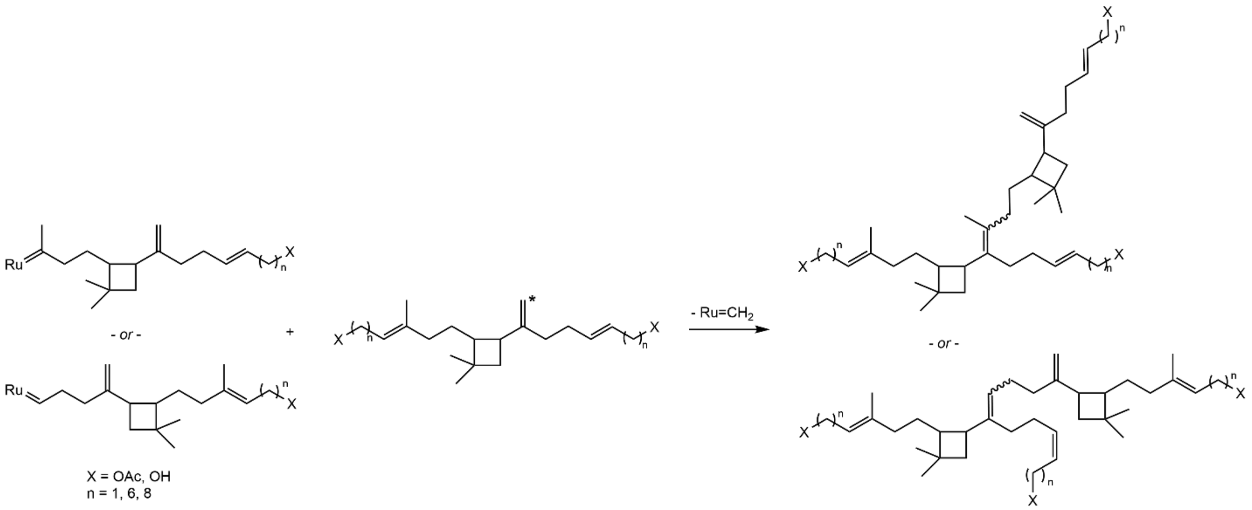

Scheme S2. Products arising from ROMP polymerization and CTA termination occurring with branching. A single unit of polycaryophyllene is shown for clarity. The vinylidene involved in branching is indicated with a *.

If predominantly operative, this process would result in an OH functionality greater than 2, which was not observed in the HTPCR(1) used for the synthesis of the polyurethanes. Further, if this process predominated, it is expected that the reaction of the isolated HTPCR(1) with G2 alone would lead to an increase in molecular weight of the polyol and erosion of dispersity as branching occurs. This experiment was conducted in the decomposition processes detailed in Table 6, entries 1 and 2, in which HTPCR(1) was reacted with 1 wt % G2 at room temperature or 50 °C for 48 hours. In these experiments, no substantial change in molecular weight or dispersity of the polyol was observed, suggesting that branching between two functionalized polymer units does not predominate.

Scenario 3: A third pathway is that ROMP and CTA termination kinetically outcompete branching processes entirely. In this scenario, the Ru allyl alcohol carbenes would be responsible for increasing the vinyl content of the polymer post-synthetically, as shown in Scheme S3.

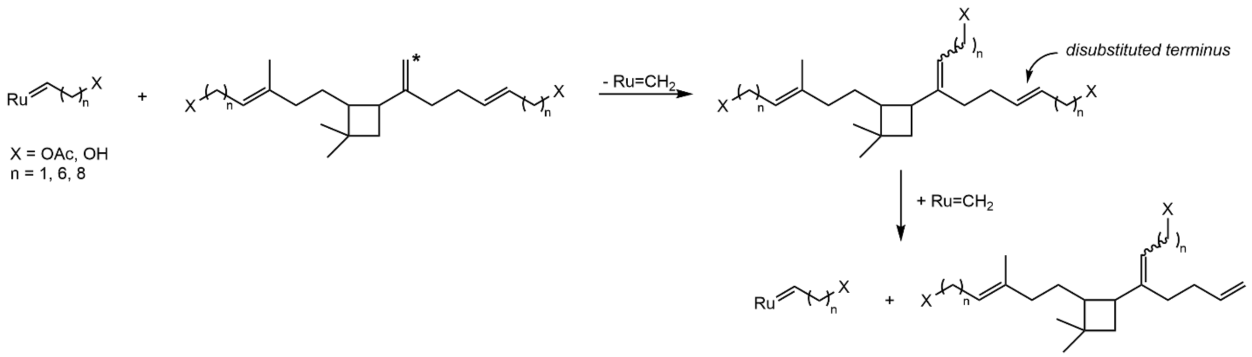

Scheme S3. Products arising from ROMP polymerization and CTA termination outcompeting branching. A single unit of polycaryophyllene is shown for clarity. The vinylidene involved in metathesis is indicated with a *.

Kinetically slower reaction of the ruthenium carbene with vinylidenes of the α,ω-disubstituted linear polycaryophyllenes would add allyl alcohol functionalities to the internal positions of the chain and return a ruthenium methylidene to the active catalyst. Exchange of the ruthenium methylidene with 1,2-disubstituted allyl alcohol termini of the polymer would then result in terminal vinyl groups in the resulting microstructure; exchange at the 1,2-disubstituted terminus should also be favorable compared to exchange at the trisubstituted olefin terminus due to steric constraints of metathesis. Potential exchange of the ruthenium methylidene with vinylidenes would also result in degenerate speciation. This mechanism accounts for the observation of increased vinyl content seen with the more active cis-7-hexadec-6-ene-1,16-diol, given that the ruthenium allyl alcohol carbene appears more active in metathesis than the shorter CTAs, leading to more termini exchange. This mechanism also accounts for the observation that, in the chain end characterization of HTPCR(6,8) of Figure S5D, monosubstituted vinyl chain termination is observed in a significantly higher quantity than geminal-substituted vinyl termination. An exchange process of this type also justifies the observed microstructure of the acetoxy-terminated polycaryophyllene, in which 5.5% of the acetoxy groups can be found on the vinylidene positions (Figure S5A and Figure S1E).

Validation of the metathesis exchange of vinylidene groups with exogenous CTA, as conducted in **Section II. Post-Synthetic Metathesis Activity of HTPCR(1)** (*vide infra*)**,** also supports the

exchange mechanism of Scenario 3. Subjection of predominantly α,ω-disubstituted HTPCR(1) to excess cis-1,4-diacetoxy-2-butene in the presence of G2 lead to a 12% consumption of vinylidenes and 8% increase in acetoxy content at the vinylidene positions; however, a 1% increase in vinyl termination was also observed (Figure S6A), potentially arising from the pathway detailed in Scheme S3. While this hypothesized background activity could give rise to hydroxyl functionalities greater than 2 in the bulk polymer, the reactivity of the vinylidenes with CTA appears significantly slower than chain termination during the ROMP process. These data indicate that rigorously disubstituted, linear α,ω-acetoxy-terminated polycaryophyllene and HTPCR(1) predominates in the microstructure.

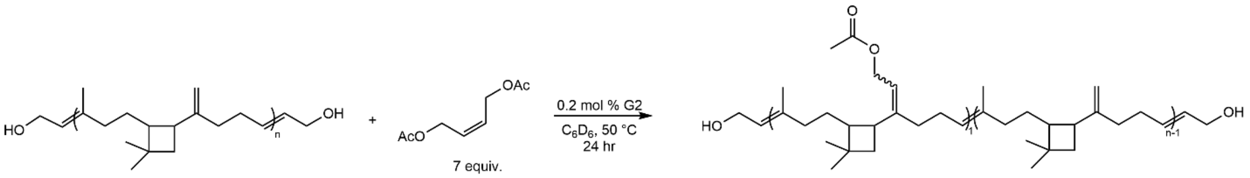

Post-Synthetic Metathesis Activity of HTPCR(1). In order to verify the metathesis activity of the vinylidenes, post-synthetic cross-metathesis between HTPCR(1) and cis-1,4-diacetoxy-2-butene was conducted. To a J Young tube in the glove box was added 200 mg (Mn = 2.891 kg/mol, Đ = 1.4, 0.06918 mmol) of HTPCR(1) dissolved in 600 μL of benzene-d_6. An initial quantitative $^{13}C\{^1H\}$ NMR was taken, after which the tube was returned to the glove box and the contents added to a vial. To the vial was then added 83.3 mg (0.484 mmol, 7 equiv.) of cis-1,4-diacetoxy-2-butene, after which 100 μL of a stock solution containing 12 mg of G2 in 1 mL of benzene-d_6 was added. The vial contents were mixed to homogeneity and transferred to a J Young tube, after which the tube was sealed and added to an oil bath at 50 °C for 24 hours. After 24 hours the degree of vinylidene consumption was quantified by quantitative $^{13}C\{^1H\}$ NMR. The comparative quantitative $^{13}C\{^1H\}$ NMR spectra before and after cross-metathesis are given in Figure S6A, and integration of the relative number of vinylidenes to a methyl group of the cyclobutyl ring before and after the reaction indicated a 12% reduction in vinylidene content.

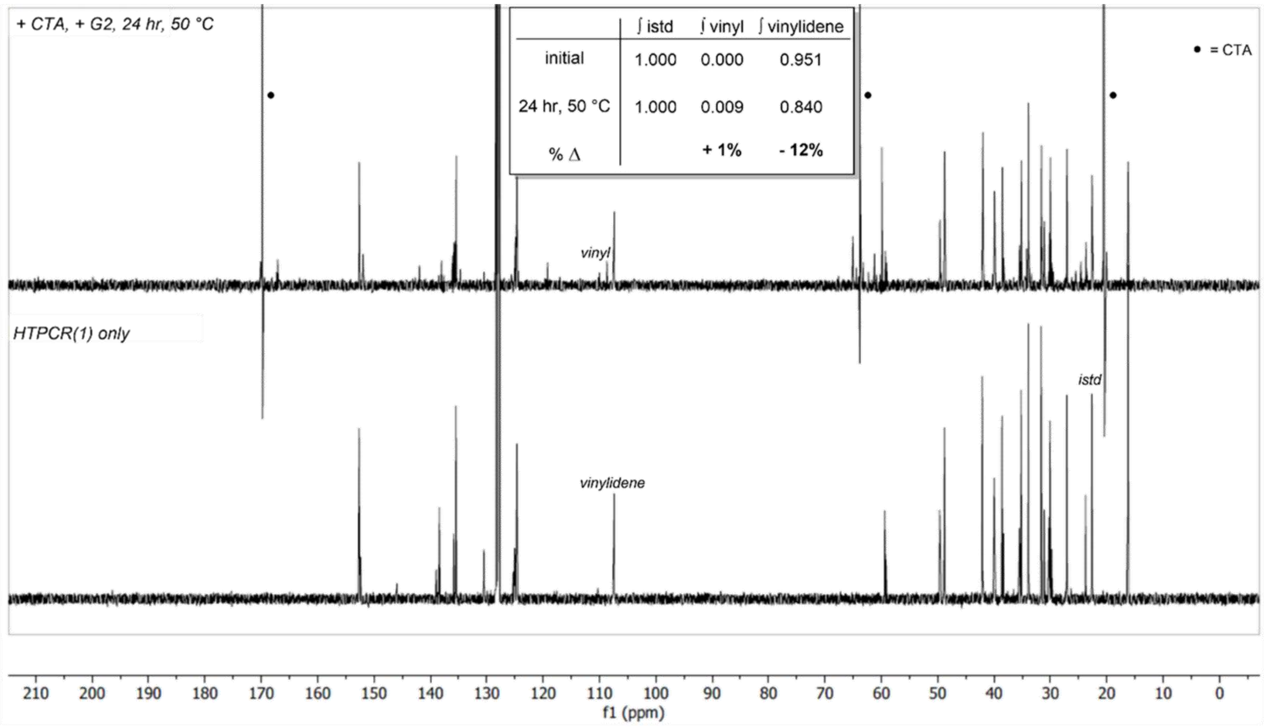

Figure S6A. Comparative quantitative $^{13}C\{^{1}H\}$ NMR spectra of HTPCR(1) before (bottom) and after (top) cross-metathesis with CTA. Relative integrations of the vinylidene signals to the methyl group of the cyclobutyl ring are given in the inset.

Confirmation of acetoxy functionality incorporation was provided by isolation of the resulting polymer in the following procedure. The contents of the J Young tube were dispersed in 5 mL of methanol, after which the polymer precipitate was collected by decantation of the methanol wash. The precipitate was washed two more times with 5 mL portions of methanol, after which the precipitated polymer was dried under vacuum. A quantitative $^{13}C\{^{1}H\}$ NMR spectrum and an IR spectrum of the polymer was then obtained (Figure S6B and C). In the former, the carbonyl carbon was identified at ca. 171 ppm, integrating to 8% of the sample; in the latter, the carbonyl stretch at 1743 cm^{-1} was identified.

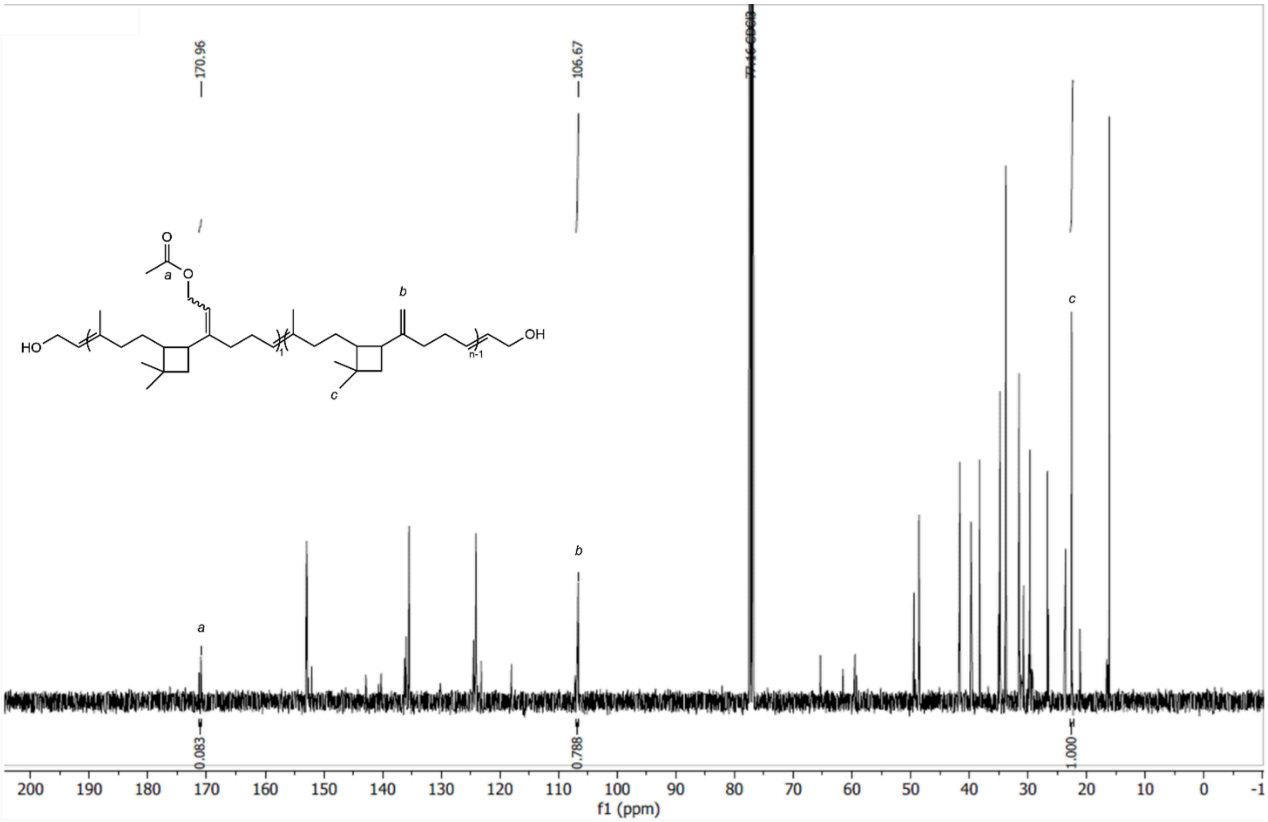

Figure S6B. Annotated quantitative $^{13}C\{^{1}H\}$ NMR spectrum of isolated HTPCR(1) after cross-metathesis with CTA.

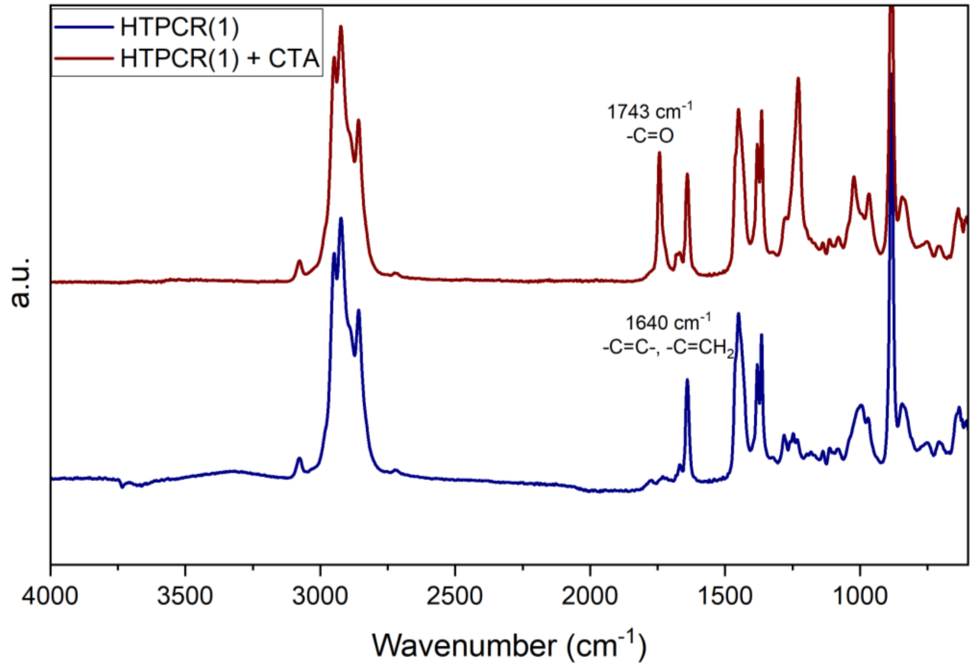

Figure S6C. Annotated ATR-IR spectrum of isolated HTPCR(1) before (blue, bottom) and after (red, top) cross-metathesis with CTA.

DSC Data for HTPCR(1) and HTPCR(6,8). The glass transition temperatures of the polyols were determined through DSC analysis using parameters detailed in **Section I. General Methods.** The DSC spectra used to elucidate the glass transition temperatures are given in Figure S7.

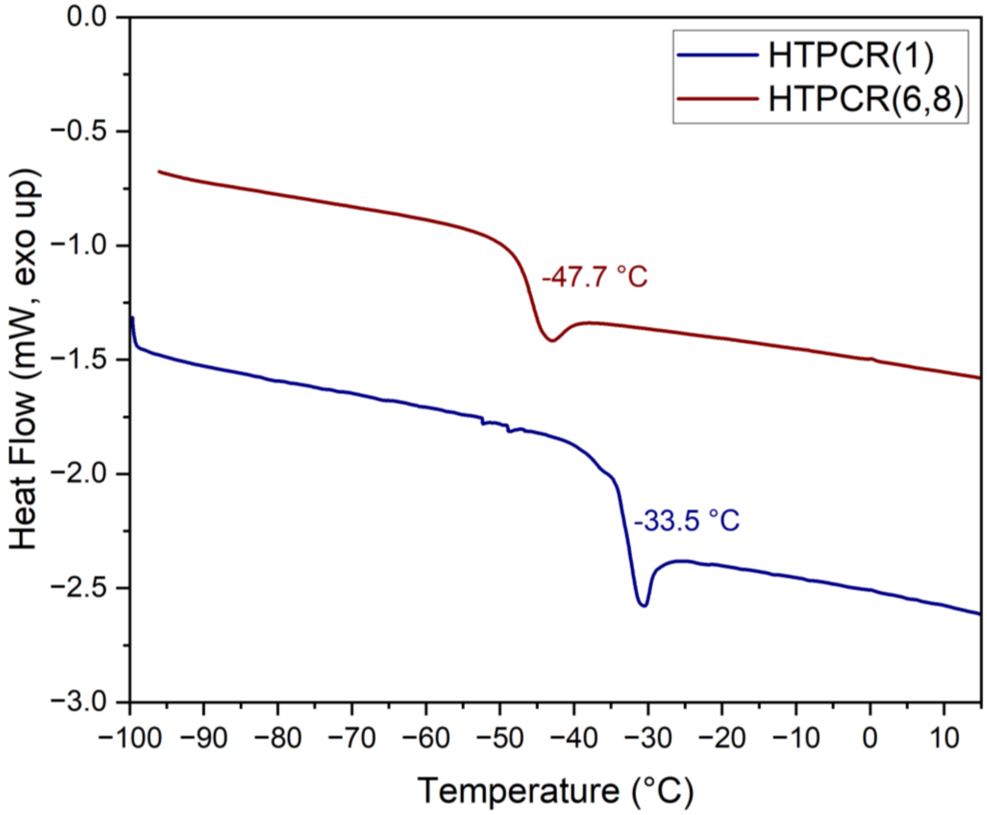

Figure S7. DSC data used to identify the glass transition temperatures for HTPCR(1) (blue trace) and HTPCR(6,8) (red trace).

TGA Data for HTPCR(1) and HTPCR(6,8). The decomposition temperatures of the polyols were determined through TGA analysis using parameters detailed in **Section I. General Methods.** The TGA spectra are given in Figure S8.

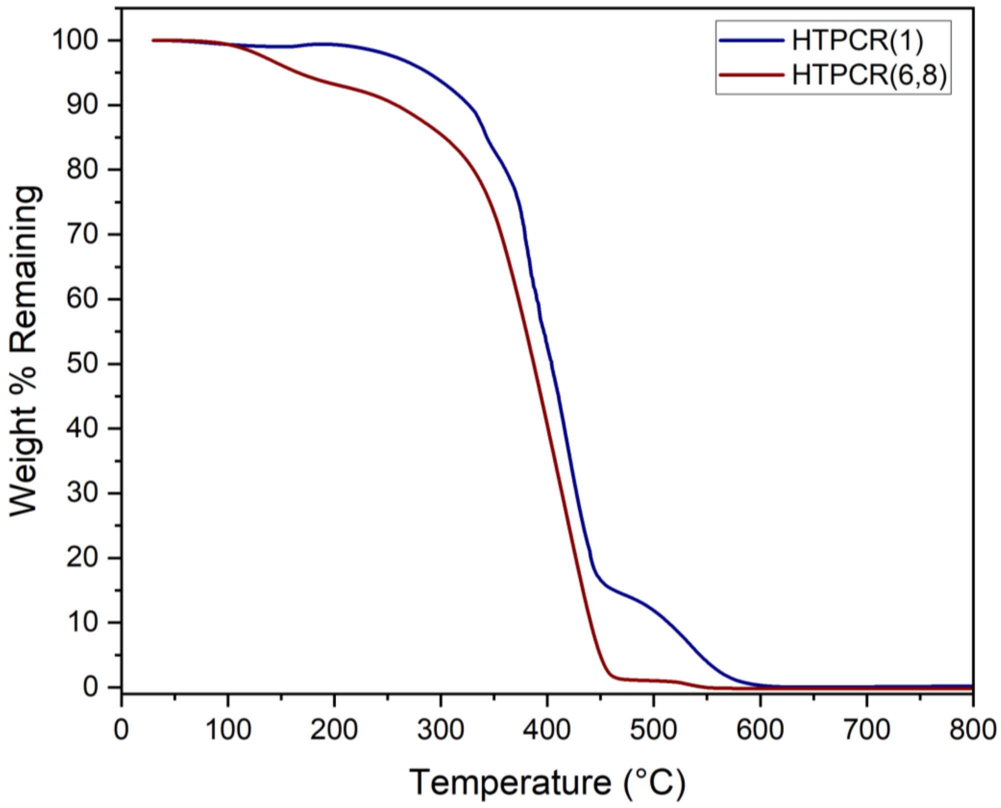

Figure S8. Inert atmosphere TGA spectra for HTPCR(1) and HTPCR(6,8).

III. Synthesis and Characterization of HTPCR-Based Polyurethanes

General Cast-Cure Procedure for Elastomers. To a vial in the glove box was added 4.000 grams (0.833 mmol) of HTPCR(1). DBTDL (25 µL, 0.0416 mmol, 0.05 equiv.) was added to the vial with a syringe. To a separate vial was added 1.833 mmol (2.2 equiv.) of di- or triisocyanate and 3 mL of toluene. The toluene solution was poured into the polyol vial, and the vial was capped and quickly agitated until dissolution of the components. The homogeneous solution was poured into a 2.5 inch diameter aluminum pan and left at room temperature to cure. The resulting pans are shown in Figure S9A.

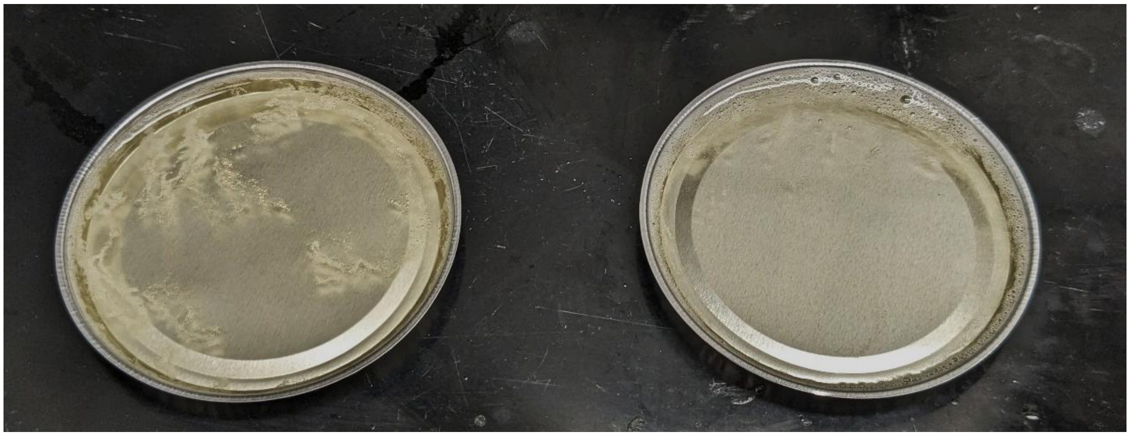

Figure S9A. Images of cast cures of HTPCR-TDI (right) and HTPCR-polyHDI (left) in aluminum pans.

After 24 hours, the solidified contents were cut from the pans using a dogbone dye cutter with dimensions measuring 20.00 mm length, 5.00 mm width, and 1.50 mm thickness. The resulting cuttings were sonicated in water for 5 minutes, then acetone for 2 minutes to remove unreacted polyisocyanate, then dried under vacuum. The removal of excess isocyanate was tracked by IR by the disappearance of the peak at 2271 cm^{-1} corresponding to the residual isocyanate functional group. Representative IR spectra for the purification of HTPCR-TDI are given in Figure S9B.

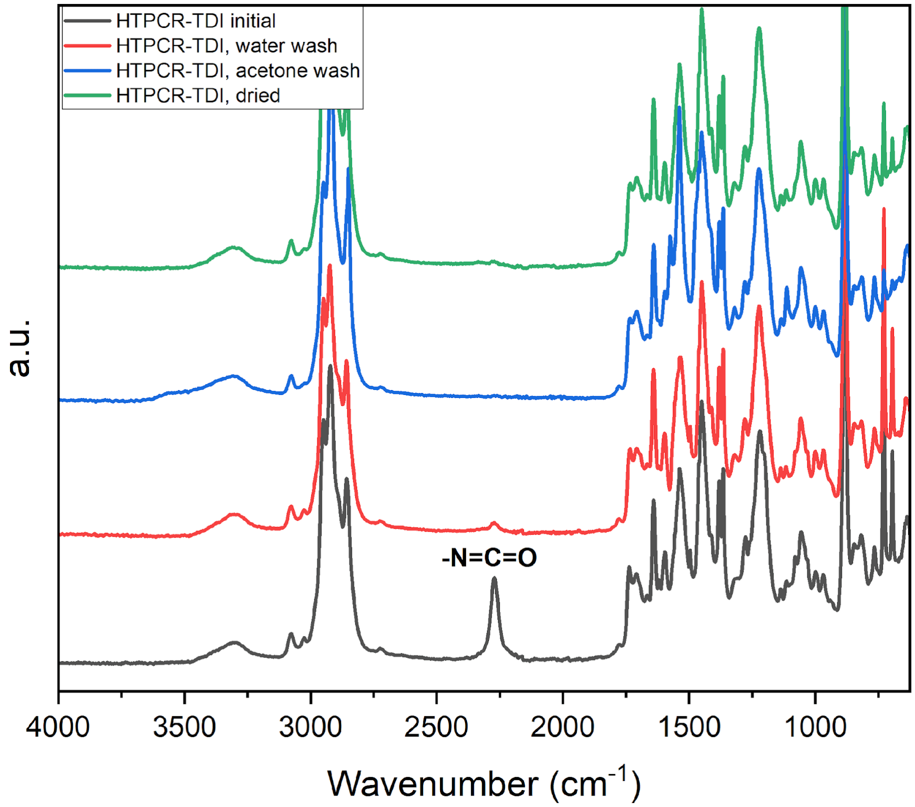

Figure S9B. ATR-IR spectra taken at different steps of the purification process to remove excess isocyanate in the synthesis of HTPCR-TDI.

The polyurethanes were then characterized by ATR-IR, tensile testing, DSC, DMA, and TGA. HTPCR-TDI was also analyzed by NMR and GPC due to its solubility in organic solvents. A gelation test was conducted on HTPCR-polyHDI in order to estimate the degree of crosslinking.

General Cast-Cure Procedure for Adhesives. To a vial in the glove box was added 4.000 grams (0.833 mmol) of HTPCR(1). DBTDL (25 µL, 0.0416 mmol, 0.05 equiv.) was added to the vial with a syringe. To a separate vial was added 0.833 mmol (1 equiv.) of di- or triisocyanate and 3 mL of toluene. The toluene solution was poured into the polyol vial, and the vial was capped and quickly agitated until dissolution of the components. The homogeneous solution was poured into a 2.5 inch diameter aluminum pan and added to an oven at 110 °C to cure for 24 hours.

After 24 hours, the solidified contents were scraped out of the pans using a spatula and collected in respective vials. The polyurethanes were then characterized by ATR-IR, lap shear testing, DSC, and TGA. HTPCR-TDI was also analyzed by NMR and GPC due to its solubility in organic solvents. A gelation test was conducted on adhesive-like HTPCR-polyHDI in order to estimate the degree of crosslinking.

NMR and GPC Spectroscopic Data for HTPCR-TDI Thermoplastics. HTPCR-TDI was characterized by ^{1}H, $^{13}C\{^{1}H\}$, and ^{1}H-^{1}H COSY NMR. The data are reported as follows: ^{1}H NMR (400 MHz, benzene-d_6, 25 °C): δ 8.09 (br s, 4H); 7.54 (br s, 4H); 7.36 (s, 1H); 6.96 (s, 1H); 6.89 (br s, 3H); 6.79 (br s, 2H); 6.09 (br s, 2H); 6.01 (app. m, 1H); 5.57 (br s, 12H); 5.35 (m, 48 H); 4.98 (m, 102H); 4.72 (m, 12H); 3.36 (m, 2H); 2.50 (m, 50H); 2.31-2.02 (m, 372 H); 1.84 (t, 58H); 1.77 (s, 43H); 1.65 (m, 300H); 1.30 (m, 26H); 1.08 (m, 355H). The ^{1}H NMR spectrum is given in Figure S10A. $^{13}C\{^{1}H\}$ NMR (101 MHz, benzene-d_6, 25 °C): δ 152.67, 135.92, 135.51, 128.30, 128.06, 127.82, 125.04, 124.67, 107.45, 49.67, 48.82, 42.10, 40.01, 38.62, 35.53, 35.21, 33.93, 31.63, 31.14, 30.08, 27.12, 23.76, 22.62, 16.26. $^{13}C\{^{1}H\}$ NMR spectrum is given in Figure S10B. ^{1}H-^{1}H COSY NMR was used to establish correlations between chain end groups and carbamate-adjacent groups, as indicated in Figure S10C. Molecular weight and dispersity were determined by GPC; the chromatogram is given in Figure S10D.

Assignment of the integration of the ^{1}H NMR spectrum of HTPCR-TDI was based on the obtained molecular weight of the PU by GPC (14.480 kg/mol) as well as the molecular weight of HTPCR(1) obtained by GPC (4820 kg/mol) and ^{1}H NMR. Based on the molecular weights of the polyol and PU, there are three HTPCR(1) units and either two or three TDI units in the PU chain for a total molecular formula of $C_{1044}H_{1704}N_4O_7$ (14.400 kg/mol, two TDI units) or $C_{1053}H_{1710}N_6O_9$ (14.574 kg/mol, three TDI units), in excellent agreement with the GPC results. Three HTPCR units give rise to 12 protons associated with the methylenes adjacent to the urethane oxygens or terminal

oxygens at 4.70 ppm, setting a starting point for assignment of integrations of the ^{1}H NMR spectrum.

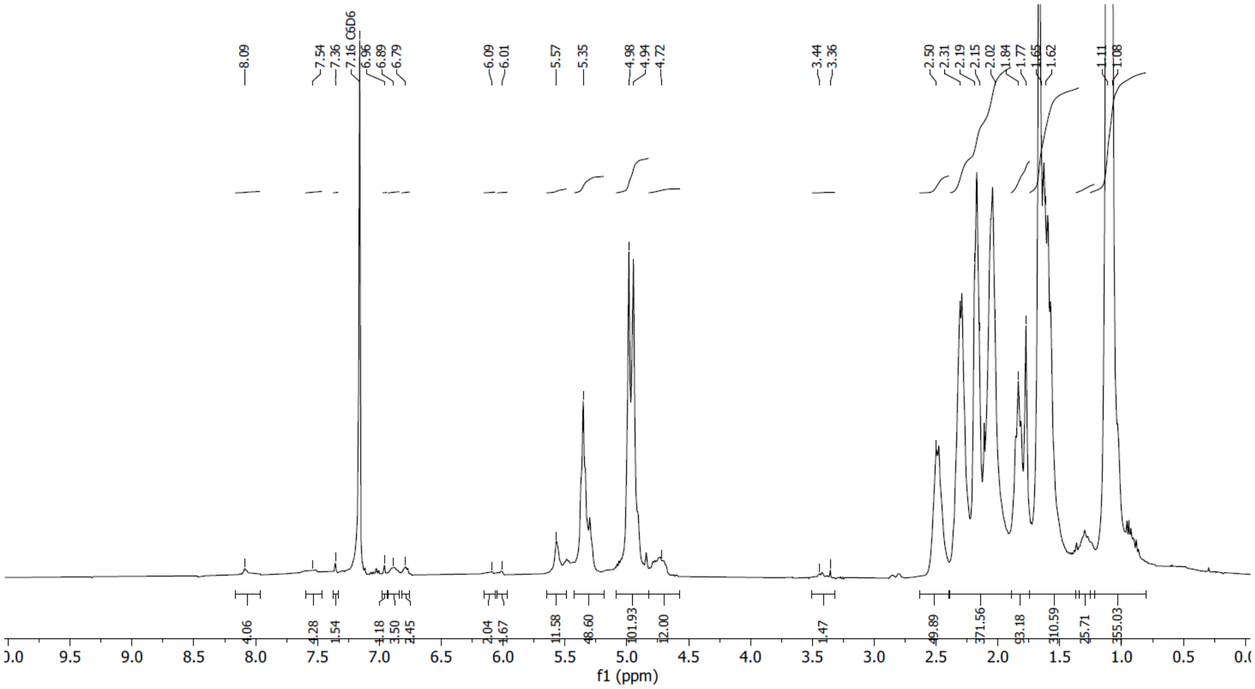

Figure S10A. ^{1}H NMR (benzene-d_6, 25 °C) spectrum of HTPCR-TDI elastomer.

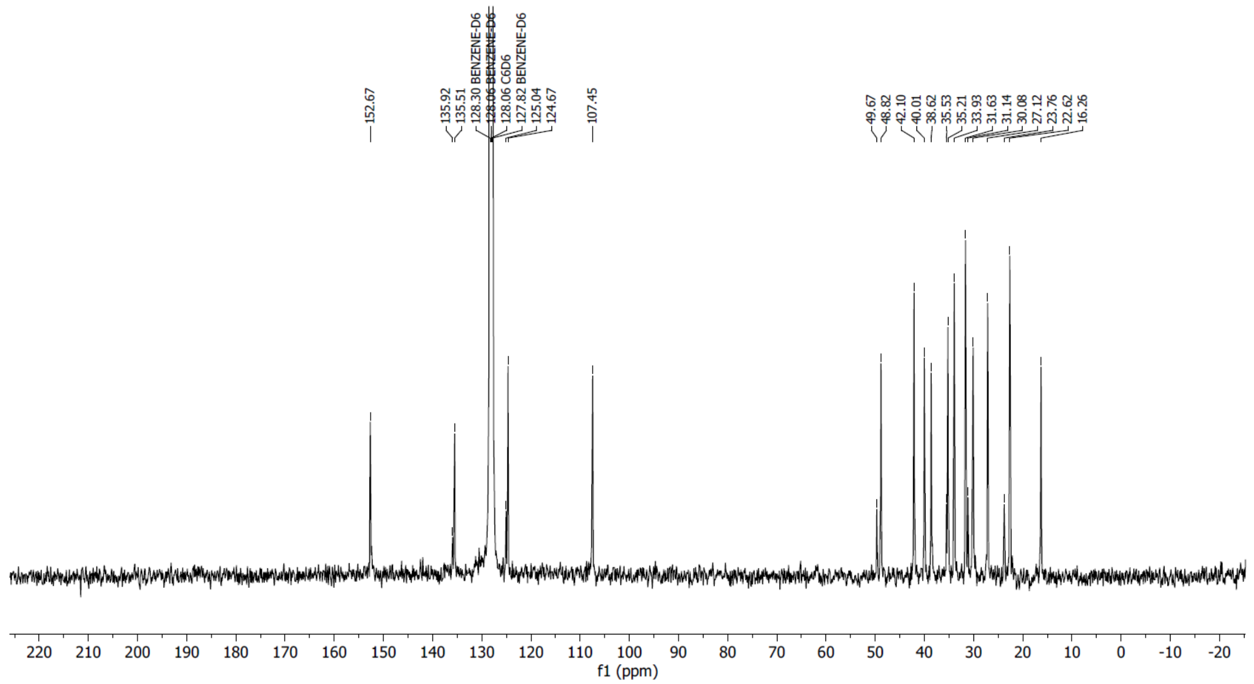

Figure S10B. $^{13}C\{^{1}H\}$ NMR (benzene-d_6, 25 °C) spectrum of HTPCR-TDI elastomer.

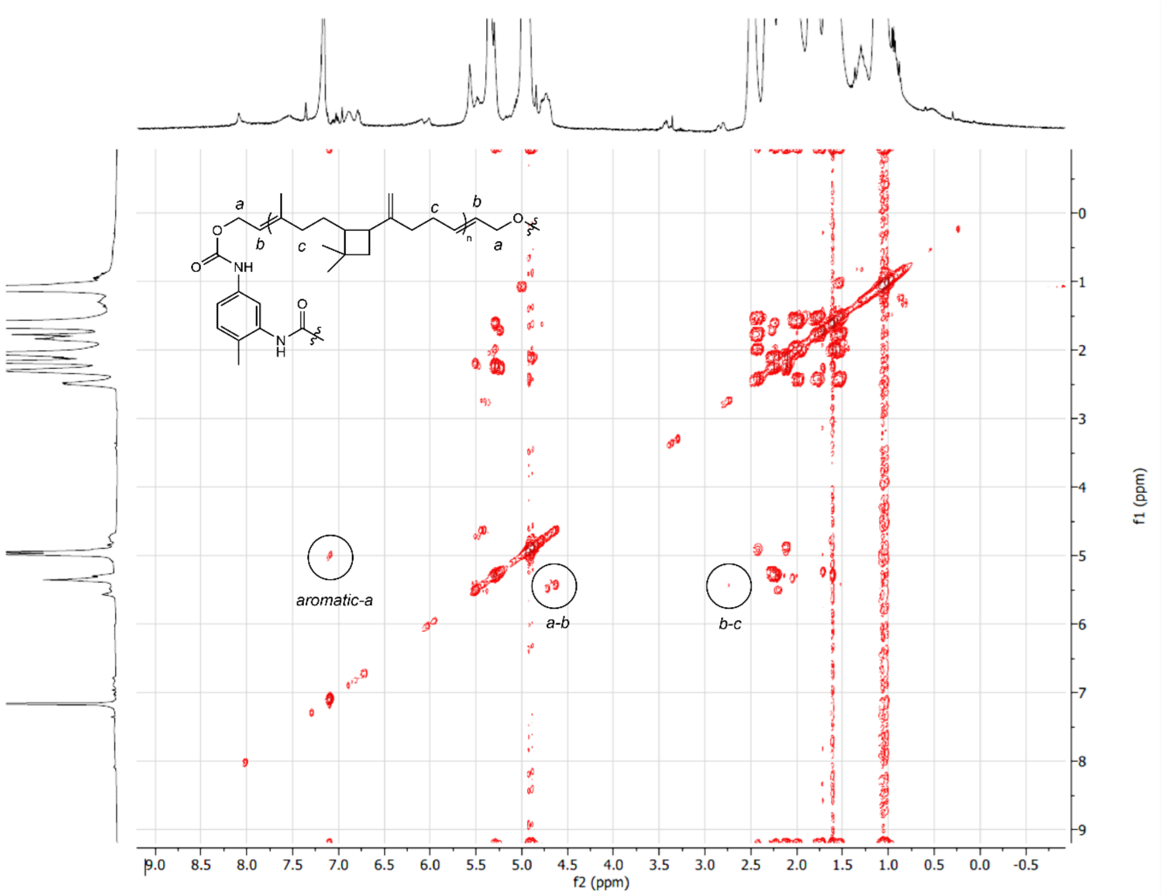

Figure S10C. Annotated ^{1}H-^{1}H COSY NMR (benzene-d_6, 25 °C) spectrum of HTPCR-TDI elastomer.

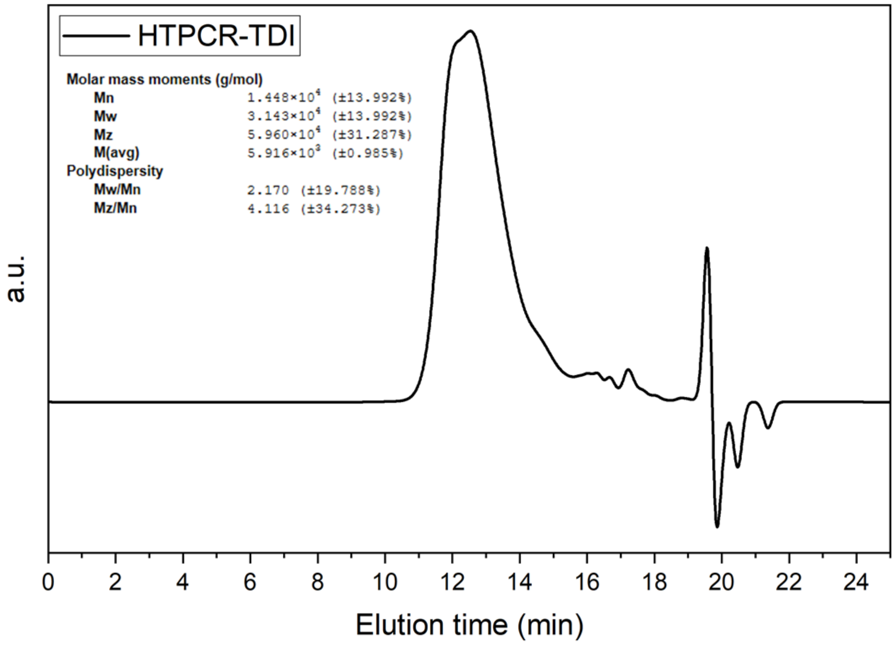

Figure S10D: GPC Chromatogram of HTPCR-TDI elastomer (M_n = 14.480 kg/mol, Đ = 2.2).

Adhesive HTPCR-TDI was also characterized by NMR spectroscopy as well as GPC and was found to have identical spectroscopic signatures to that of the elastomer material. The ^{1}H and ^{13}C NMR spectra of HTPCR-TDI adhesive is given in Figure S10E and Figure S10F, respectively, and the GPC is given in Figure S10G.

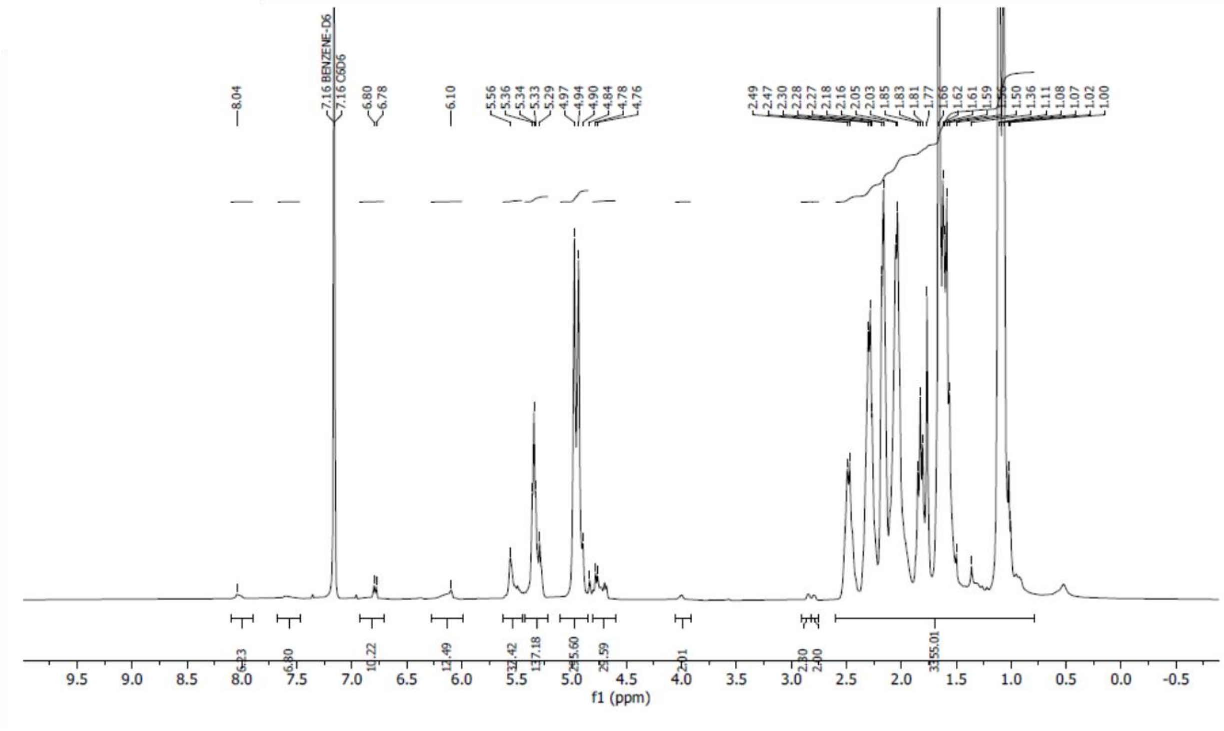

Figure S10E. ^{1}H NMR (benzene-d_6, 25 °C) spectrum of HTPCR-TDI adhesive.

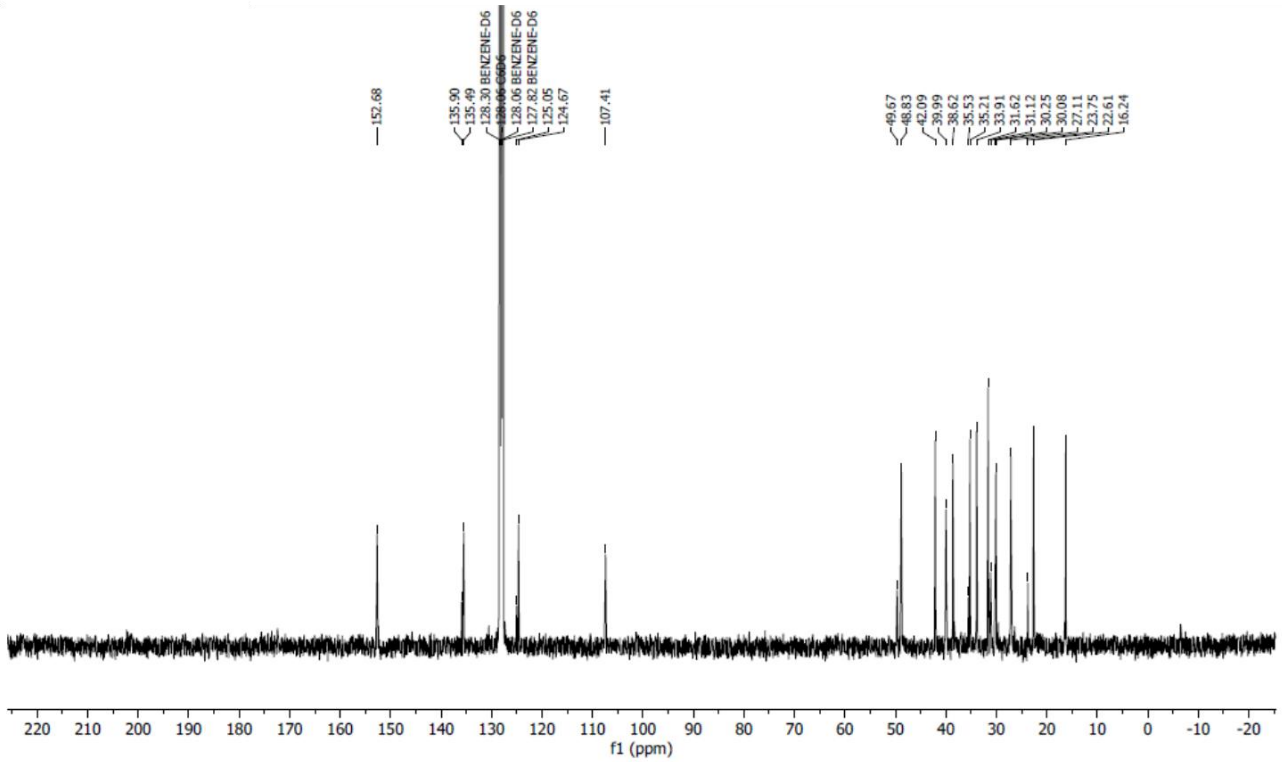

Figure S10F. $^{13}C\{^{1}H\}$ NMR (benzene-d_6, 25 °C) spectrum of HTPCR-TDI adhesive.

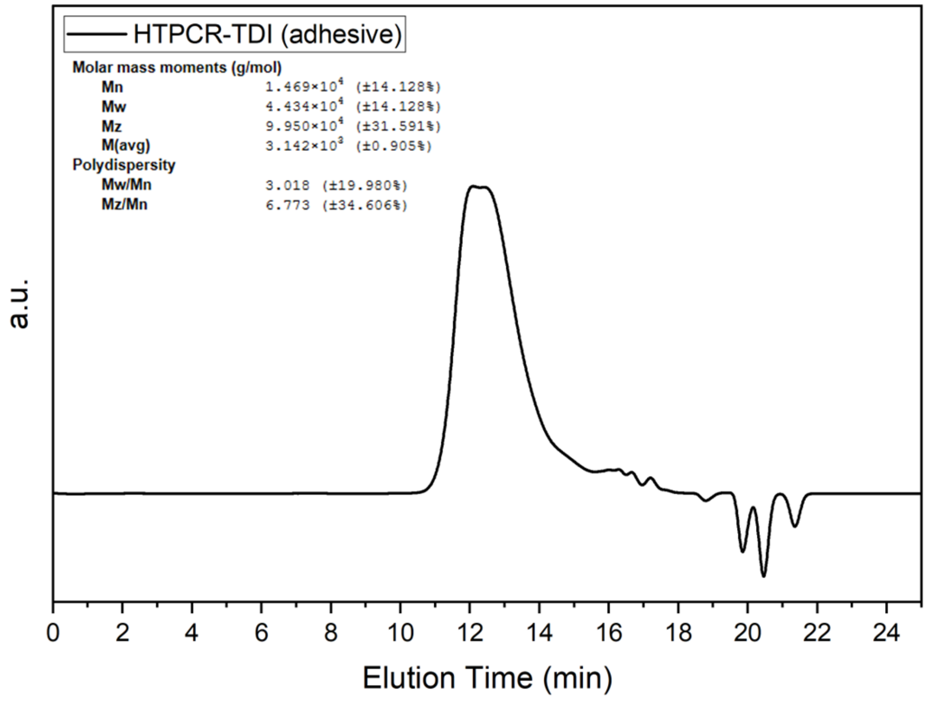

Figure S10G: GPC Chromatogram of HTPCR-TDI adhesive (M_n = 14.469 kg/mol, Đ = 3.0).

ATR-IR Data for HTPCR-TDI and HTPCR-polyHDI Variants. The purity of the cures and the formation of urethane linkages in the thermoplastics and thermosets synthesized was established by ATR-IR. The spectroscopic data is reported as follows: ATR-IR of HTPCR-TDI: (cm^{-1}, assignment): 3310 cm^{-1}, -NH- and terminal -OH, 1733 cm^{-1}, -C=O, 1640 cm^{-1}, -C=C- and -$C=CH_2$. ATR-IR of HTPCR-polyHDI: (cm^{-1}, assignment): 3310 cm^{-1}, -NH- and terminal -OH, 1733 cm^{-1}, -C=O, 1640 cm^{-1}, -C=C- and -$C=CH_2$.

The annotated ATR-IR spectra of HTPCR-TDI and HTPCR-polyHDI are given in Figure S11A and Figure S11B, respectively.

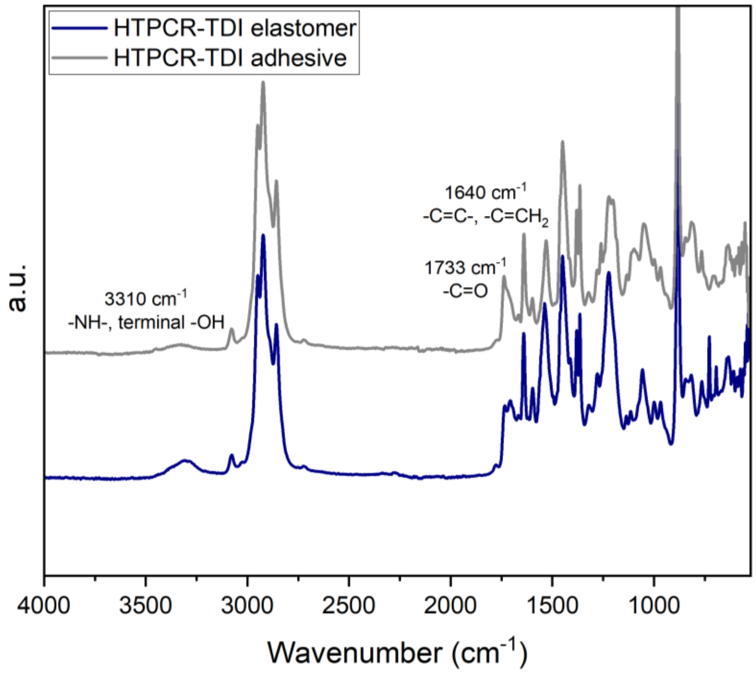

Figure S11A. Annotated ATR-IR spectra of HTPCR-TDI adhesive (top) and HTPCR-TDI elastomer (bottom).

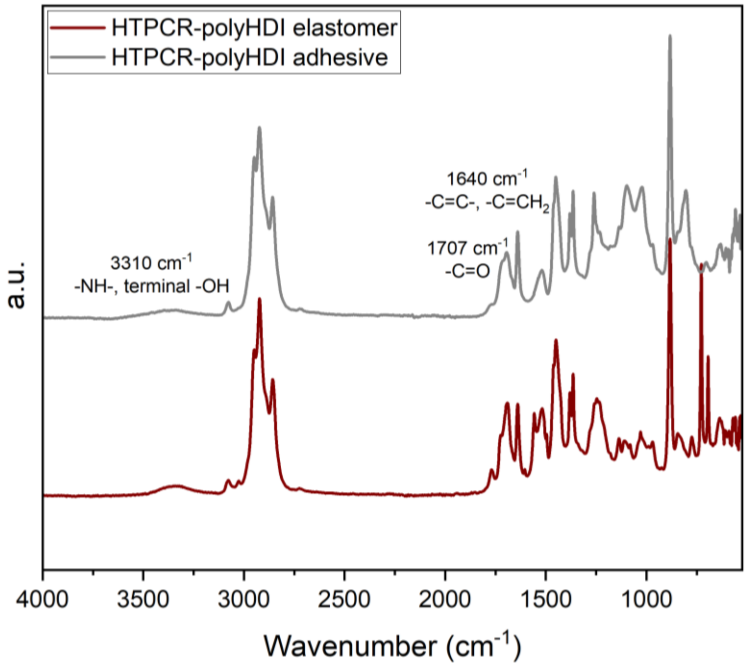

Figure S11B. Annotated ATR-IR spectra of HTPCR-polyHDI adhesive (top) and HTPCR-polyHDI elastomer (bottom).

Gelation Test for Crosslinking of HTPCR-polyHDI Thermosets. A 379.2 mg sample of HTPCR-polyHDI elastomer was cut from the synthesized cast cure films, while a 313.1 mg sample of HTPCR-polyHDI adhesive was cut from the synthesized gel material. The samples were immersed in a scintillation vial containing 10 mL of THF and a PTFE-coated stir bar. The vials were capped and added to a heating block at 65 °C, and were heated with stirring for 24 hours. After 24 hours, the solvent and solubilized material was decanted from the thermosets, and solvent on the surface of the cures was removed by placing it on an absorbent (paper) towel. The samples were then re-weighed on an analytical balance, in which a final mass of 297.2 mg was obtained for the elastomer, and 183.0 mg was obtained for the adhesive. Percent swell was calculated using the following equation:

$$\% \text{ crosslinking} = m_2/m_1 \times 100\%$$

where m_1 = mass of cure before immersion in mg and m_2 = mass of cure after immersion in mg. The percent crosslinking content was found to be 78% for the elastomer and 58% for the adhesive.

TGA Data for HTPCR-TDI and HTPCR-polyHDI variants. The decomposition temperatures of the polyurethane cures were determined through TGA analysis using parameters detailed in **Section I. General Methods.** The TGA spectra are given in Figure S12, with the spectrum of HTPCR(1) provided for comparison.

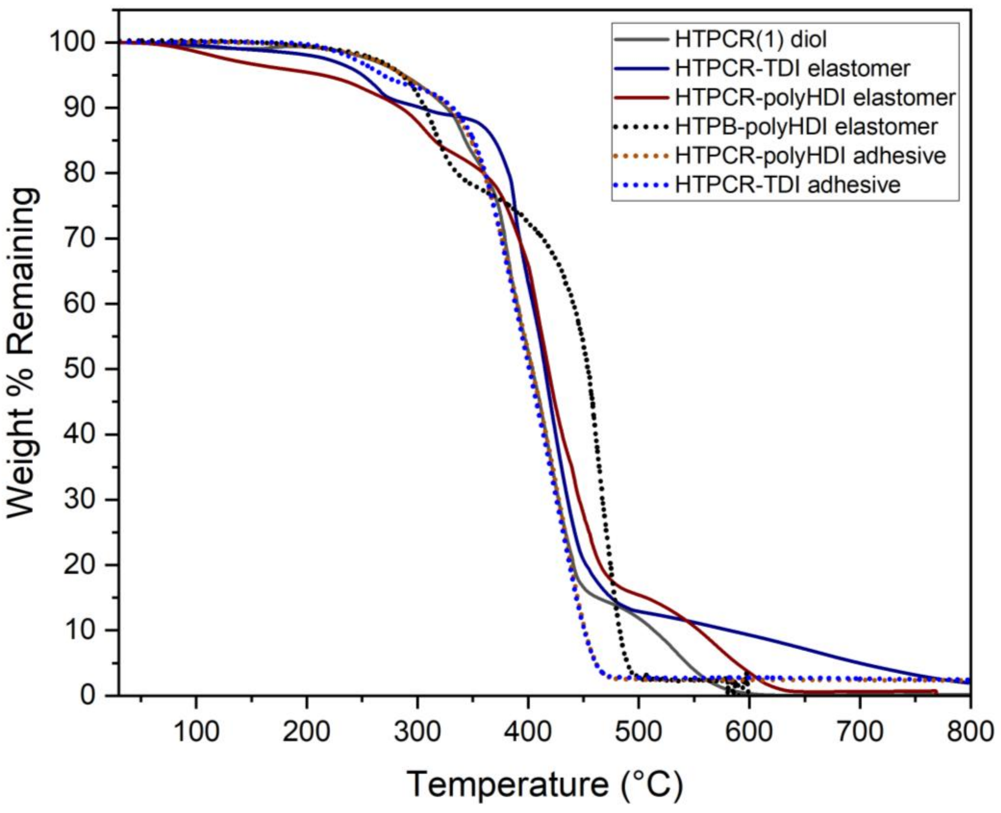

Figure S12. Annotated inert atmosphere TGA spectra of HTPCR-TDI and HTPCR-polyHDI variants.

DSC Data for HTPCR-TDI and HTPCR-polyHDI. Confirmation of either thermoplastic behavior for HTPCR-TDI or thermosetting behavior for HTPCR-polyHDI elastomers was obtained through DSC, using parameters detailed in **Section I. General Methods**. For HTPCR-TDI, a melting event

was identified at 223 °C, indicative of thermoplastic behavior. For HTPCR-polyHDI, no melting or crystallization events were found in the temperature window, consistent with thermosetting behavior. The DSC spectra are given in Figure S13A.

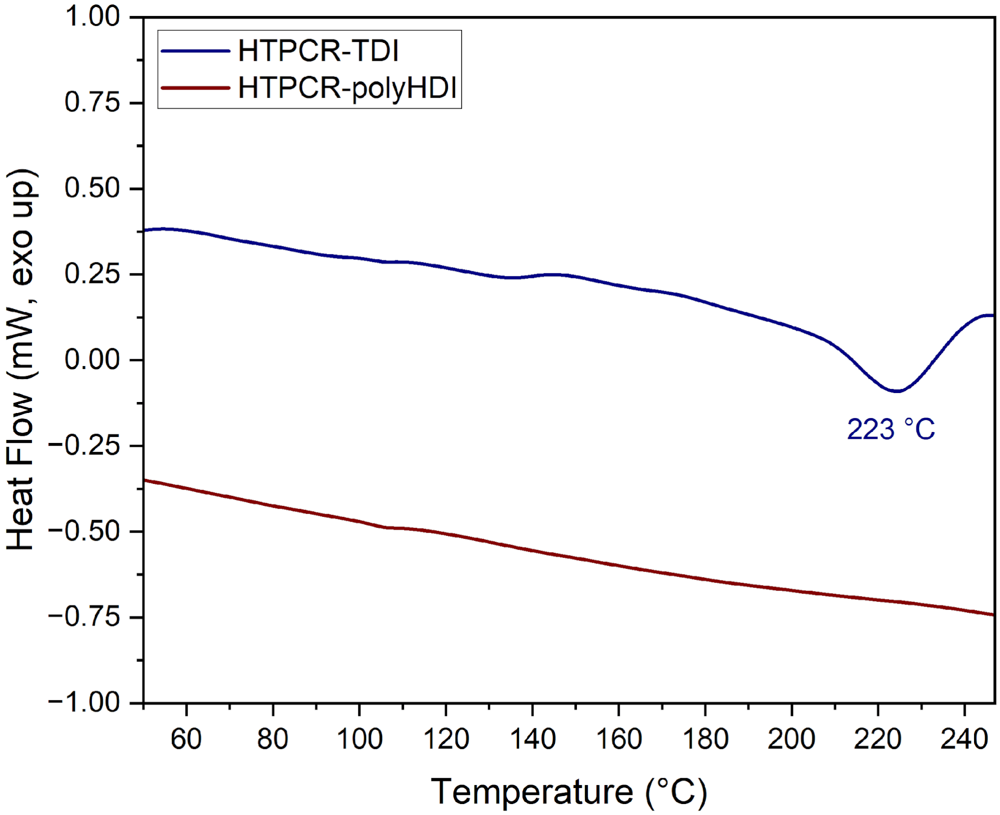

Figure S12A. DSC spectra of HTPCR-TDI and HTPCR-polyHDI elastomers indicating the melting event for HTPCR-TDI at 223 °C.

Because the adhesive PUs failed to form films amenable to evaluation of glass transition temperatures by DMA, the glass transition temperatures were evaluated by DSC. The annotated DSC spectra are given in Figure S13B.

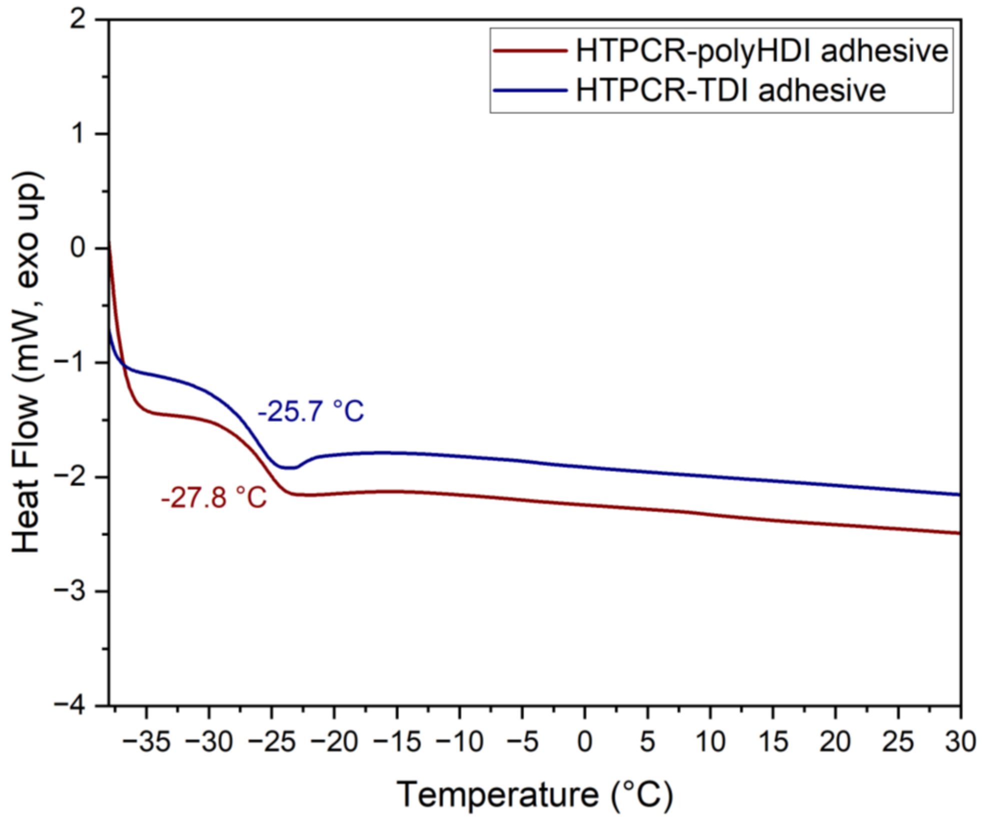

Figure S13B. DSC spectra of HTPCR-TDI and HTPCR-polyHDI adhesives used to determine the glass transition temperatures.

DMA Data for HTPCR-TDI and HTPCR-polyHDI Elastomers. The glass transition temperatures for HTPCR-TDI and HTPCR-polyHDI were identified by temperature-sweep DMA of the dogbones in tension mode, using the tan(δ) plot using parameters detailed in **Section I. General Methods**. The full DMA spectra are given in Figures S14A and Figure S14B. The DMA spectrum of HTPB-polyHDI is provided for comparison in Figure S14C.

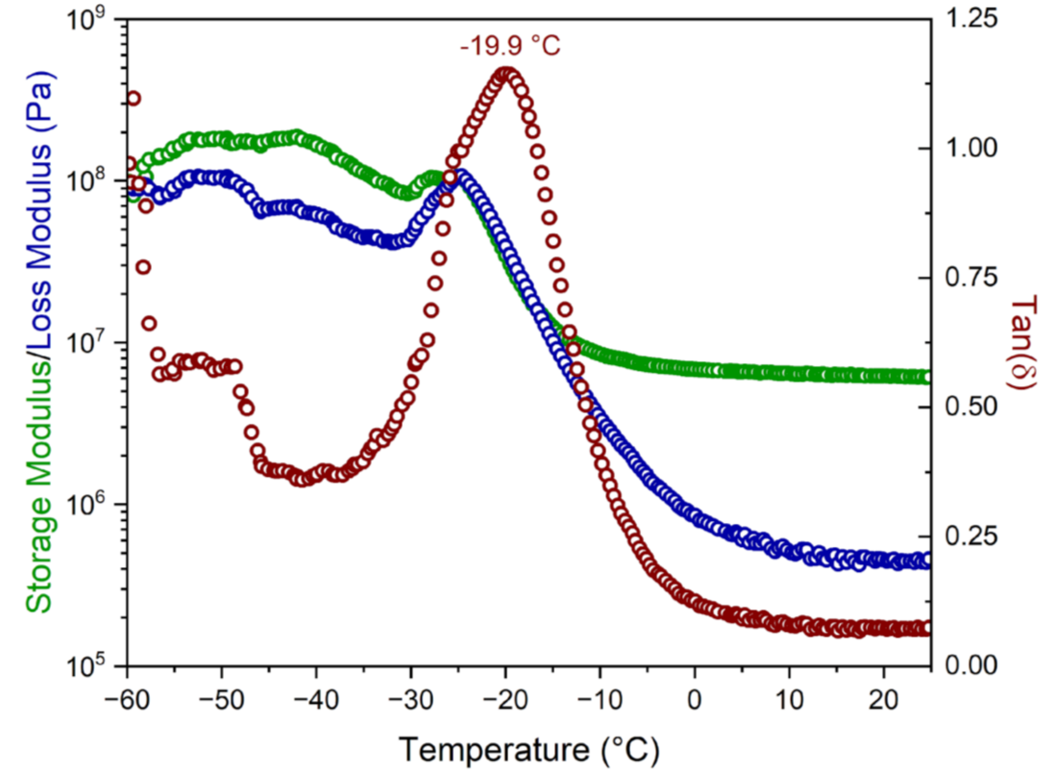

Figure S14A: Annotated temperature-sweep DMA plot of HTPCR-TDI.

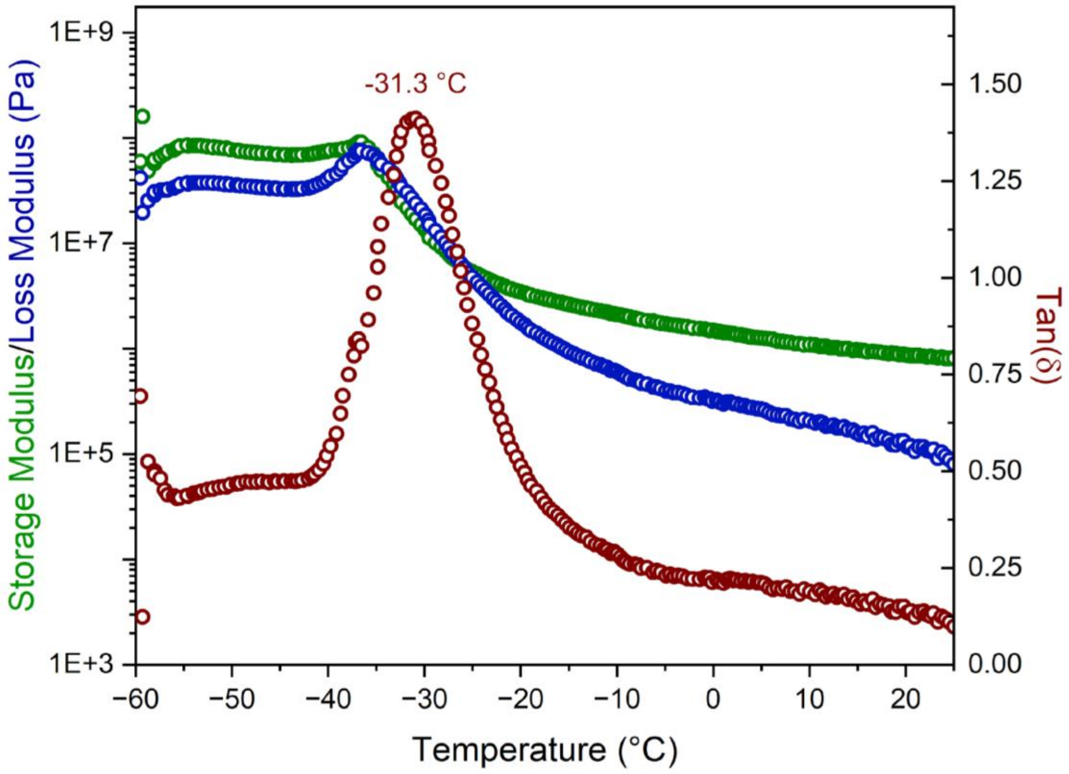

Figure S14B: Annotated temperature-sweep DMA plot of HTPCR-polyHDI.

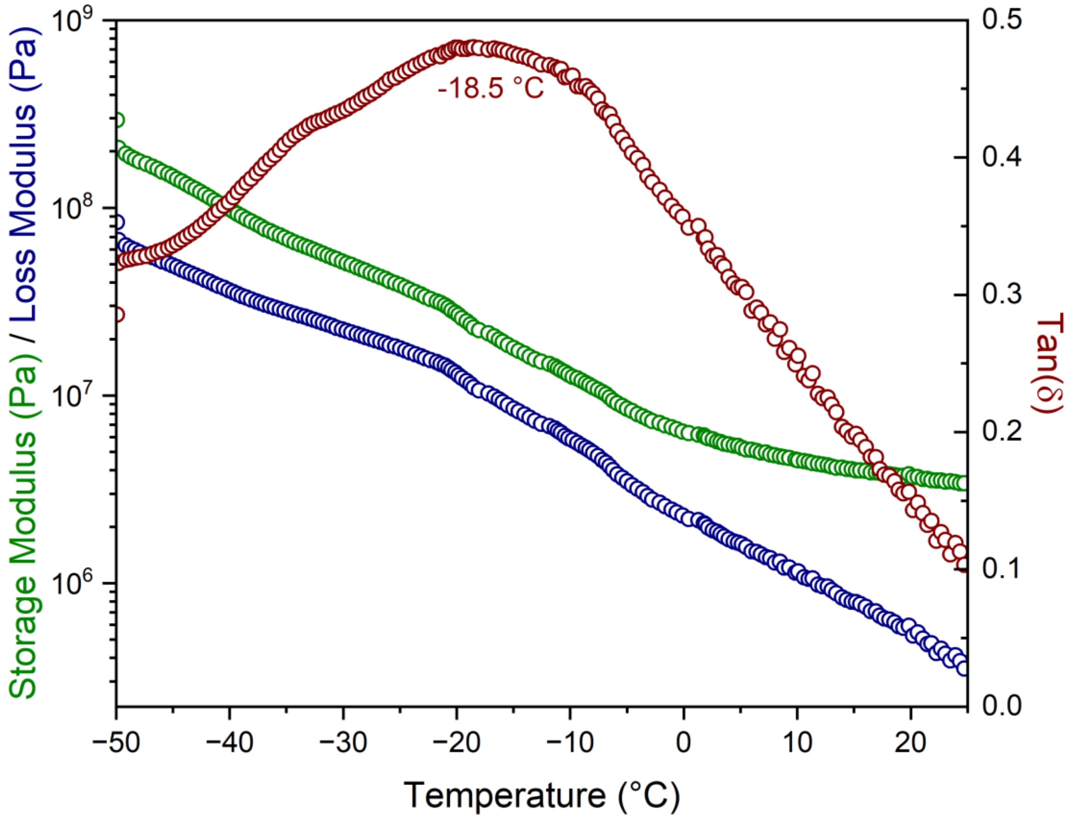

Figure S14C: Annotated temperature-sweep DMA plot of HTPB-polyHDI.

Lap Shear Testing of HTPCR-TDI and HTPCR-polyHDI Adhesives. The lap shear strength of the adhesives was tested by curing the PU adhesive formulations between two aluminum plates Two aluminum lap shear plates were roughened with 80 grit sandpaper. In a 20 mL scintillation vial in the glove box was added 0.25 g of HTPCR(1) (0.3125 mmol) and 2 mg (0.0156 mmol, 0.05 equiv.) of DBTDL. In a separate vial was added 1 equiv. of TDI or 0.67 equiv. of polyHDI and 1 mL of toluene. The polyisocyanate solutions were added to the polyol solutions, after which the contents were spread onto a ca. 20 x 20 mm section of each aluminum grip. Duplicates of each formulation were prepared. The grips were adhered to each other by weighting with a 30 g steel weight, after which the sealed grips were cured in an oven at 110 °C for 24 hours. The resulting thickness of the adhesives between the plates was 0.10-0.14 mm. A representative image of the resulting plates is given in Figure S15A. The plates were positioned on the tensile tester equipped with a 250 lbf force transducer, after which the position was adjusted 1.5 mm/min until the load was 400 N, sampling at 500 samples per second. The maximum load was then obtained from the load vs. position traces. Representative load vs. position traces are given in Figure S15B, and the tabulated data is given in Table S1.

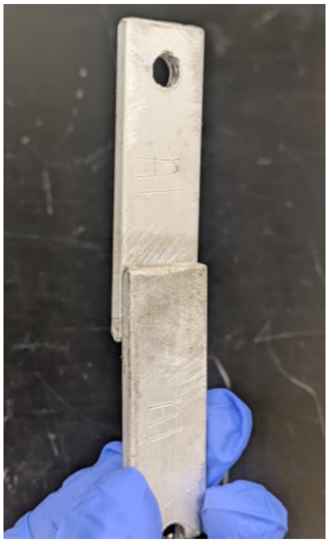

Figure S15A. Example adhered Al plates after adhesive curing.

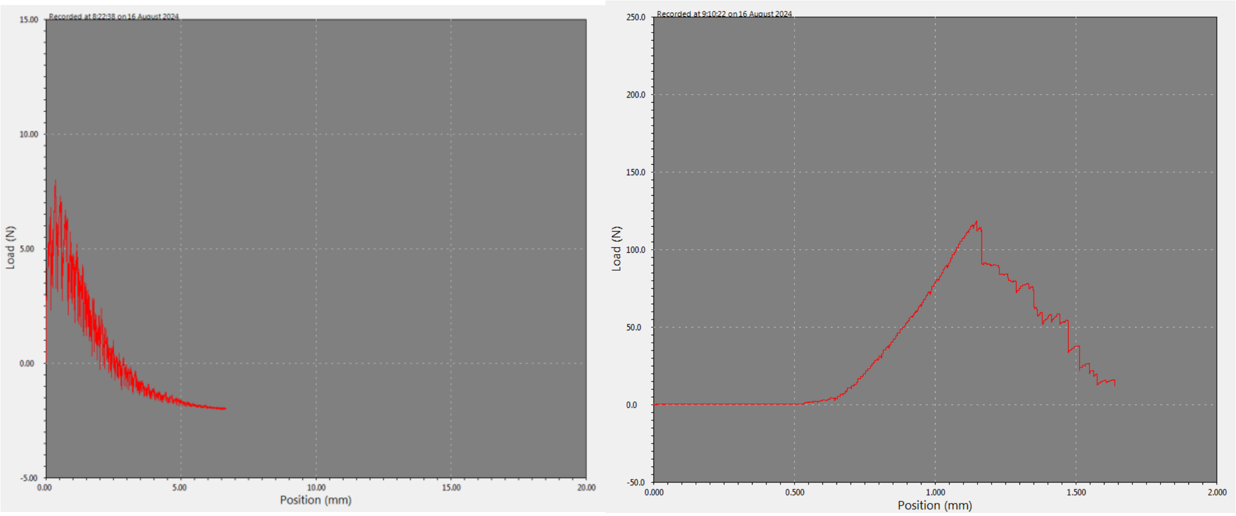

Figure S15B. Representative load vs. position curves obtained for HTPCR-TDI adhesive (left) and HTPCR-polyHDI adhesive (right).

Table S1. Tabulated Maximum Load Data for HTPCR-based PU Adhesives Synthesized.

	**Max load (N)**	**length (mm)**	**width (mm)**	**area (mm^2)**	**Max Load (kPa)**
HTPCR-TDI trial 1	25.79	20.01	19.64	393.00	65.63
HTPCR-TDI trial 2	13.80	20.01	19.64	393.00	35.11
average	19.80				50.37
standard deviation	6.00				15.26
HTPCR-polyHDI trial 1	70.28	20.35	19.40	394.79	178.03
HTPCR-polyHDI trial 2	118.34	24.46	19.44	475.50	248.88
average	94.31				213.45
standard deviation	24.03				35.43

Tensile Testing of HTPCR-TDI and HTPCR-polyHDI Elastomers. The elongation at break, load at break, and Young's Moduli of the polyurethane cures were determined by tensile testing using parameters detailed in **Section I. General Methods.** Both the thermoplastic and thermoset data were obtained in duplicate. The stress-strain curves are given in Figure S16, with the tabulated data from the curves given in Table S2. Results from HTPB-polyHDI are provided for comparison.

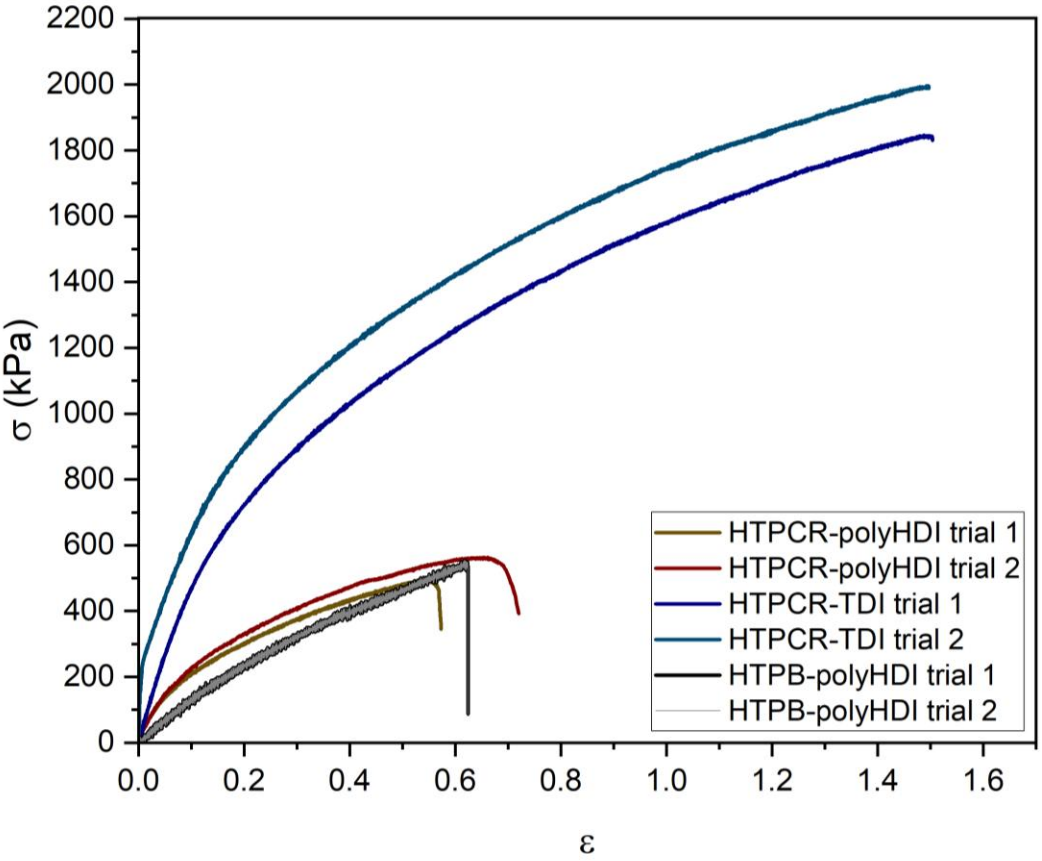

Figure S16. Stress-strain curves obtained from tensile testing of HTPCR-TDI and HTPCR-polyHDI PUs.

Table S2. Tabulated Young's Moduli, Elongation at Break, and Load at Break for HTPCR-TDI, HTPCR-polyHDI, and HTPB-polyHDI.

	Young's Modulus (MPa)	Elongation at Break (%)	Load at Break (MPa)
HTPCR-TDI Trial 1	4.548	150	1.839
HTPCR-TDI Trial 2	4.216	151	1.997
average	4.382	150	1.918
standard deviation	0.166	0.5	0.079
HTPCR-polyHDI Trial 1	3.368	72	0.552
HTPCR-polyHDI Trial 2	3.226	57	0.491
average	3.297	64.5	0.522
standard deviation	0.071	7.5	0.031
HTPB-polyHDI Trial 1	1.289	62.3	0.549
HTPB-polyHDI Trial 2	1.311	62.4	0.541
average	1.300	62.4	0.545
standard deviation	0.011	0.05	0.004

IV. Metathesis Activity of Thermoplastics and Thermosets.

General Procedure for Decomposition of Polymer Composites. To a vial in the glove box was added 200 mg of HTPCR-based PU shredded into approximately 10 mg pieces and a PTFE-coated stir bar. The shreds were suspended in 5 mL of dry THF. To the vial was then added 50 µL of a solution containing 20 mg of G2 in 0.5 mL of dry THF. The vial was sealed, removed from the glove box and stirred in a heating block at either ambient temperature or 50 °C for 48 hours. In successful decompositions, a color change from pink-red to golden brown was observed. At the 24-hour reaction interval, the vial was moved into the glove box, and a 1 mL aliquot of the reaction mixture was taken from the solubilized material for analysis by GPC. The vial was then capped under inert atmosphere and stirred for another 24 hours and another aliquot was removed for GPC analysis.

For determination of the percent decomposition of HTPCR-polyHDI, the insoluble material was separated from the solubilized decomposition products by pipetting, after which the insoluble material was dried under vacuum overnight and weighed. The percent decomposition was then calculated from the ratio of the mass of the recovered material to the initial mass of the thermoset subjected to decomposition.

Decomposition Catalyst Screen. To analyze the efficacy of commercially available metathesis catalysts in the decomposition of HTPCR-based PUs, a screen was conducted whereby HTPCR-TDI was depolymerized with 1 weight percent of either Grubbs 1st Generation (G1), Grubbs 2nd Generation (G2) or Grubbs 3rd Generation (G3) catalysts at room temperature over 48 hours. Reactions were set up as detailed in the **General Procedure for the Decomposition of Polymer Composites** section and analyzed every 24 hours by GPC. To prepare a GPC sample, a 1 mL aliquot of the reaction mixture was removed via syringe and filtered through a 0.22 µm pore size hydrophobic PTFE syringe filter into an autosampler vial. The results of the screen are given in Table S3, and the GPC chromatograms are given in Figure S17. Based on the results of the

screen, G2 appeared most effective in reducing the molecular weight of HTPCR-TDI under ambient conditions and was selected as the catalyst of choice for the remainder of the study.

Table S3. Tabulated Molecular Weight and Dispersity Data Obtained from the Catalyst Screening of the Decomposition of HTPCR-TDI.

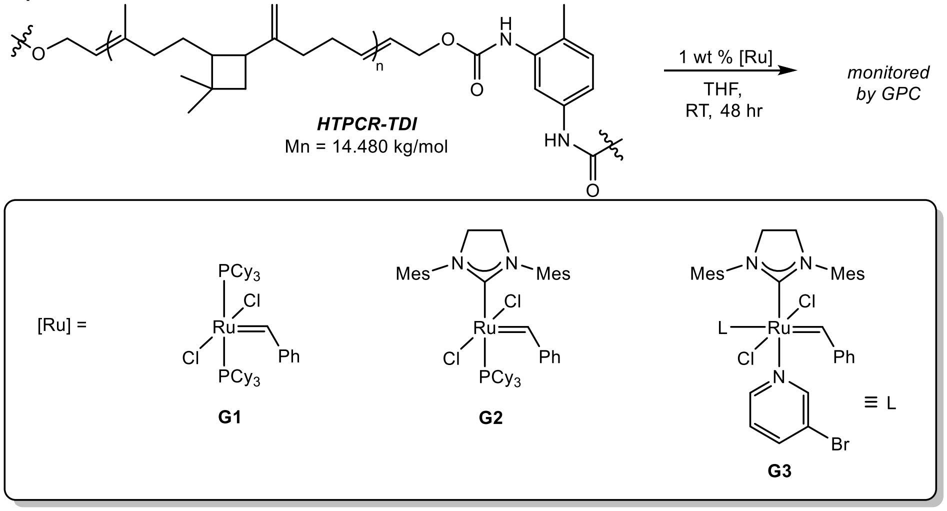

Catalyst	Time (hr)	M_n (kg/mol)	Đ
**G1**	24	14.436	7.6
	48	15.390	5.0
**G2**	24	5.543	3.1
	48	5.037	3.4
**G3**	24	8.911	6.7
	48	8.843	5.2

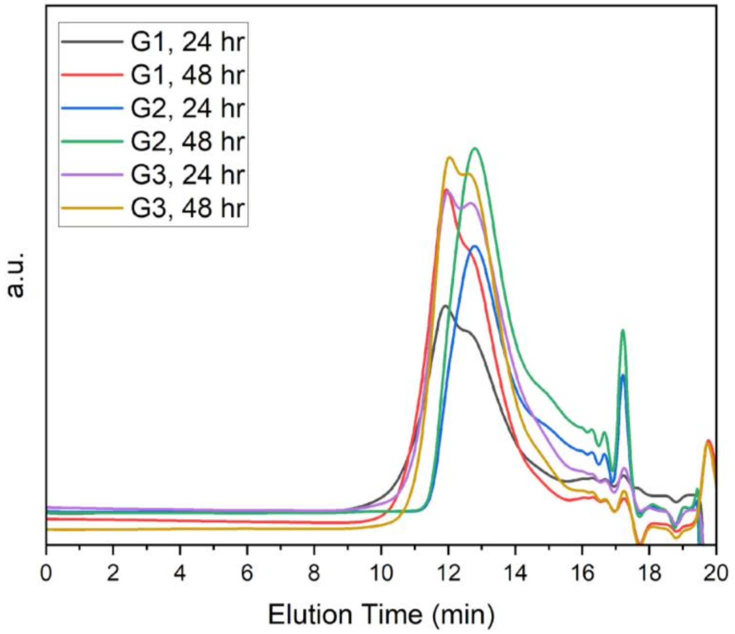

Figure S17. GPC chromatograms of the decomposition products of the catalyst screen.

Catalyst Loading Screen. To analyze the effect of catalyst loading on extent of decomposition, the catalyst loading was systematically reduced in the decomposition of HTPCR-TDI at room temperature for 48 hours, using the following general procedure: To a vial in the glove box was added 100 mg of HTPCR-TDI shredded into approximately 10 mg pieces and a PTFE-coated stir bar. The shreds were suspended in 2.5 mL of dry THF. To the vial was then added 10 μL (0.1 wt%), 50 μL (0.5 wt%), or 100 μL (1 wt%) of a solution containing 5 mg of G2 in 0.5 mL of dry THF. The vial was sealed, removed from the glove box and stirred in a heating block at ambient temperature for 48 hours. At the 48-hour reaction interval, the vial was moved into the glove box, and a 1 mL aliquot of the reaction mixture was taken from the solubilized material for analysis by GPC. The GPC data indicate significant reductions in extent of decomposition upon reduction of catalyst loading, as reported in Table S4. The GPC chromatograms for the catalyst screen are given in Figure S18.

Table S4. Tabulated Molecular Weight and Dispersity Data Obtained from the Catalyst Loading Screening of the Decomposition of HTPCR-TDI.

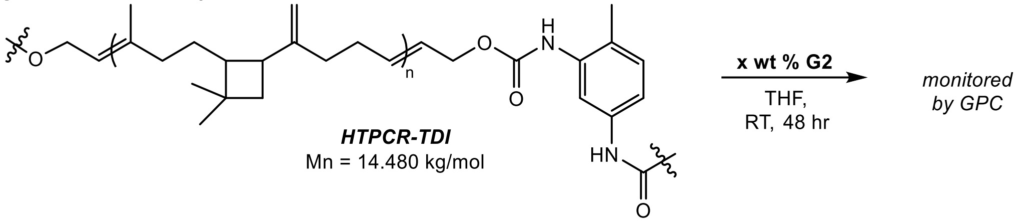

Weight %	M_n (kg/mol)	Đ
0.1	19.1	7.4
0.5	9.6	4.8
1	6.8	3.6

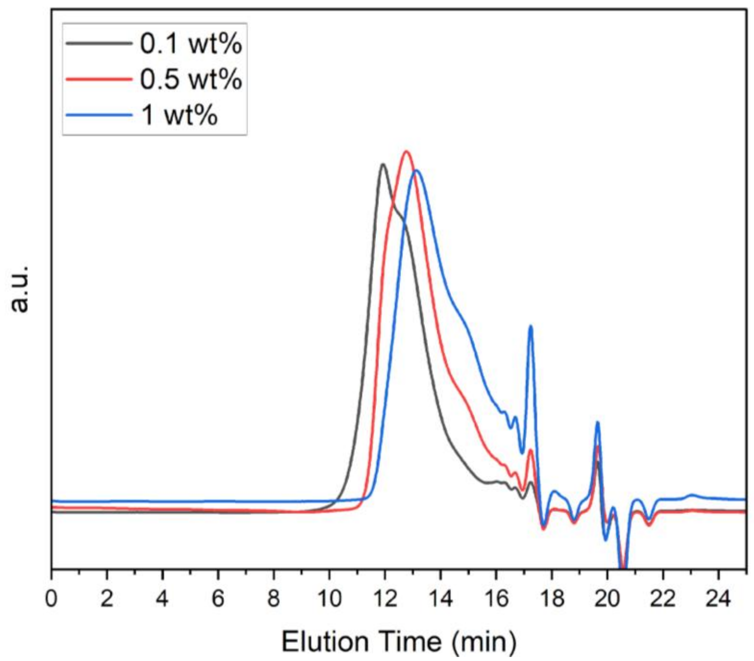

Figure S18. GPC chromatograms of the decomposition products of the loading screen.

GPC Data for PU Decomposition Conditions Screen. The extent of decomposition of HTPCR-TDI thermoplastic and HTPCR-polyHDI thermoset under different reaction conditions was determined by GPC, using parameters detailed in **Section I. General Methods**. To prepare a GPC sample, a 1 mL aliquot of the reaction mixture was removed via syringe and filtered through a 0.22 μm pore size hydrophobic PTFE syringe filter into an autosampler vial. The GPC chromatograms for the decomposition condition screening are given in Figure S19, and the tabulated molecular weight and dispersity data is given in Table 6 of the main text.

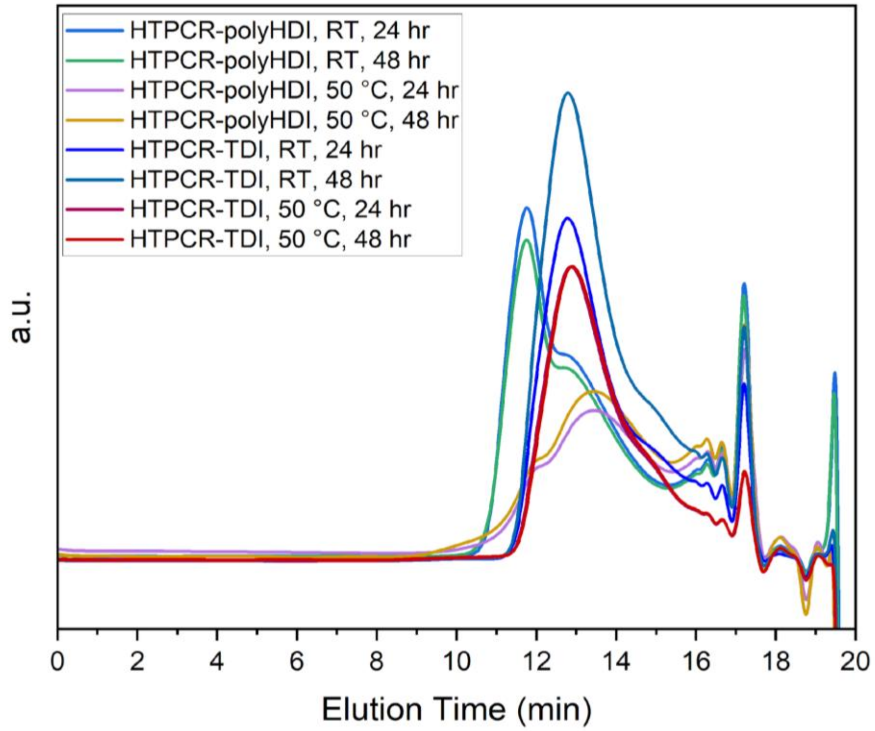

Figure S19. Combined GPC chromatograms obtained from the metathesis decomposition of HTPCR-TDI and HTPCR-polyHDI under varying reaction conditions.

Determination of Decomposition Products by NMR. The decomposition process for both HTPCR-TDI and HTPCR-polyHDI was monitored *in situ* by both ^{1}H and ^{13}C{^{1}H} NMR spectroscopy, using the following general procedure. To a J young tube in the glovebox was added 60 mg of shredded PU. To the J young tube was then added 750 μL of benzene-d_6, followed by 50 μL of a solution containing 20 mg of G2 in 0.5 mL of benzene-d_6. The J young tube was sealed and sonicated for 7 hours, ensuring the sonicator bath did not reach a temperature higher than 30 °C, with NMR spectra taken intermittently. The solutions turned from a pink to yellow/brown color over the course of the reaction, with near complete solubilization of the PU solids observed. Images of the tubes over a segment of the sonication process are given in Figure S20. The tube was then left to stand at ambient temperature overnight, with occasional agitation by inversion, in which a final NMR was taken after 24 hours of total reaction time. The J young tube contents were then analyzed by GPC and GC/MS for the identification of any small molecule cyclized products.

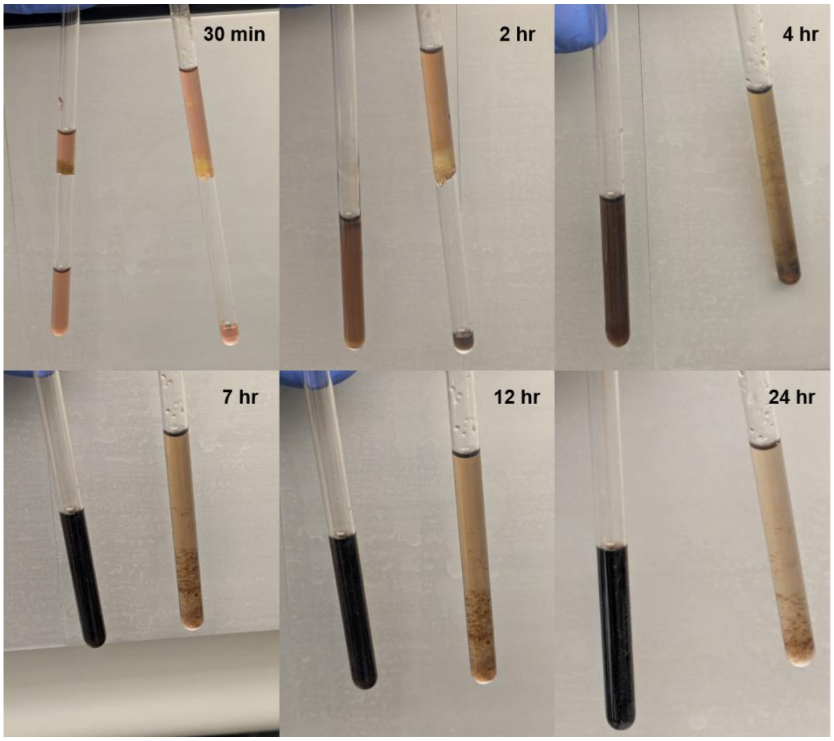

Figure S20. Images of the color changes and solubilization observed over the course of the reaction for HTPCR-TDI (left tube) and HTPCR-polyHDI (right tube).

Given the similarity between the final products of decomposition and the starting thermoplastic material, the ^{1}H NMR timecourse obtained did not reveal any significant change in features that could be used to assess product formation. However, monitoring the decomposition by $^{13}C\{^{1}H\}$ NMR permitted assignment of decomposition products. The timecourse obtained by $^{13}C\{^{1}H\}$ NMR is given in Figure S21A. Over the course of the decomposition process, new olefinic signals arising from terminal vinyl groups are formed, as well as new signals in the aliphatic region that are attributed to liberated chain ends of polycaryophyllene repeat units, as identified by Grau and Mecking.[2] Full chain end assignment is given in Figure S21B. Tracking the relative integrations of the olefinic signals to the cyclobutyl methyl groups in the $^{13}C\{^{1}H\}$ NMR spectrum as a function of time indicated that both signals associated with the vinylidene as well as the trisubstituted olefin (both E and Z isomers) decrease over time (Figure S21C). However, the signals associated with the secondary carbon of the terminal olefin group remains relatively constant, while the primary carbon of the free vinyl group increases in concentration. Given that conversion of the trisubstituted olefin would produce the signal at 125 ppm, this stagnation in peak intensity at 125 ppm suggests that cleavage is potentially occurring at the trisubstituted main chain olefin as well. Further, the decrease in signal intensity of the vinylidene gives evidence for the participation of the vinylidene groups as decomposition initiators.

Comparison of the depolymerized material to that of authentic β-caryophyllene monomer indicates that the obtained product is not recovered monomer from the reverse of polycaryophyllene polymerization (Figure S21D). Authentic ^{1}H and ^{13}C NMR spectra of β-caryophyllene monomer in benzene-d_6 are given in Figure S21E and S21F, respectively, for comparison. The GPC obtained of the material post-decomposition indicated M_n = 5.320 kg/mol and Đ = 2.1 for the products; the GPC chromatogram is given in Figure S21G.

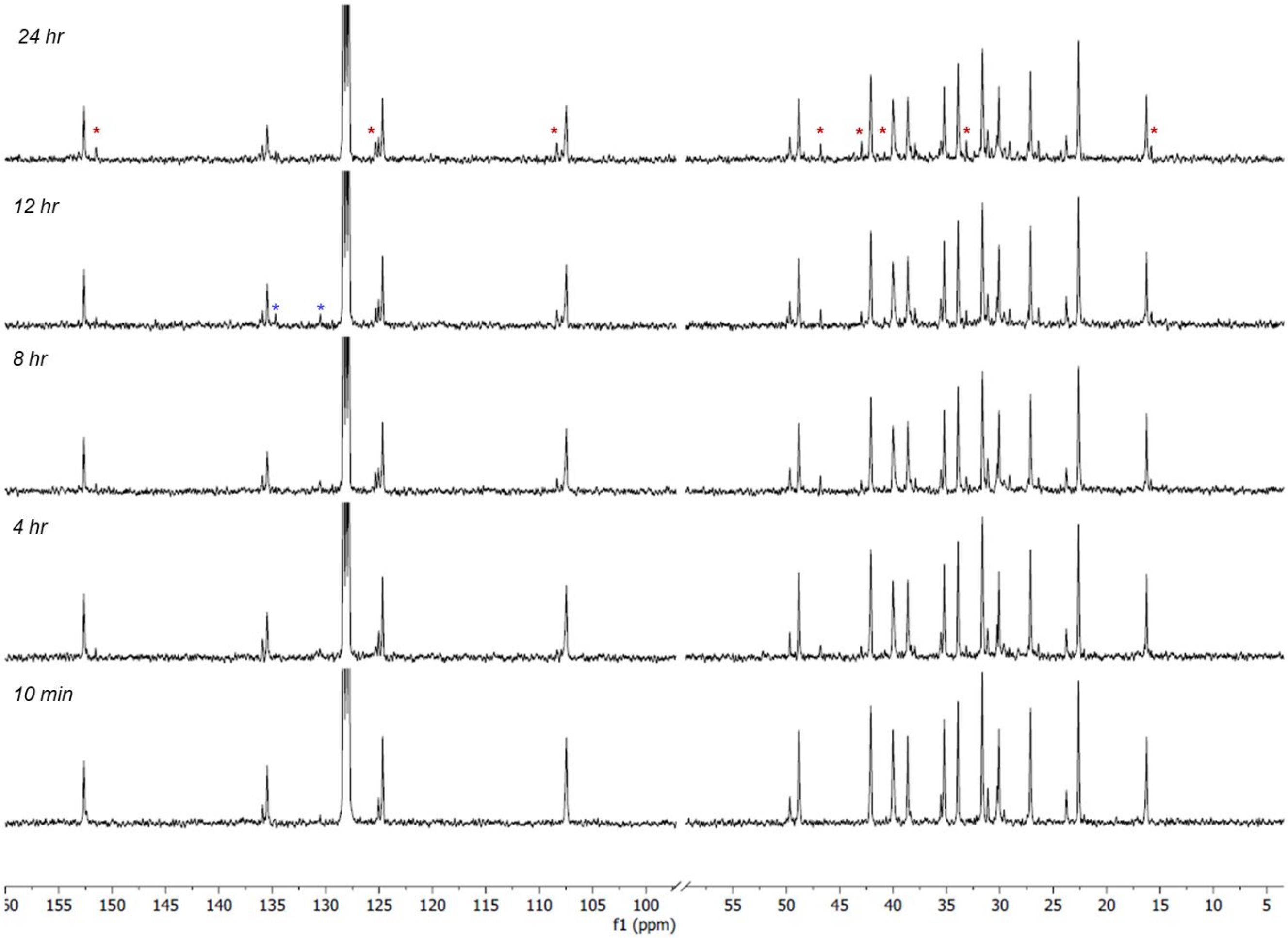

Figure S21A. $^{13}C\{^{1}H\}$ timecourse of the metathesis decomposition of HTPCR-TDI. New signals attributed to decomposition products are annotated with asterisks. Blue asterisks indicate vinyl signals attributable to the vinylated carbamate chain end.

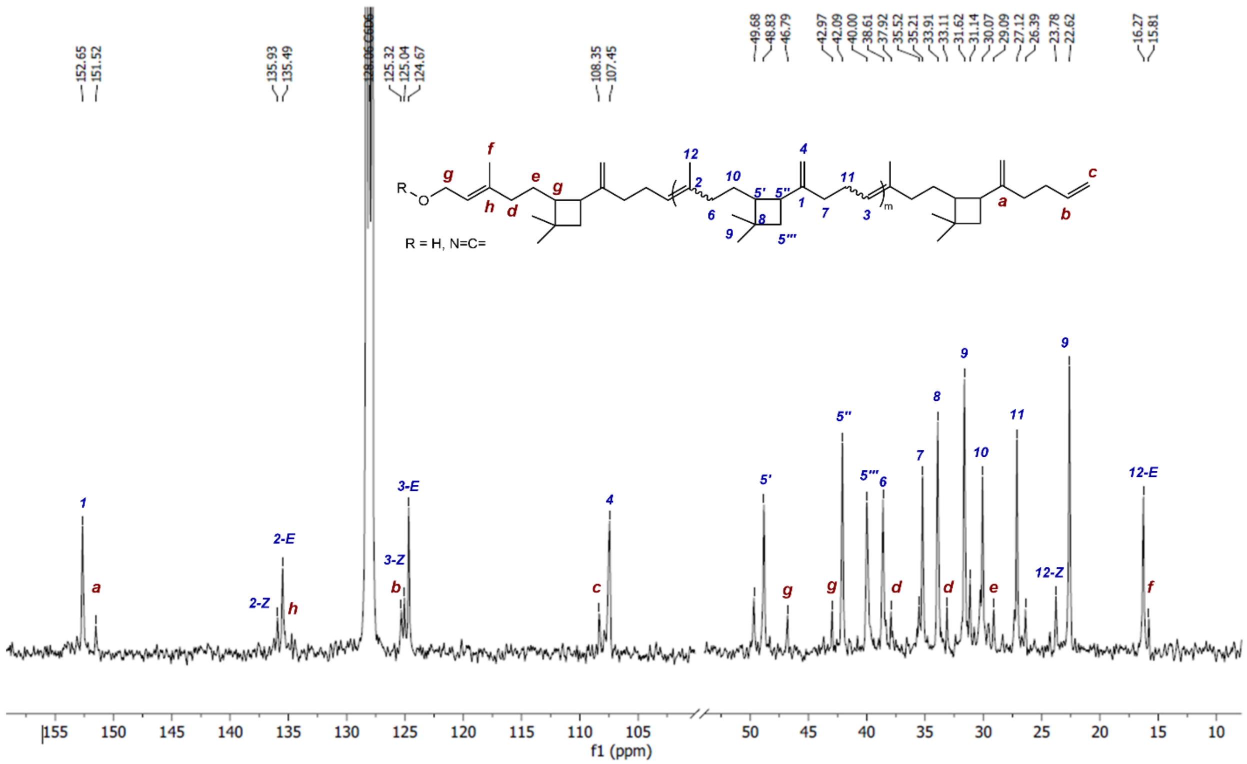

Figure S21B. Annotated $^{13}C\{^1H\}$ NMR spectrum of the chain ends of the products obtained from the decomposition of HTPCR-TDI.

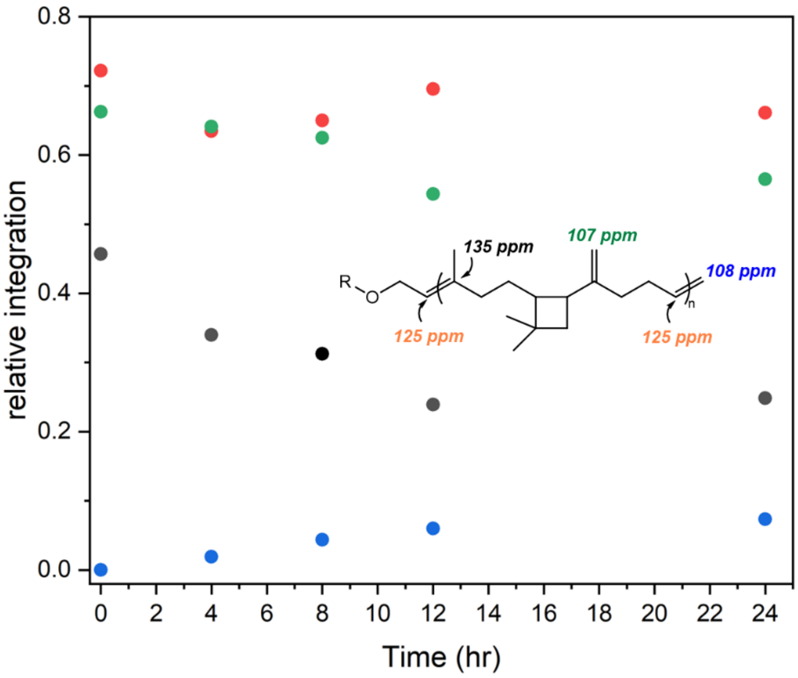

Figure S21C. Timecourse of the relative intensity of the olefin signals in the $^{13}C\{^{1}H\}$ NMR spectrum of the decomposition of HTPCR-TDI. Peak integrated relative to the cyclobutyl methyl signals.

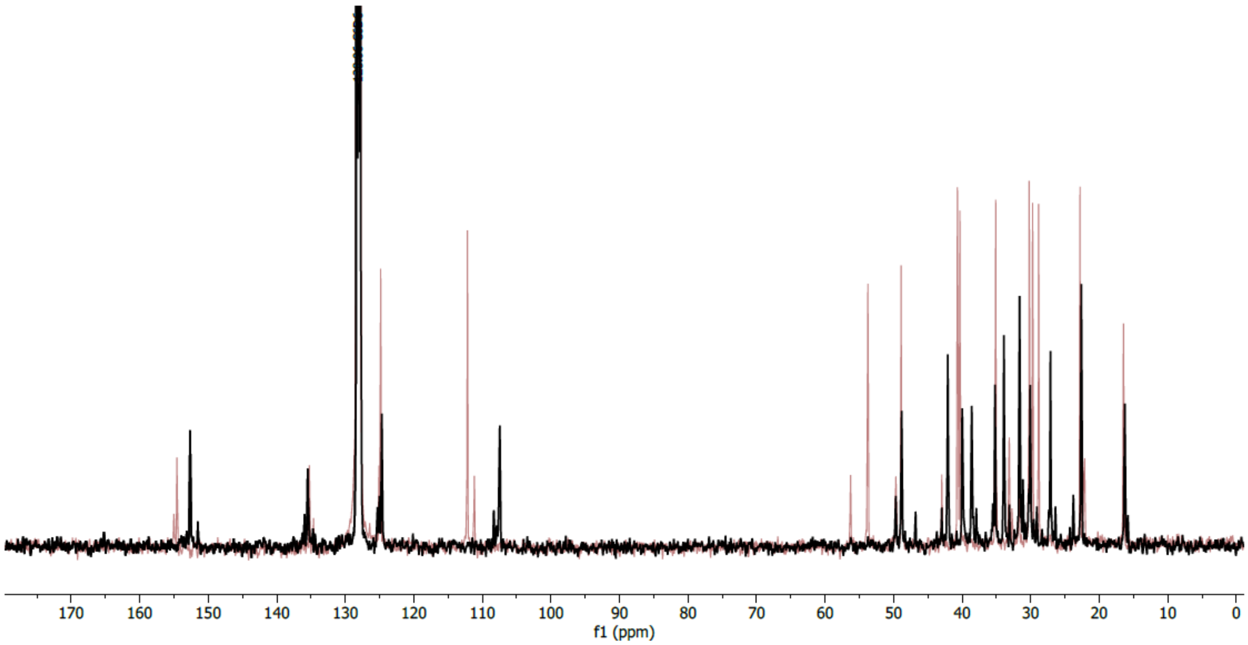

Figure S21D. Overlaid $^{13}C\{^{1}H\}$ NMR spectra of the products obtained from the decomposition of HTPCR-TDI (black) and authentic β-caryophyllene monomer (red).

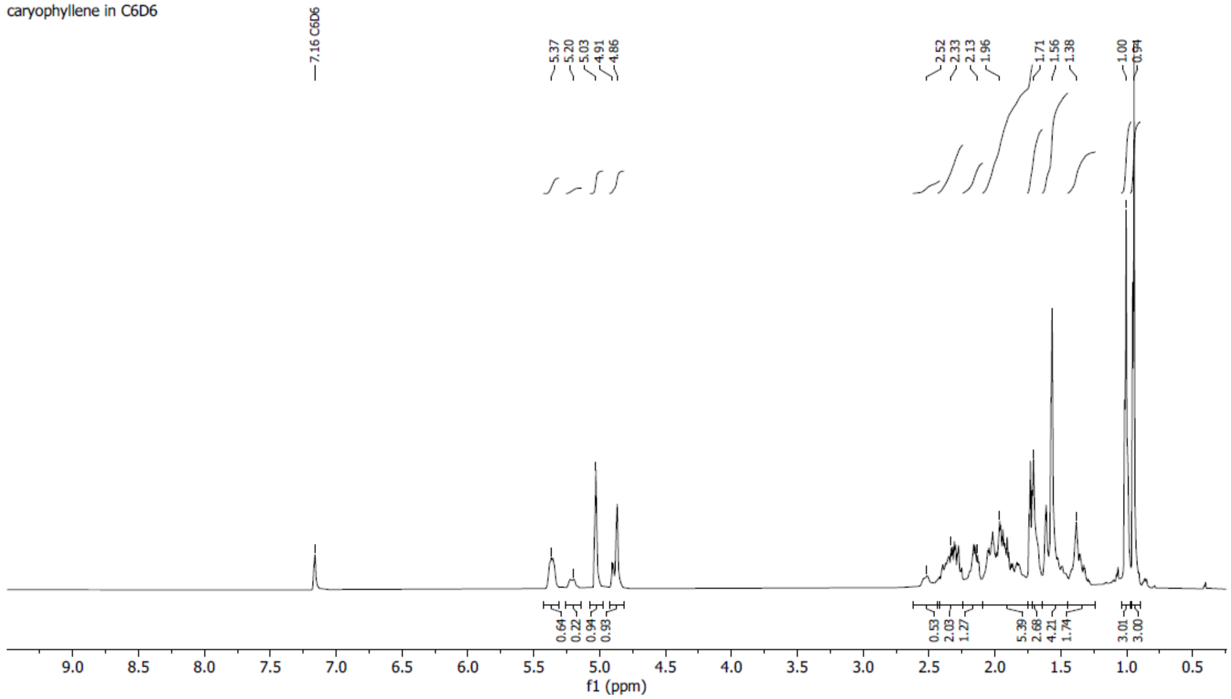

Figure S21E. ^{1}H NMR spectrum (benzene-d_6, 25 °C) of β-caryophyllene monomer.

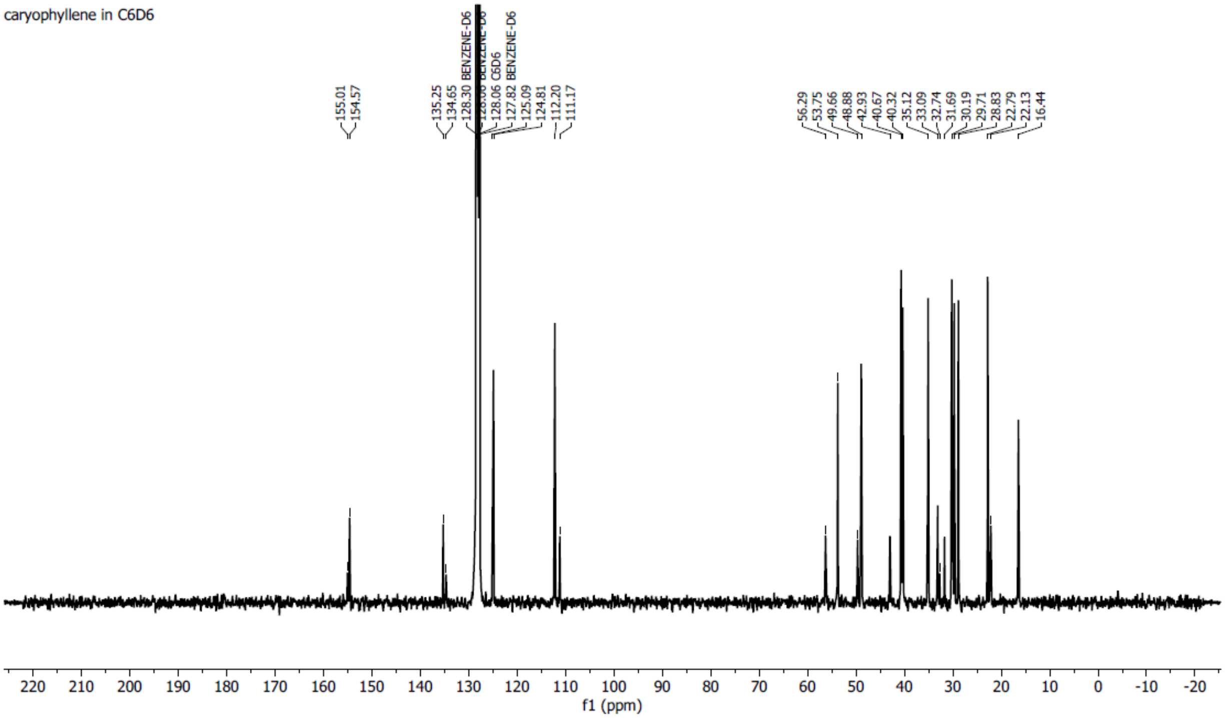

Figure S21F. $^{13}C\{^{1}H\}$ NMR spectrum (benzene-d_6, 25 °C) of β-caryophyllene monomer.

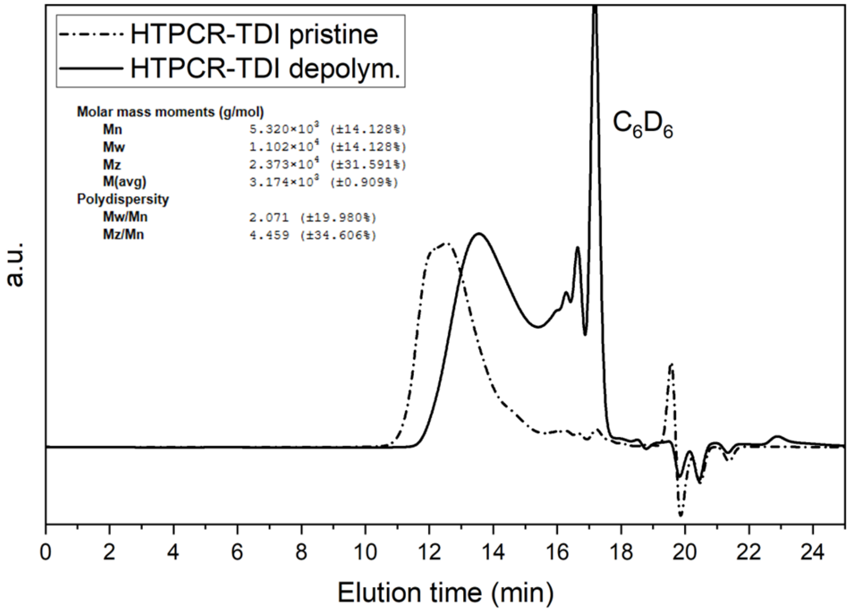

Figure S21G. GPC chromatogram of the products arising from the decomposition of HTPCR-TDI. The pristine HTPCR-TDI chromatogram is provided (dotted line) for comparison.

Monitoring the decomposition of HTPCR-polyHDI by $^{13}C\{^{1}H\}$ NMR once again produced the in-growth of a principally polycaryophyllene backbone along with the same chain ends identified

in Figure S21B for the decomposition of HTPCR-TDI. The $^{13}C\{^1H\}$ NMR timecourse for the decomposition of HTPCR-polyHDI is given in Figure S22A. Notably, the complete solubilization of the thermoset of the course of the reaction also provides evidence for chain scission. The GPC obtained of the material post-decomposition indicated M_n = 2.309 kg/mol and Đ = 2.4 for the products; the GPC chromatogram is given in Figure S22B.

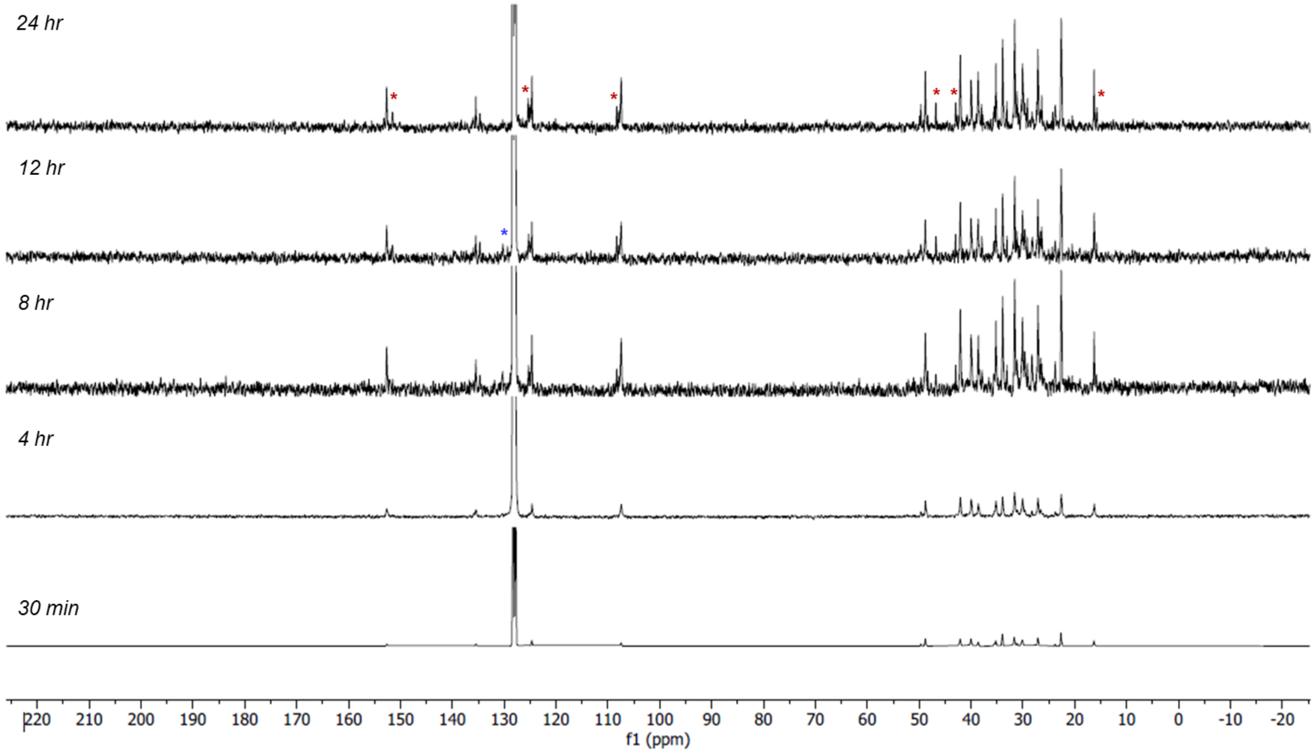

Figure S22A. $^{13}C\{^1H\}$ timecourse of the metathesis decomposition of HTPCR-polyHDI. New signals attributed to chain ends of decomposition products are annotated with asterisks, and blue asterisks denote peaks associated with the vinylated carbamate chain end.

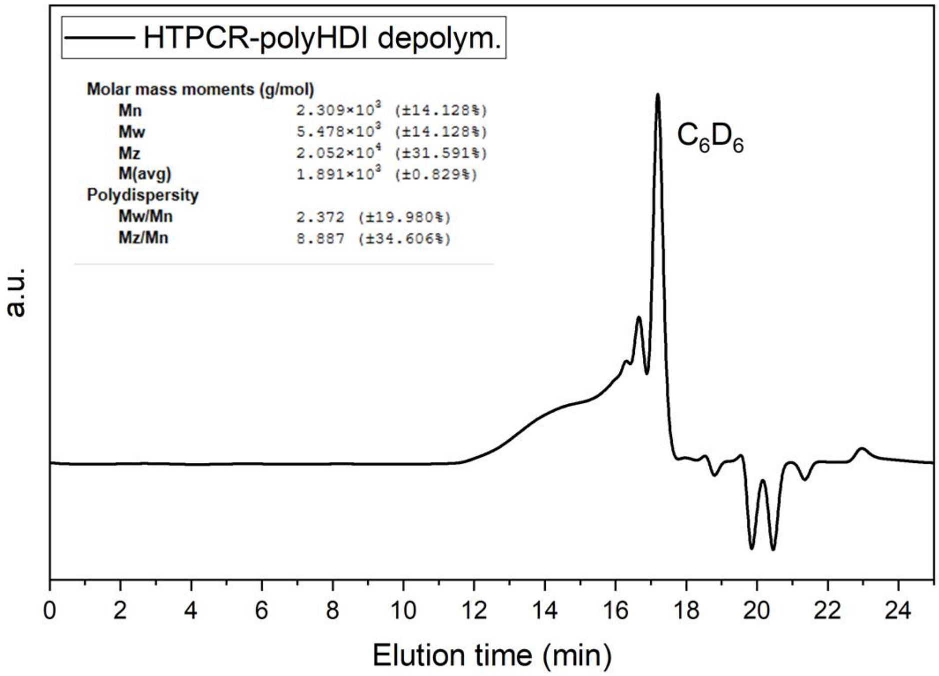

Figure S22B. GPC chromatogram of the products arising from the decomposition of HTPCR-polyHDI.

Determination of Decomposition Products by GC/MS. In order to identify any potential small molecule products arising from ring closure pathways, the soluble products from NMR decomposition of HTPCR-TDI and HTPCR-polyHDI were analyzed by GC/MS. The J young tubes were unsealed, after which the contents were poured into a vial, diluted with ca. 1 mL of dichloromethane, and filtered through a 0.22 μm pore size hydrophobic PTFE syringe filter into an autosampler vial. No small cyclized molecules were detected in the chromatograms, as shown in Figure S23. The chromatogram of authentic β-caryophyllene is provided for comparison.

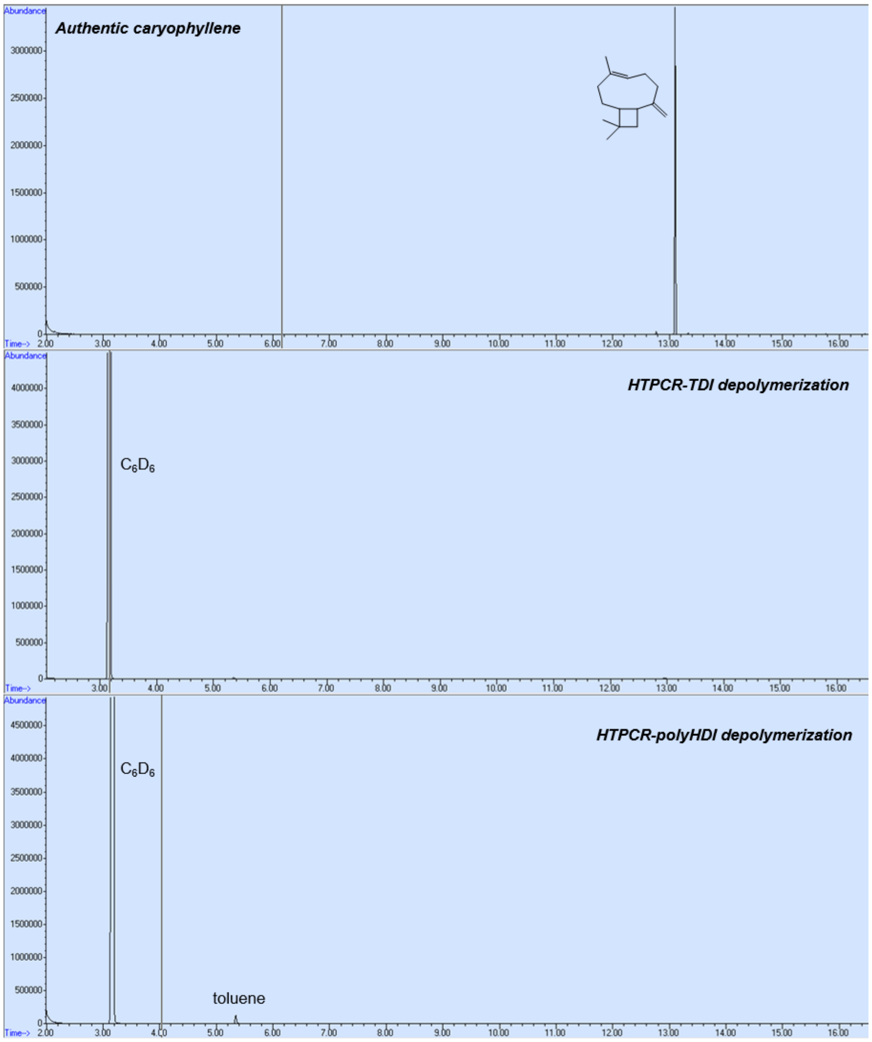

Figure S23. Comparative annotated gas chromatograms of β-caryophyllene (top) and the soluble products arising from the NMR-scale decomposition of HTPCR-TDI (middle) and HTPCR-polyHDI (bottom).

Control Experiments. A control experiment was run to assess any degradation of HTPCR-polyHDI in the absence of G2. To a 20 mL scintillation vial in the glove box was added a 59.9 mg sample of HTPCR-polyHDI that was shredded into approximately 10 mg pieces along with a PTFE-coated stir bar. 5 mL of THF was added, and the vial was sealed and stirred at ambient temperature for 48 hours. After 48 hours, the THF was decanted from the solids, and the solids were evaporated to dryness under vacuum and weighed. The mass of recovered material was found to be 60.2 mg (>99% recovery).

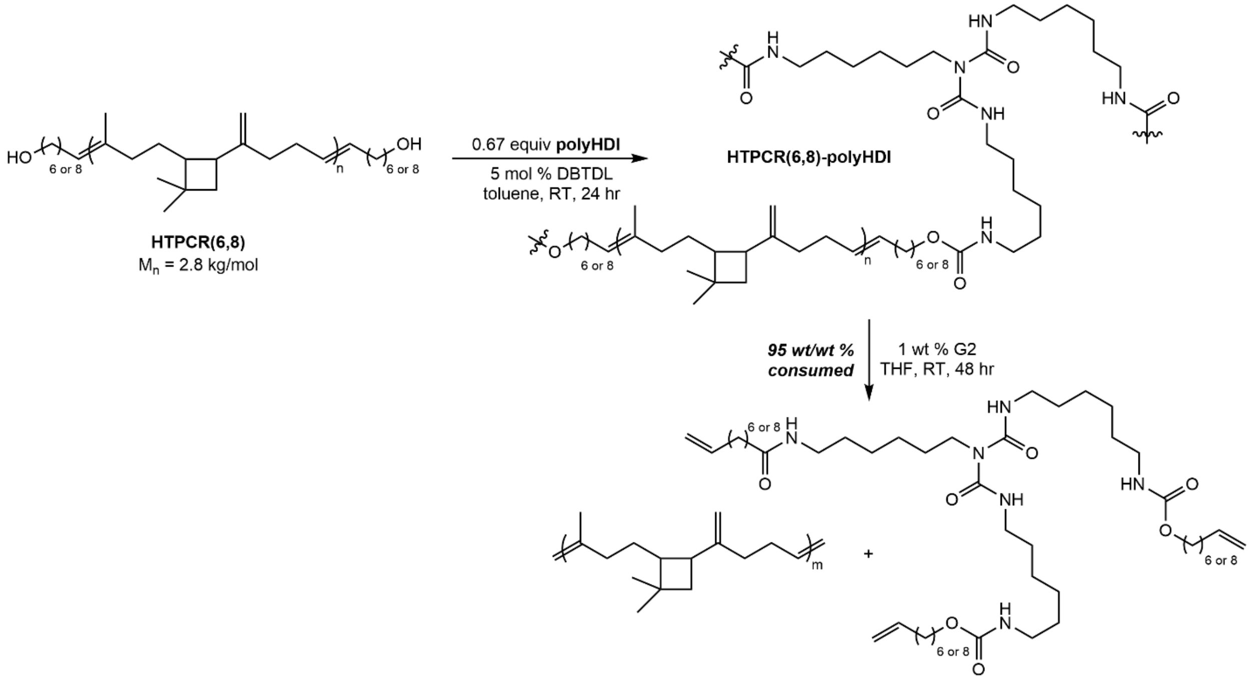

Synthesis and NMR Decomposition of HTPCR(6,8)-based PU Thermoset. For comparison of decomposition products to that of PUs made from HTPCR(1), a thermoset PU composed of HTPCR(6,8) polyol was synthesized in the following procedure. To a vial in the glove box was added 600 mg (0.214 mmol) of HTPCR(6,8) (M_n = 2.8 kg/mol) and DBTDL (4.4 mg, 0.00716 mmol, 0.05 equiv.). In a separate vial was added 68 mg (0.143 mmol, 0.67 equiv.) of polyHDI and 1 mL of THF. The vials were brought out of the glovebox, after which the THF solution was poured into the HTPCR(6,8) vial and mixed until dissolution of the contents. The homogenized mixture was then poured into a silicone dogbone mold (dimensions 60 mm x 10 mm x 4 mm, for more information see ref. 3) and left to cure at ambient temperature for 24 hours. The resulting thermoset was then characterized by ATR-IR. The ATR-IR spectrum of the thermoset is given in Figure S24.

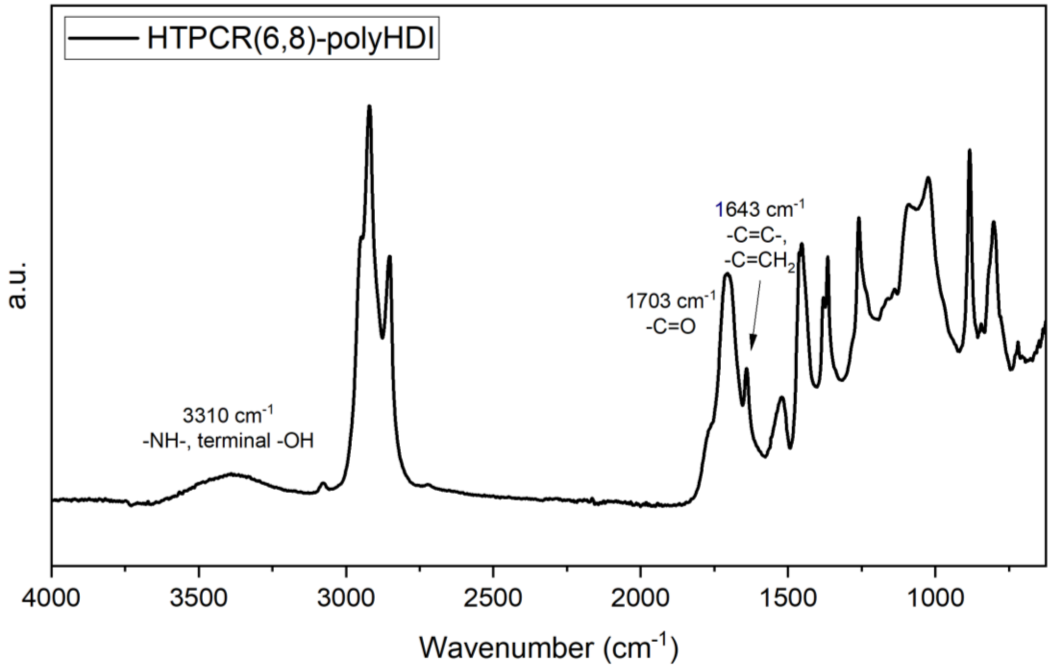

Figure S24. ATR-IR spectrum confirming the synthesis of PU thermoset HTPCR(6,8)-polyHDI derived from HTPCR(6,8) polyol.

Decomposition of this material was conducted as detailed in **Determination of Decomposition Products by NMR**. The $^{13}C\{^1H\}$ NMR decomposition timecourse is given in Figure S25A, in which the in-growth of a principally polycaryophyllene product is evident, along with vinyl chain ends consistent with that previously seen in the decomposition of HTPCR-TDI and HTPCR-polyHDI. Comparison of the spectra obtained from this decomposition to that of HTPCR-TDI and HTPCR-polyHDI is given in Figure S25B, in which the same product patterns are identified. This implies that polycaryophyllene units are clipped off in the metathesis decomposition process.

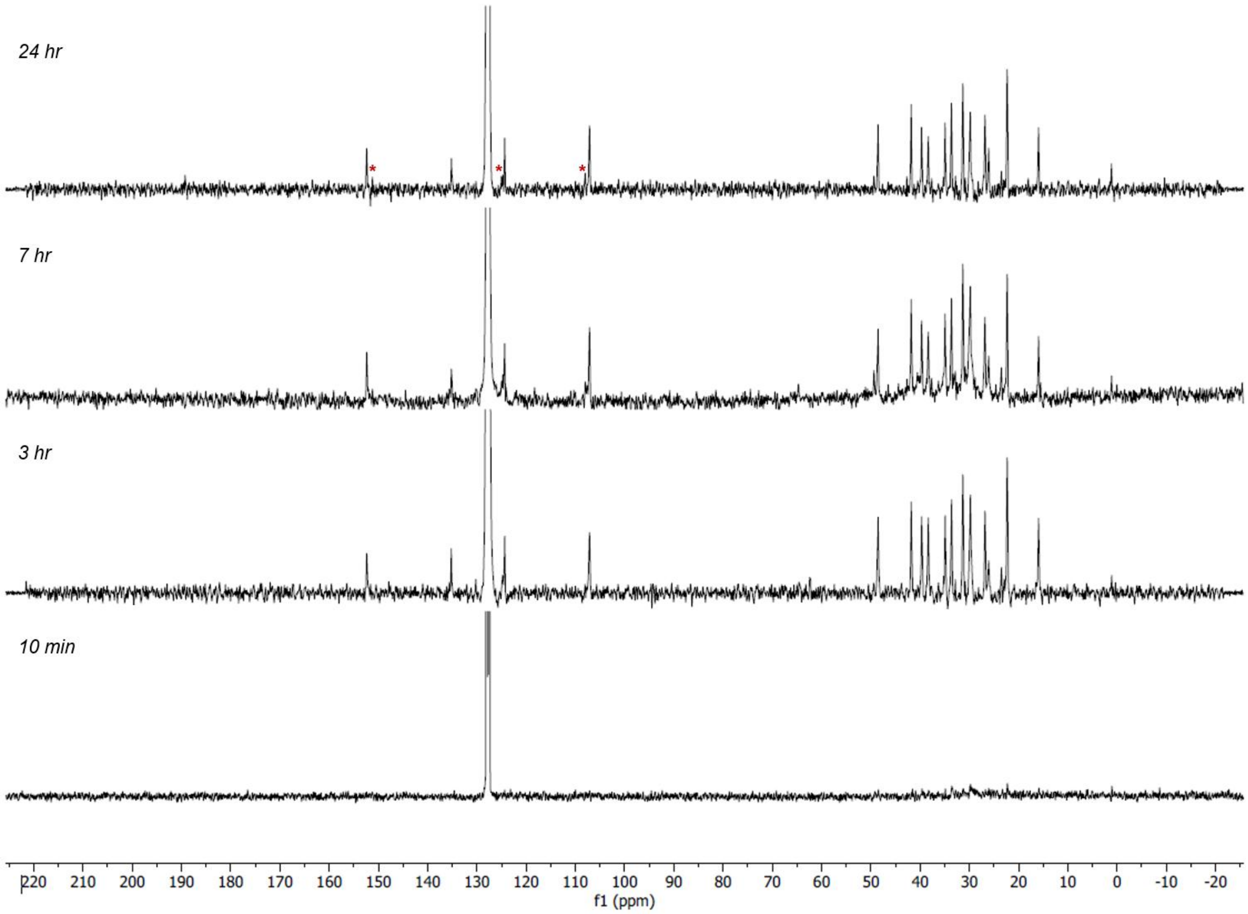

Figure S25A. $^{13}C\{^{1}H\}$ timecourse of the metathesis decomposition of thermoset PU derived from HTPCR(6,8). New signals attributed to chain ends of decomposition products are annotated with asterisks.

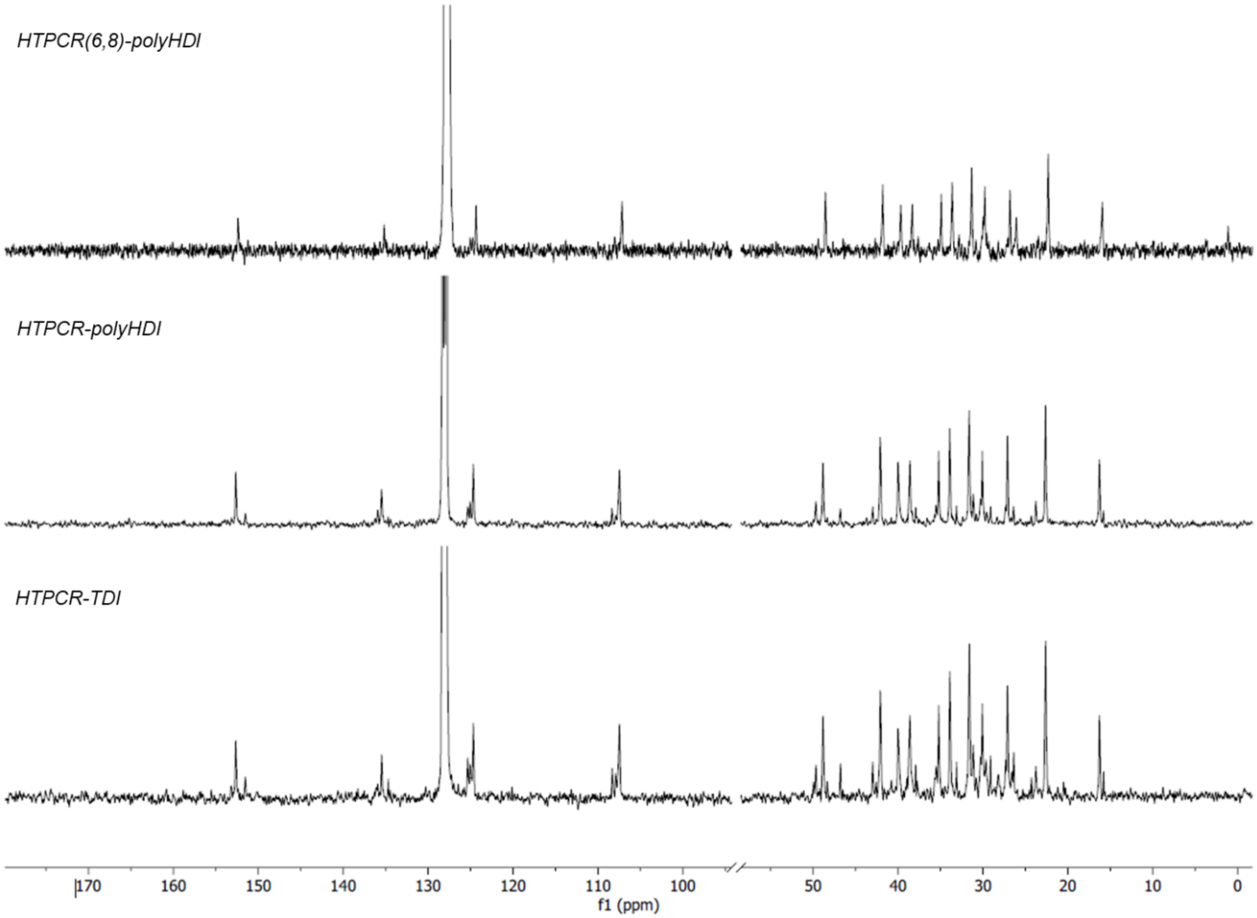

Figure S25B. Stacked $^{13}C\{^{1}H\}$ spectra of the products arising from the decomposition of HTPCR(1)-based PU thermoplastics and thermosets and HTPCR(6,8) based thermosets, indicating the same polycaryophyllene product formed.

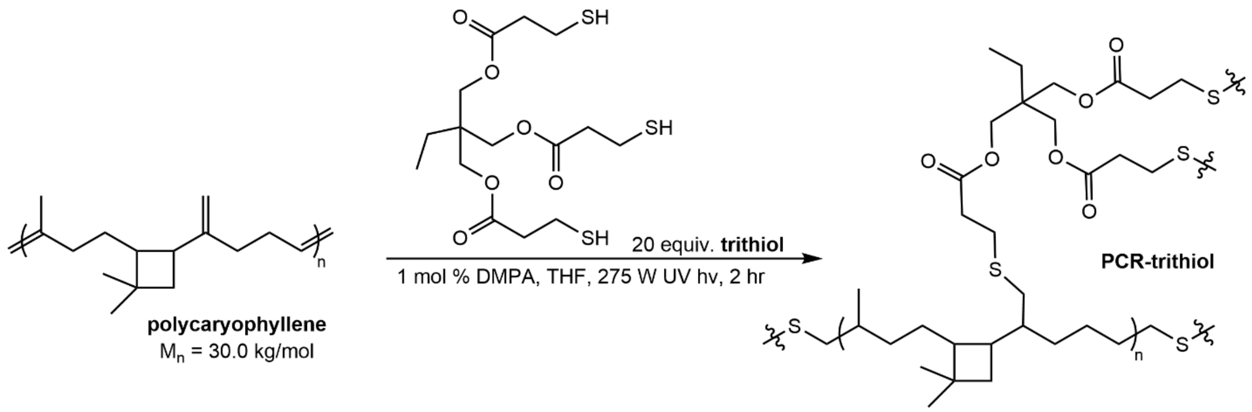

Synthesis of PCR-trithiol Thermoset. To eliminate all vinylidene intramolecular chain terminators, PCR-trithiol was synthesized through established thiol-ene click chemistry[3] in the following procedure: To a vial in the glove box was added 600 mg of polycaryophyllene (M_n = 30.050 kg/mol, 0.0200 mmol), 160 mg (0.402 mmol, 20 equiv.) of trimethylolpropane tris(3-mercaptopropionate), and 1.5 mg (0.00600 mmol, 0.3 equiv.) of DMPA. The mixture was dissolved in 1 mL of THF, after which the vial was sealed, brought out of the glove box, and poured into a 2.5 inch aluminum pan. The pan was positioned approximately 10 inches away from a 275 W UV Sylvania Sunlamp and irradiated for 2 hours. After two hours, the solidified thermoset was removed from the pan and sonicated in acetone for 30 minutes to remove excess thiol. The formation of the thermoset and elimination of olefin content was confirmed by ATR-IR. The ATR-IR spectrum is given in Figure S26. The material was then subjected to decomposition conditions as detailed in the **General Procedure for Decomposition of Polymer Composites** section, recovering 95% of starting thermoset.

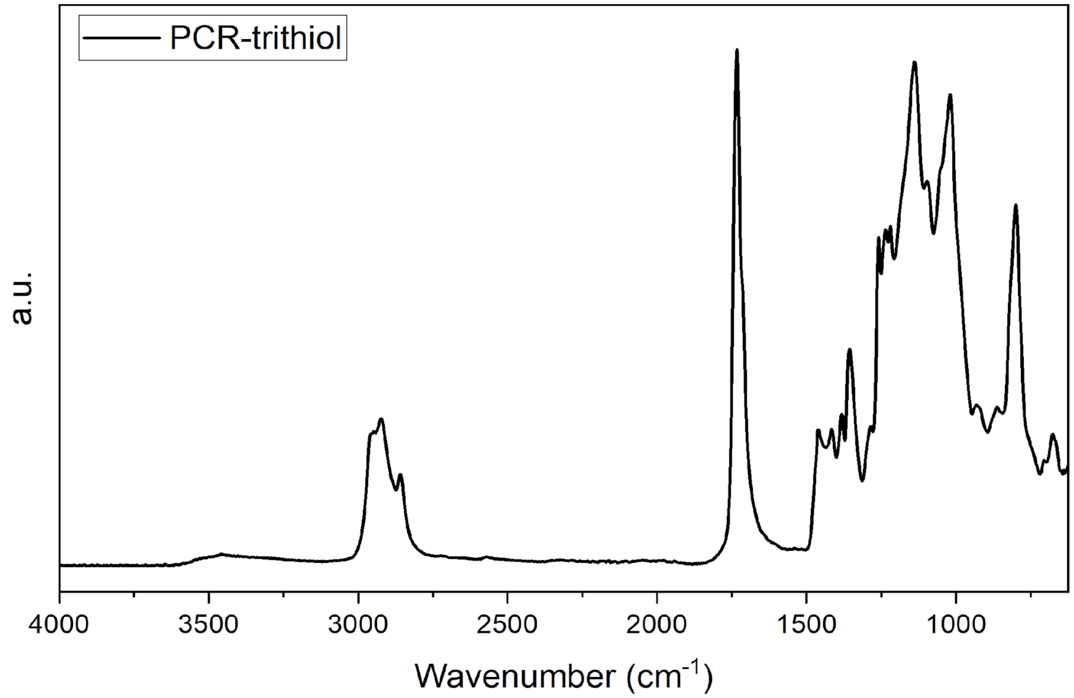

Figure S26. ATR-IR spectrum of PCR-trithiol.

Crosslinking of Decomposition Products. To demonstrate the utility of the thermoplastic material obtained from the metathesis decomposition process, the soluble organic fractions were isolated from a decomposition of HTPCR-polyHDI elastomer. To a 20 mL scintillation vial in the glove box was added 1.1663 g of HTPCR-polyHDI elastomer, 12 mg (1 wt %) G2, and a stir bar. The contents were dissolved/suspended in 10 mL of THF, and the vial was capped and stirred at room temperature for 48 hours. After 48 hours, the solubilized material was separated from the insoluble material by filtration, after which the soluble material was purified by column chromatography (silica solid phase, 100% EtOAc eluent) to recover 0.2637 g (23%) of a waxy thermoplastic material that was soluble in nonpolar and slightly polar organic solvents. The recovered material was characterized by ^{1}H, ^{13}C{^{1}H], and IR spectroscopies as well as GPC. The ^{1}H and ^{13}C NMR spectra are given in Figure S27A and S27B, the annotated IR spectrum is given in Figure S27C, and the GPC chromatogram is given in Figure S27D.

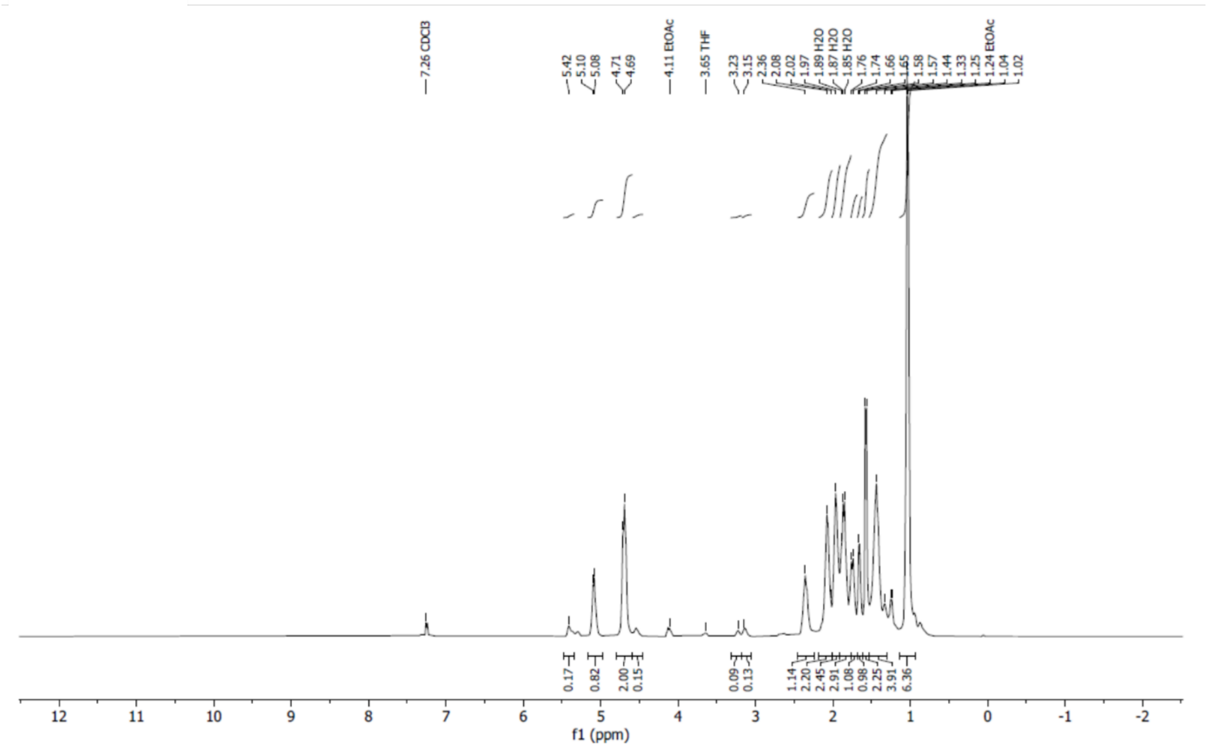

Figure S27A. ^{1}H NMR spectrum (benzene-d_6, 25 °C) of recovered thermoplastic material (M_n = 4.883 kg/mol, Đ = 4.0).

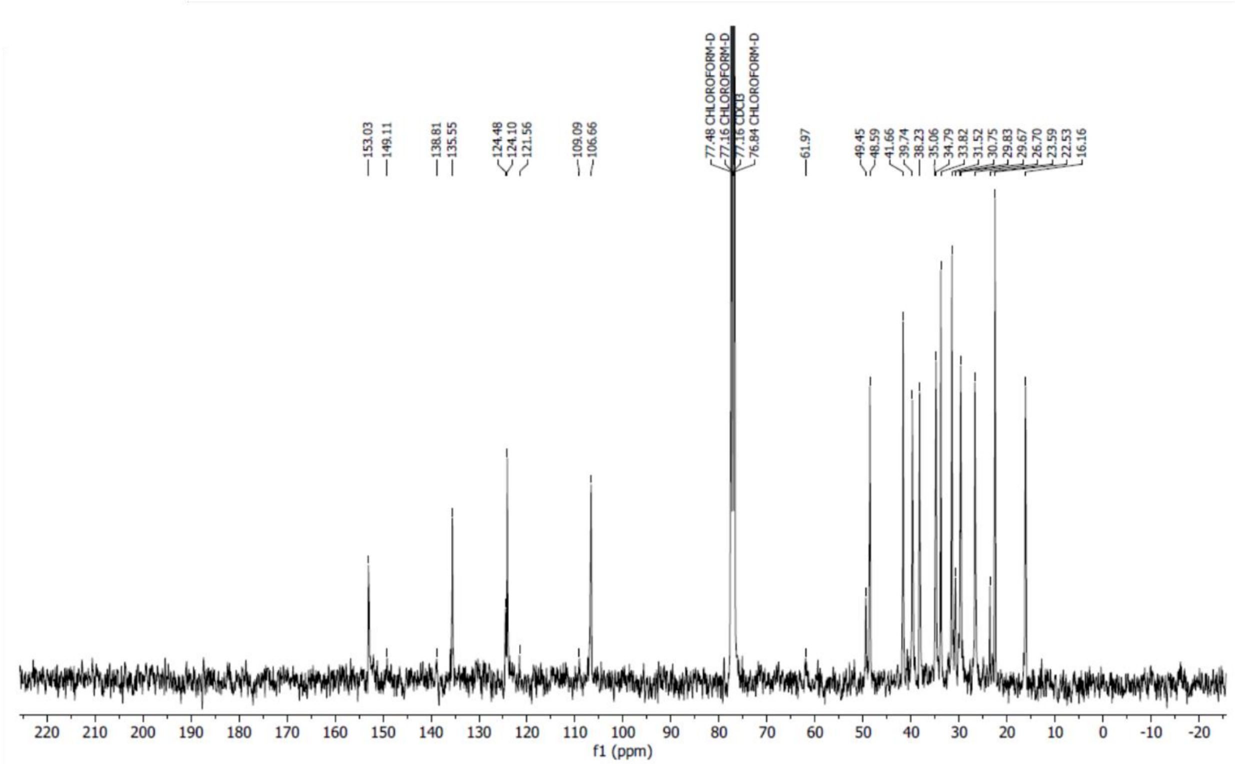

Figure S27B. $^{13}C\{^{1}H\}$ NMR spectrum (benzene-d_6, 25 °C) of recovered thermoplastic material (M_n = 4.883 kg/mol, Đ = 4.0).

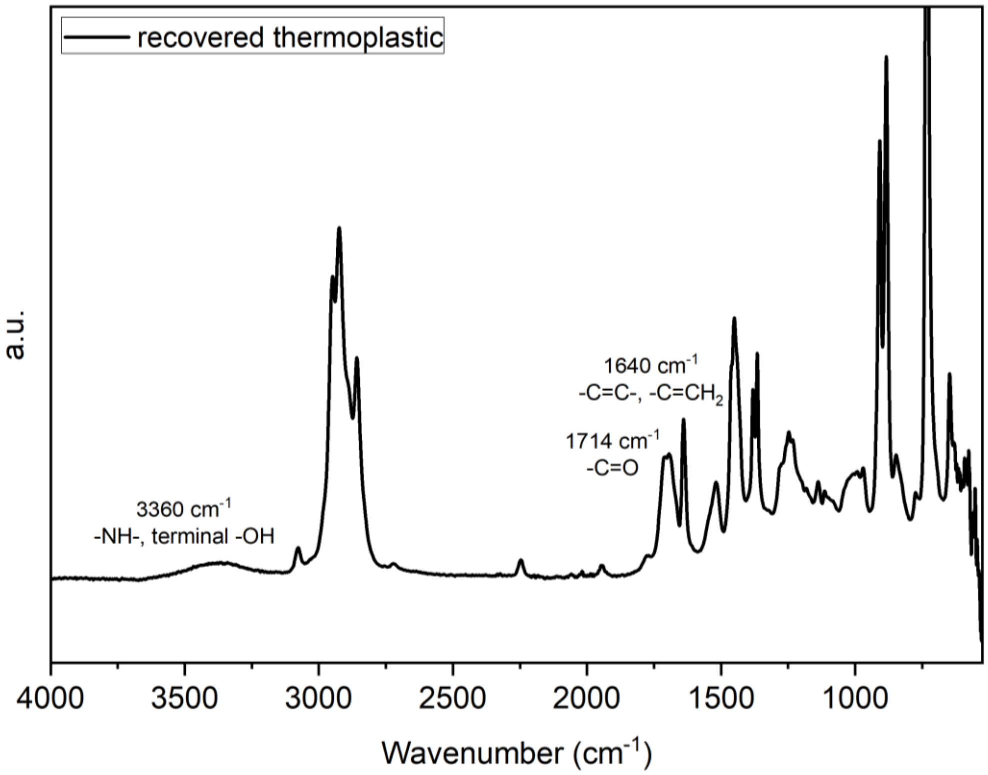

Figure S27C: Annotated ATR-IR spectrum of recovered thermoplastic material (M_n = 4.883 kg/mol, Đ = 4.0).

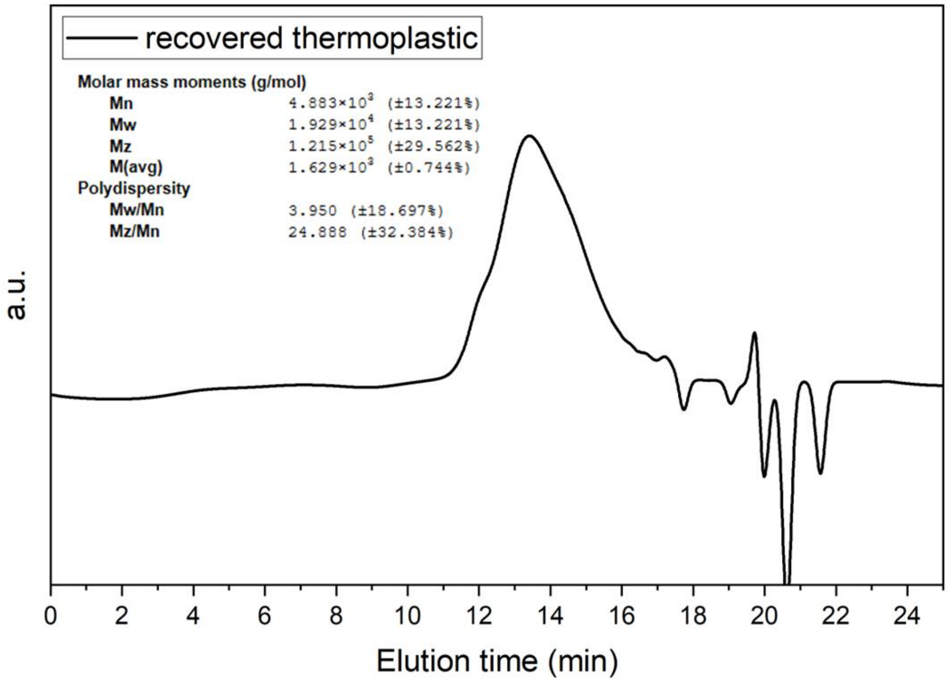

Figure S27D: GPC Chromatogram of recovered thermoplastic material (M_n = 4.883 kg/mol, Đ = 4.0).

The isolated thermoplastic HTPCR-polyHDI material was then crosslinked to polythioether thermoset material. 0.2637 mg (0.05406 mmol) of the thermoplastic was added to a 20 mL scintillation vial, after which 144 mg (0.3622 mmol, 6.7 equiv) of trithiol was added. A stock solution of 2 mg of DMPA in 10 mL of uninhibited THF was prepared, after which 1 mL of the stock solution was added to the vial containing thermoplastic and thiol. The vial was capped and agitated until dissolution of the contents was observed. The solution was then pipetted into a silicone dogbone mold and the mold was positioned approximately 10 inches away from a 275 W UV Sylvania Sunlamp and irradiated for 1 hour. The solidified thermoset identified as HTPCR-polyHDI-trithiol was removed from the pan using tweezers, and fragmentation of the dogbones was observed. The pieces of thin film were sonicated in acetone for 30 minutes to remove excess thiol. The formation of the thermoset and elimination of olefin content was confirmed by ATR-IR. The ATR-IR spectrum is given in Figure S28A. The glass transition and decomposition temperatures of the thermoset were determined by DSC and TGA, respectively. The annotated DSC spectrum is given in Figure S28B, and the TGA spectrum is given in S28C.

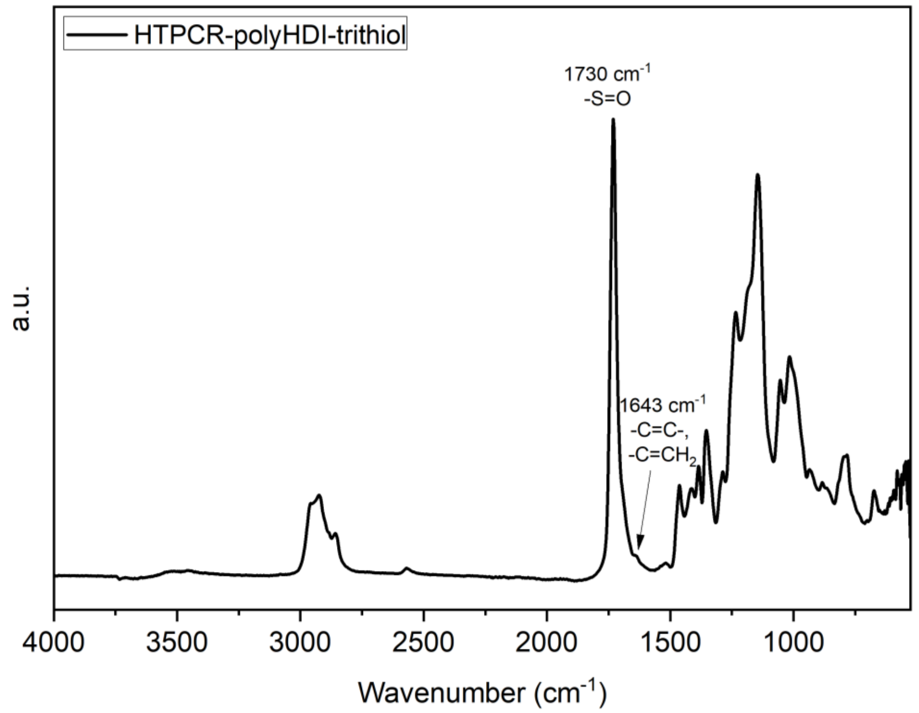

Figure S28A: Annotated ATR-IR spectrum of HTPCR-polyHDI-trithiol.

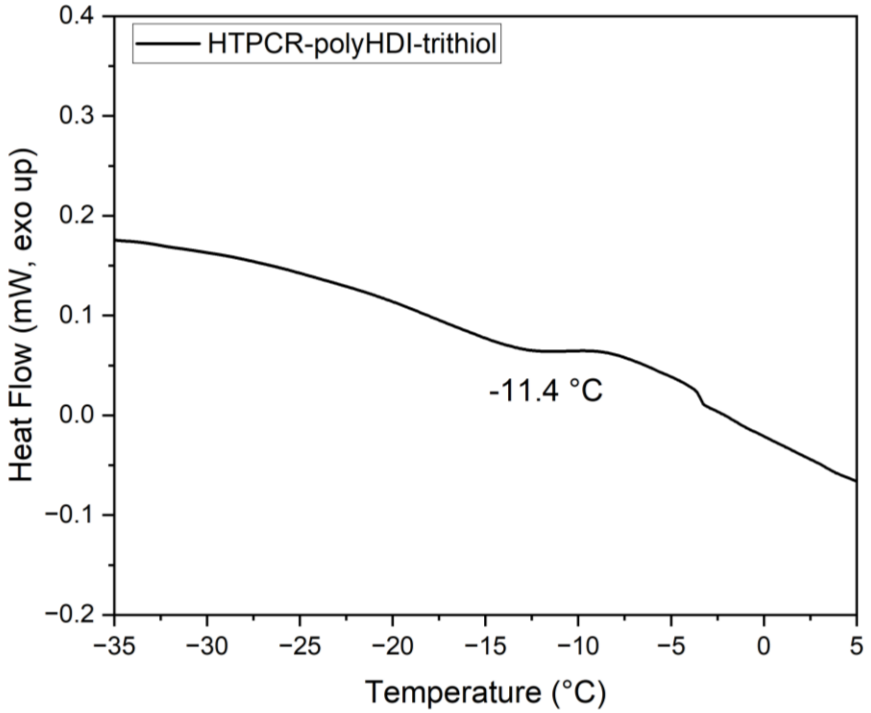

Figure S28B: Annotated DSC spectrum of HTPCR-polyHDI-trithiol used to elucidate the T_g.

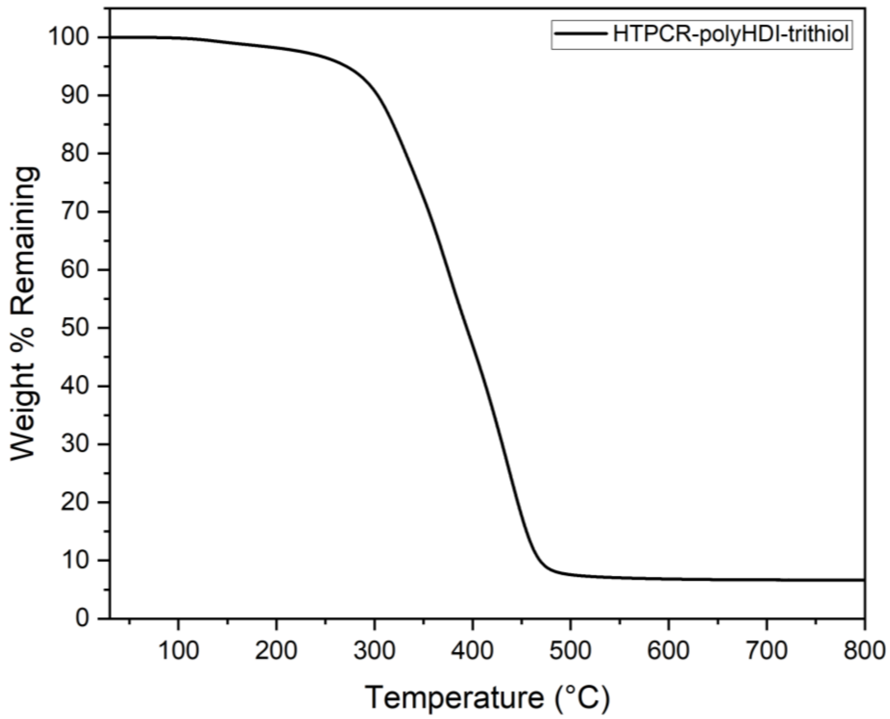

Figure S28C. Inert atmosphere TGA spectrum of HTPCR-polyHDI-trithiol.

V. References

1. Pangborn, A.; Giardello, M.; Grubbs, R.; Rosen, R.; Timmers, F. Safe and Convenient Procedure for Solvent Purification. *Organometallics* **1996**, *15*, 1518–1520.

2. Grau, E.; Mecking, S., Polyterpenes by ring opening metathesis polymerization of caryophyllene and humulene. *Green Chemistry* **2013**, *15*, 1112–1115.

3. Nachtrieb, K.; Nie, C.; Chirik, P.; Mohadjer Beromi, M. Synthesis, Thermochemistry, and Cure Behavior of Oligocyclobutane Prepolymers Relevant to Propellant Applications. *ACS Applied Polymer Materials* **2024**, *6*, 5171–5182.

4. Thomas, R.; Grubbs, R. H. Synthesis of Telechelic Polyisoprene via Ring-Opening Metathesis Polymerization in the Presence of Chain Transfer Agent. *Macromolecules* **2010**, *43*, 3705-3709.
